# Multi-layered target identification strategy of natural products: Daphnetin targets NQO1 and OPLAH in silicosis alleviation

**DOI:** 10.1016/j.jpha.2026.101629

**Published:** 2026-04-02

**Authors:** Ling Wang, Bo-Yan Ma, Sheng-Ping Jiang, Shu-Ting Wei, Xue-Mei Qin, Zhen-Yu Li

**Affiliations:** a Modern Research Center for Traditional Chinese Medicine, Shanxi University, Taiyuan, 030006, China; b The Key Laboratory of Chemical Biology and Molecular Engineering of Ministry of Education, Shanxi University, Taiyuan, 030006, China; c First Clinical Medical College, Shanxi Medical University, Taiyuan, 030000, China; d National Health Commission Key Laboratory of Pneumoconiosis, Shanxi Key Laboratory of Respiratory Diseases, Department of Pulmonary and Critical Care Medicine, The First Hospital of Shanxi Medical University, Taiyuan, 030000, China

**Keywords:** Silicosis, Daphnetin, Multi-layered target identification, NQO1, OPLAH

## Abstract

Pneumoconiosis is a common occupational lung disease with unclear pathogenesis and no effective targeted therapies. Emerging evidence suggests that daphnetin is a promising therapeutic candidate for silicosis, yet its efficacy targets remain poorly characterized. In this study, we propose and implement a multi-layered target identification strategy that integrates transcriptomics, clinical metabolomics, public database mining, an artificial intelligence (AI)-based target prediction model, and tissue-based thermal proteome profiling (Tissue-TPP) technology. This strategy enables stepwise elucidation, from pneumoconiosis-related targets to daphnetin efficacy targets and ultimately to direct targets. Using this approach, we validated nicotinamide adenine dinucleotide phosphate hydrogen (NAD(P)H) quinone oxidoreductase 1 (NQO1) and 5-oxoprolinase (OPLAH) as direct targets of daphnetin, and identified Kelch-like ECH-associated protein 1 (KEAP1), nuclear factor erythroid 2-related factor 2 (NRF2), and arginase 1 (ARG1) as indirect targets. Notably, OPLAH is reported here as a direct target of daphnetin for the first time. Furthermore, the binding of daphnetin to NQO1 and OPLAH helps explain its effects on cellular thiol levels. Using a thiol-responsive probe, we observed that daphnetin significantly increased intracellular thiol levels and reduced silica-induced reactive oxygen species (ROS) accumulation in both A549 and THP-1 cells, thereby contributing to its antioxidant effects. Additionally, overexpression (OE) of OPLAH significantly increased reduced glutathione (GSH) levels and alleviated silica-induced epithelial-mesenchymal transition (EMT). Conversely, OPLAH knockdown partially inhibited the efficacy of daphnetin. Together, these findings clarify the anti-fibrotic mechanism of daphnetin, support its therapeutic potential for silicosis, and provide a practical framework for the target discovery of natural products (NPs).

## Highlights

• We propose and implement a multi-layered strategy for identifying the targets of natural products.
• The antifibrotic effects of daphnetin are partially mediated by OPLAH.
• Daphnetin upregulates NQO1 through an NRF2-independent mechanism.
• OPLAH plays a key role in mediating daphnetin's regulation of thiol levels and ROS accumulation.

## Graphical abstract

**Figure:**
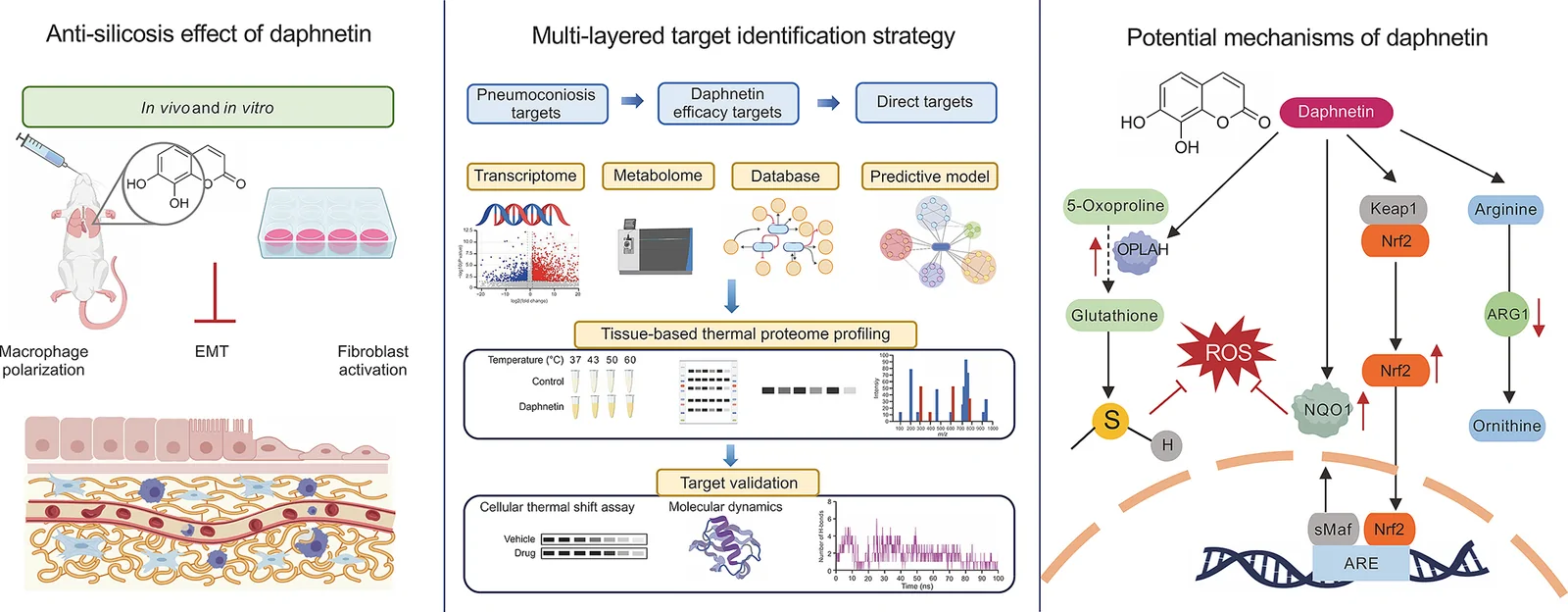

## 1 Introduction

Pneumoconiosis, one of the most prevalent occupational diseases worldwide, is a critical public health concern. It encompasses a series of occupational interstitial lung diseases induced by the inhalation of mineral dusts, such as free silica, asbestos fibers, coal dust, and other substances. Among these, silicosis is the most common and prototypical form [1]. Histopathologically, silicosis is characterized by irreversible and progressive pulmonary fibrosis. Its typical clinical symptoms include chest tightness, exertional dyspnea, and chronic cough, which significantly impair the quality of life [2]. To date, no specific or effective drug has been approved globally for the treatment of silicosis. In China, tetrandrine remains the only approved therapeutic agent for silicosis. Antifibrotic drugs developed for idiopathic pulmonary fibrosis, such as nintedanib and pirfenidone, are also used in the management of silicosis.

Natural products (NPs) have long served as a valuable source for innovative drug discovery due to their diverse chemical structures and significant biological activities [3]. Identifying their efficacy targets, including both direct and indirect targets, is crucial for elucidating their mechanisms of action and facilitating clinical translation. Direct targets are proteins that are directly bound by the compound to produce the desired phenotype, playing a key role in understanding the mode of action of NPs. In contrast, indirect targets are other proteins that are influenced by the direct targets, which in turn modulate the associated signaling pathways, gene expression, and metabolic networks [4]. In recent years, several NPs, such as curcumin [5], tanshinone IIA [6], and daphnetin [7], have demonstrated promising antifibrotic effects in silicosis animal models. However, a systematic understanding of their efficacy targets is still needed.

The emergence of transcriptomics, proteomics, and metabolomics has provided powerful tools for the rapid and systematic discovery of efficacy targets for NPs. Meanwhile, advances in computational science have enabled the development of online platforms that integrate existing omics data related to diseases and small molecules. For instance, the Gene Expression Omnibus (GEO) database archives gene expression data from global studies on disease and drug responses [8], the Comparative Toxicogenomics Database (CTD) maps gene/protein changes associated with environmental exposures [9], while GeneCards integrates multi-omics data to identify gene-disease associations [10]. Meanwhile, clinical metabolomics reflects real metabolic changes in the human body and is now widely used in early diagnosis and disease prediction. For NPs- and traditional Chinese medicine-related target discovery, several databases provide transcriptomic data under compound treatment, such as the Connectivity Map (CMap) [11], the Ingredients of Traditional Chinese medicine (ITCM) platform [12], and the HERB database [13]. Additionally, recent advances in big data and artificial intelligence (AI) have driven a paradigm shift in drug target discovery, from traditional experimental methods to data-driven strategies. For example, Xu et al. [14] proposed an NP virtual screening-interaction-phenotype strategy, which combines virtual screening, chemical proteomics, and metabolomics to explore and confirm the efficacy targets of NPs. Chen et al. [15] developed a deep learning model called Functional Representation of Gene Signatures (FRoGS), which predicts compound targets by projecting transcriptomic signatures onto their biological functions rather than relying solely on gene identities. Therefore, how to apply these databases and strategies to efficiently identify direct and indirect targets of NPs is a critical question that warrants further exploration.

In this study, we developed a multi-layered strategy for target identification by integrating transcriptomics, clinical metabolomics, public databases (GEO, CTD, GeneCards, CMap, HERB, and ITCM), an AI-based target prediction model, and tissue-based thermal proteome profiling (Tissue-TPP) technology. Using daphnetin as a case study, we systematically validated its anti-silicosis effects *in vitro* and *in vivo*, and identified its efficacy targets, categorizing them as direct or indirect. Subsequently, molecular docking, molecular dynamics (MD) simulations, and overexpression (OE)/knockdown studies at the cellular level, along with fluorescence assays, were conducted to further elucidate its mechanisms of action. This integrative approach provides a practical framework for identifying both direct and indirect targets of NPs and elucidates the mechanisms underlying daphnetin's alleviation of pneumoconiosis.

## 2 Materials and methods

### 2.1 Chemicals and materials

Silicon dioxide (99%, 0.5–10 μm, approximately 80% between 1–5 μm) was purchased from Sigma-Aldrich Laboratories, Inc. (St. Louis, MO, USA). Daphnetin (98% for animal experiments and 99% for cell experiments) was obtained from Chengdu Push Bio-Technology Co., Ltd. (Chengdu, China). Tetrandrine tablets were purchased from Zhejiang Jinhua CONBA Bio-Pharmaceutical Co., Ltd. (Jinhua, Zhejiang, China). Recombinant human transforming growth factor-beta 1 (TGF-β1) and ML385 were obtained from MedChemExpress (Shanghai, China) and Aladdin Inc. (Shanghai, China), respectively. Primary antibodies against fibronectin (FN) and collagen type I (COL-1) were purchased from Servicebio, Inc. (Wuhan, China). Alpha-smooth muscle actin (α-SMA), vimentin (VIM), arginase-1 (ARG1), Nicotinamide adenine dinucleotide phosphate hydrogen (NAD(P)H) quinone oxidoreductase 1 (NQO1), 5-oxoprolinase (OPLAH), and E-cadherin (E-cad) were purchased from Proteintech Group, Inc. (Wuhan, China). Kelch-like ECH-associated protein 1 (KEAP1) and nuclear factor erythroid 2-related factor 2 (NRF2) antibodies were obtained from Wanleibio (Shenyang, China). Chromatographic grade methanol (MeOH), aceto-nitrile (ACN), and formic acid (FA) were purchased from Thermo Fisher Scientific Co., Ltd (Waltham, MA, USA). Cholic acid-2,2,4,4-d4 (CA-d4), L-valine-d8 (Val-d8), L-phenylalanine (Ring-d5) (Phe-d5), and Stearic acid-18,18,18-d3 (FFA 18:0-d3) were purchased from MedChemExpress (Shanghai, China). Nonadecanoyl-2-hydroxy-snglycero-3-phosphocholine (LPC19:0) was purchased from Aladdin Bio-Chem Technology Co., Ltd. (Shanghai, China). Additional information on the reagents and antibodies used in this study is provided in Section S1 of the Supplementary data.

### 2.2 Animals experimental protocol

Specific pathogen-free (SPF) male rats (10 weeks, 180 to 220 g) were purchased from Beijing Vital River Laboratory Animal Technology Co., Ltd. (Certificate No.: 2016–0006; Beijing, China). All animals were housed under controlled conditions (20–22 °C, 45% ± 5% humidity, and a 12-h light/dark cycle) and had free access to food and water. The animal experiments were carried out in accordance with the guidelines for the Care and Use of Laboratory Animals of Shanxi University, China (Approval No.: SXULL2019014). After seven days of acclimatization, rats were randomly assigned to six groups (*n* = 9 per group): control, silicosis, low-dose (L), medium-dose (M), and high-dose (H) daphnetin (15, 30, and 60 mg/kg, respectively), and tetrandrine (30 mg/kg, positive control). Except for the control group, all rats received an intratracheal injection of silica (1 mL, 50 mg/mL) on Day 0. From Day 1, the treatment groups were orally administered their respective doses daily for 28 days, while the control and silicosis groups received saline. Respiratory rates were measured weekly to assess dyspnea induced by silica, and exercise capacity was evaluated using rotarod performance tests in the final week. On Day 29, rats were anesthetized with isoflurane and euthanized. The upper lobes of the right lungs were preserved in 4% paraformaldehyde for histopathological analysis, while the remaining lung tissues were frozen at −80 °C for subsequent analysis. The concentration of TGF-β1 in serum was quantified using a commercial enzyme-linked immunosorbent assay (ELISA) kit (Andy Gene, Beijing, China) according to the manufacturer's protocol. The protocols for hematoxylin-eosin (H&E), Masson's trichrome, Sirius red staining, and immunohistochemical staining of FN, α-SMA, COL-1, and VIM, as well as the corresponding quantification methods, are described in detail in Section S2 in the Supplementary data.

### 2.3 Detection of daphnetin and its metabolites

Daphnetin and its metabolites in lung tissue and serum samples were analyzed using a 1290 Infinity II liquid chromatography system (Agilent Technologies, Santa Clara, CA, USA) coupled with a TripleTOF 5600+ time-of-flight mass spectrometer (AB Sciex, Framingham, MA, USA). Detailed information is shown in Section S3 in the Supplementary data.

### 2.4 Cells experimental protocol

Human acute monocytic leukemia cells (THP-1) were obtained from the Cell Bank of the Chinese Academy of Sciences (Shanghai, China). Human lung adenocarcinoma cells (A549; Lot No.: TCH-C116) and human bronchial epithelial cells (BEAS-2B; Lot No.: TCH-C132) were sourced from Haixing Biosciences Co., Ltd. (Suzhou, China). Mouse embryonic fibroblasts (NIH 3T3; Lot No.: TM050) were purchased from Applied Biological Materials Co., Ltd. (Vancouver, Canada). THP-1 cells were cultured in Roswell Park Memorial Institute (RPMI) 1640 medium (Gibco™, Waltham, MA, USA), supplemented with 10% (*v/v*) fetal bovine serum (DCELL, Shanghai, China), 100 U/mL penicillin, and 100 U/mL streptomycin (Solarbio®, Beijing, China). Other cell lines were cultured in Dulbecco's modified Eagle's medium (DMEM) (Gibco™). Cells were cultured in a humidified incubator at 37 °C with 5% CO_2_. Experiments were conducted when cells reached approximately 80% confluence. For macrophage differentiation, THP-1 cells were seeded and treated with 20 nM phorbol myristate acetate (MedChemExpress, Shanghai, China) for 24 h. Stock solutions of 100 mM daphnetin and 20 mM ML-385 were prepared in dimethyl sulfoxide (DMSO) (Solarbio®), while TGF-β1 (2 μg/mL) and SiO_2_ (50 mg/mL) were dissolved in ultrapure water. These stock solutions were stored at −20 °C. Unless otherwise specified, the final concentrations of TGF-β1 and ML-385 were 10 ng/mL and 10 μM, respectively. Daphnetin was used at concentrations of 10 and 20 μM, while SiO_2_ concentrations were adjusted as follows: 100 μg/mL for THP-1 cells, 150 μg/mL for A549 cells, 250 μg/mL for BEAS-2B cells, and 200 μg/mL for NIH 3T3 cells. ML-385 was administered 1 h prior to daphnetin treatment, while the modeling agents TGF-β1 or SiO_2_ was applied concurrently with daphnetin.

### 2.5 Scratch wound healing assay

A sterile 200-μL pipette tip was used to create a cross-shaped scratch in the center of each well. After washing with phosphate-buffered saline (PBS) to remove debris, cells were treated with TGF-β1 alone or in combination with daphnetin. After treatment, the wells were washed with PBS, and wound closure was assessed by EVOS™ M5000 imaging system (Thermo Fisher Scientific Inc., Waltham, MA, USA). The cell migration rate was calculated as follows:

Migration rate (%) = Wound width at 0 h − wound width at 24 h/wound width at 0 h × 100%

### 2.6 Construction of pneumoconiosis gene expression signature

Transcriptomic data related to pneumoconiosis were obtained from the GEO database. Gene symbols were standardized, and gene lengths were retrieved using the biomaRt R package. For raw count datasets, expression values were converted to fragments per kilobase of transcript per million mapped reads. Datasets involving multiple timepoints or doses were split into simpler comparisons to minimize analytical bias. The Limma R package was used to analyze the data, excluding genes with over 20% zero values. Differentially expressed genes (DEGs) were identified using *P <* 0.05 and |log2(fold change (FC))| ≥ 1. Disease-related genes were gathered from CTD and GeneCards databases using keywords “pneumoconiosis” or “pulmonary fibrosis” and a median inference or relevance score as the inclusion criterion. The overlap between DEGs and disease-related genes was used to construct the pneumoconiosis gene expression signature. Reactome pathway enrichment analyses were performed using the Enrichr online platform and visualized with R.

### 2.7 Construction of daphnetin gene expression signature

Gene expression profiles and *Z*-score data for daphnetin (10 μM) treatment in A375, A549, HA1E, HCC515, HT29, MCF7, PC3, and VCAP cell lines were obtained from the CMap platform, with DEGs filtered using |*Z*-score| ≥ 2. Additionally, RNA sequencing (RNA-seq) data for A2780 and SKOV3 cells treated with daphnetin for 24 h (GSE267812) were retrieved from the GEO database. No transcriptomic data for daphnetin are available in the HERB and ITCM databases. DEGs analysis was conducted using R, with *P* < 0.05 and |log2FC| ≥ 2 as the thresholds. Identified genes were categorized by “DEGs-Cell line-treatment (duration/concentration)”, and genes with a frequency of appearance ≥3 across these analyses were defined as the daphnetin expression signature.

### 2.8 Prediction of daphnetin targets by FRoGS model

Based on previously published literature [15], the corresponding Python code and pretrained gene embedding vectors were obtained from the online platform GitHub (https://github.com/chenhcs/FRoGS). Using the compound identifier (BRD-K61269089) as input, the code was executed to predict targets of daphnetin across multiple cell lines in the complementary DNA (cDNA) and short hairpin RNA (shRNA) models. Targets with a score ≥ 0.95 were selected, and duplicates were removed to obtain the final predicted targets of daphnetin.

### 2.9 Collecting differential metabolites and corresponding metabolic enzymes from clinical pneumoconiosis metabolomics

Clinical metabolomics studies on pneumoconiosis were retrieved from the PubMed (https://pubmed.ncbi.nlm.nih.gov/) and ScienceDirect (https://www.sciencedirect.com/) databases using the keywords “pneumoconiosis” and “metabolomics”. Differential metabolites were manually curated, and their Kyoto Encyclopedia of Genes and Genomes identifiers (KEGG IDs) were obtained using the KEGGREST R package complemented by manual annotation. Metabolites with inconsistent trends across studies were excluded. Metabolite-enzyme interaction networks were constructed using the Metscape plugin in Cytoscape (version 3.6.1) to identify the associated metabolic enzymes. KEGG pathway enrichment analyses were performed using the Enrichr online platform and visualized with R.

### 2.10 Identification of metabolic enzymes associated with daphnetin regulation via pseudotargeted metabolomics analysis

Lung tissue sample preparation, chromatographic conditions, and mass spectrometry parameters are shown in Section S4 in the Supplementary data. Comprehensive pseudotargeted metabolomics data were acquired using an AB SCIEX Triple Quad 5500 linear ion trap triple quadrupole mass spectrometer (AB Sciex) coupled to an ExionLC AD UHPLC system (Shimadzu, Kyoto, Japan), employing a scheduled multiple reaction monitoring (MRM) mode. The amino acid metabolomic profiling analysis was performed using an AB SCIEX Triple Quad 3200 linear ion trap triple quadrupole mass spectrometer (AB Sciex) coupled to an ExionLC AD UHPLC system, employing MRM mode. Details of retention time, Q1/Q3 ions, declustering potential (DP), and collision energy (CE) for each metabolite are listed in Table S1 (comprehensive pseudotargeted metabolomics) and Table S2 (amino acid). Data were processed using SCIEX OS 3.0.3 software (AB Sciex) with peak areas normalized using internal standards. Metabolites with a relative standard deviation (RSD) greater than 30% in quality control (QC) samples were excluded. Differential metabolites in silicosis were identified based on *P* < 0.05 and FC > 1.2 or FC < 0.8, and those responsive to daphnetin treatment were further filtered using the same criteria. Heatmaps were generated using the ComplexHeatmap package in R. KEGG pathway enrichment analyses were performed using the MetaboAnalyst6.0 online platform. Additionally, batch effect correction was performed using the ComBat method from the sva package in R. The efficacy of the correction was evaluated using principal component analysis (PCA) of QC samples. KEGG IDs of daphnetin-regulated metabolites were then retrieved to construct the metabolite-enzyme network and identify the associated metabolic enzymes.

### 2.11 Tissue-TPP

This experiment was performed based on previously published methods with appropriate modifications [16,17].

#### 2.11.1 In vivo thermal shift assays

Daphnetin (0 or 20 mg/kg) was suspended in 1.0% carboxy methyl cellulose and orally administered to adult male rats. 2 h post-administration, rats were euthanized, and lung tissues were harvested. Tissues were minced on ice and homogenized in pre-cooled PBS containing 1% protease and phosphatase inhibitors. Homogenates were aliquoted and heated at 37.0, 47.7, 51.1, 54.2, 57.2, 60.7, or 67.0 °C for 3 min using a polymerase chain reaction (PCR) thermal cycler (T960; Heal Force, Hangzhou, China), then cooled at room temperature for 3 min. Aggregated proteins were removed by centrifugation, and the supernatants (90 μL) were collected and boiled with 1× loading buffer.

#### 2.11.2 In vitro thermal shift assays

Lung tissues from untreated rats were processed similarly. Lysates were incubated with 100 μM daphnetin or DMSO at 37 °C for 2 h, followed by the same heating protocol described above.

#### 2.11.3 Protein identification

Heat-treated samples were resolved on 8% sodium dodecyl sulfate-polyacrylamide gel electrophoresis (SDS-PAGE) gels, stained with Coomassie blue overnight, and imaged (ChemiDoc™ XRS+; Bio-Rad, Hercules, CA, USA). Differential bands showing altered thermal stability were excised and subjected to liquid chromatography-tandem mass spectrometry (LC-MS/MS) (Shanghai Applied Protein Technology Co., Ltd., Shanghai, China). Proteins with an identification score > 100 and matching molecular weights were considered potential daphnetin-binding targets [17].

### 2.12 Cellular thermal shift assay (CETSA)

Following the Tissue-TPP assay, lung tissues from rats that had received oral daphnetin or undergone *in vitro* incubation were subjected to Western blot analysis to validate target proteins. The melting temperature (*T*_m_) values were then determined by sigmoid curve fitting, and the thermal shift (*ΔT*_m_) was calculated by comparing the daphnetin-treated group with the control group.

### 2.13 Western blot analysis

The detailed protocol for Western blot is shown in Section S5 in the Supplementary data.

### 2.14 Quantitative real-time PCR

Detailed procedures for quantitative real-time PCR and the primer sequences for *NRF2* and *NQO1* are shown in Table S3 and Section S6 in the Supplementary data, respectively.

### 2.15 Molecular docking, MD simulations and virtual mutagenesis

#### 2.15.1 Protein structure evaluation

The three-dimensional (3D) crystal structure of NQO1 protein (Protein Data Bank (PDB) ID: 8RFN) was retrieved and downloaded from the RCSB PDB (http://www.rcsb.org) in PDB format (∗.pdb). However, as the crystal structure of OPLAH protein has not been determined through X-ray diffraction, its structure predicted by AlphaFold DB (ID: O14841) was used for subsequent experiments. The structural quality of both proteins was assessed using the SAVES 6.0 web server (https://saves.mbi.ucla.edu/).

#### 2.15.2 Molecular docking and MD simulations

Molecular docking was performed using AutoDock Vina (La Jolla, CA, USA), as described in the previous study [18]. MD simulations were conducted using GROMACS 2022.2 (http://www.gromacs.org). Detailed information regarding the molecular docking and MD simulations methods is shown in Section S7 in the Supplementary data.

#### 2.15.3 Virtual alanine scanning analysis

To evaluate the contribution of key binding residues identified by molecular docking, virtual alanine scanning was conducted on the OPLAH-daphnetin complex. This analysis included both single-point mutations and combined mutations at the key binding sites. The resulting changes in binding free energy (*ΔΔG*) were calculated and analyzed to assess the impact of these mutations [19].

### 2.16 Plasmid construction and transfection

The OPLAH OE plasmid (pLenti-GI-CMV-Cterm-3xFlag) and its corresponding negative control (OE-NC), as well as the OPLAH knockdown plasmid (pLenti-U6-shRNA-CMV-GFP-2A-Puro) and its corresponding negative control (sh-NC), were synthesized by Applied Biological Materials Co., Ltd. For transfection, A549 cells were transfected with the plasmids using the Seven HighTrans™ DNA non-liposomal transfection reagent (Seven, Beijing, China). Briefly, 1 μg of plasmid was diluted in 50 μL of Opti-MEM® reduced serum medium (Gibco™), and mixed with 2 μL of transfection reagent diluted in the same medium. The mixture was incubated at room temperature for 5 min, followed by a further 20 min incubation. The resulting DNA-transfection reagent complexes were then added to A549 cells that had been serum-starved for 4 h. After incubating for 48 h, the cells were washed with PBS and subsequently exposed to silica alone or in combination with daphnetin for 24 h before being collected for subsequent analysis.

### 2.17 Intracellular reactive oxygen species (ROS) detection

Intracellular ROS levels were assessed using a ROS assay kit (Wanleibio, Shenyang, China). After treatment with daphnetin for 24 h, cells were incubated with 10 μM dichloro-dihydro-fluorescein diacetate (DCFH-DA) probe at 37 °C in the dark for 20 min. The Hoechst 33342 probe (5 μg/mL) (Servicebio, Wuhan, China) was then added and incubated for 5 min. Images were captured under the EVOS™ M5000 imaging system, and the average fluorescence intensity was analyzed using ImageJ software.

### 2.18 Intracellular thiol detection using CM-Mit probe

The CM-Mit probe was kindly provided by Prof. Kai-Qing Ma (Shanxi University, Taiyuan, China) [20]. After different treatments, cells were incubated with 10 μM CM-Mit probe at 37 °C in the dark for 20 min, and then with 5 μg/mL Hoechst 33342 probe for 5 min. After washing with PBS, cells were imaged using the EVOS™ M5000 imaging system. The average fluorescence intensity was quantified using ImageJ software.

### 2.19 Intracellular reduced glutathione (GSH) detection

Intracellular GSH levels were measured according to the manufacturer's instructions using a commercial assay kit (Cat. No. WLA105a, Wanleibio, Shenyang, China).

### 2.20 Statistical analysis

Data analysis was conducted using Prism version 8.0 (GraphPad, La Jolla, CA, USA). Results are presented as mean ± standard deviation (SD). Statistical significance was determined using two-tailed Student's *t*-tests for pairwise comparisons, while one-way analysis of variance (ANOVA) was utilized for comparisons among multiple groups. A *P*-value less than 0.05 was considered to be statistically significant.

## 3 Results

### 3.1 Daphnetin ameliorated silica-induced pulmonary fibrosis

**Fig. 1:**
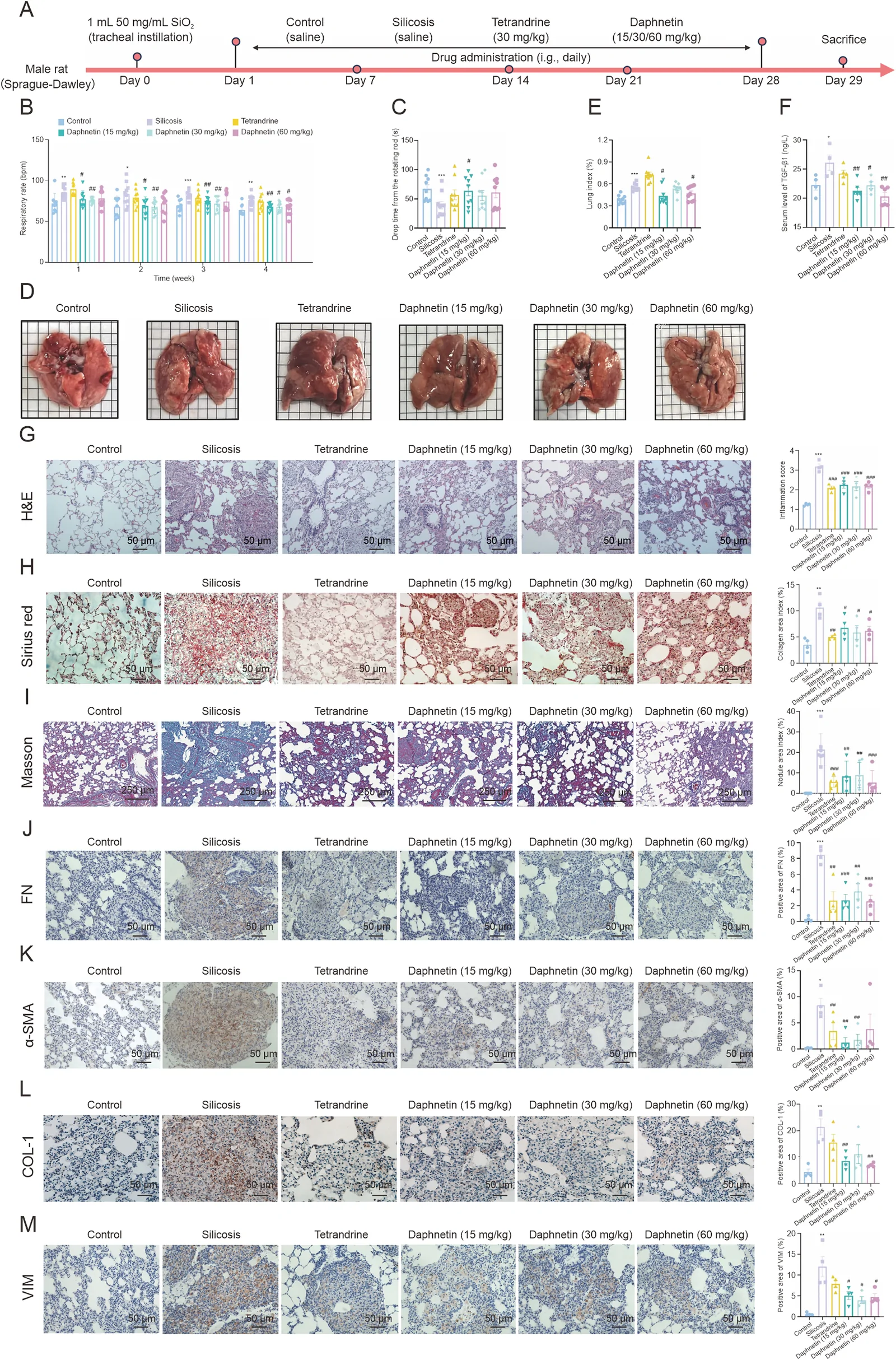
Daphnetin alleviates silica-induced histopathological damage in rat lung tissues. (A) Schematic representation of the animal experimental protocol. (B) Respiratory rate measurements (*n* = 9). (C) Rotarod test (*n* = 9). (D) Representative macroscopic images of lungs (*n* = 3). (E) Lung index (*n* = 9). (F) The serum levels of transforming growth factor-beta 1 (TGF-β1) (*n* = 6). (G) Hematoxylin-eosin (H&E) staining (left) and corresponding inflammation scores (right) (*n* = 4). (H) Sirius red staining (left) and semi-quantitative analysis of collagen area (right) (*n* = 4). (I) Masson's trichrome staining and quantitative analysis of nodule area (*n* = 4). (J–M) Immunohistochemical staining (left) and semi-quantification of fibrosis-related proteins (right) (*n* = 4): fibronectin (FN) (J), alpha-smooth muscle actin (α-SMA) (K), type I collagen (COL-1) (L), and vimentin (VIM) (M). ^∗^*P* < 0.05, ^∗∗^*P* < 0.01, and ^∗∗∗^*P* < 0.001 vs. control group; ^#^*P* < 0.05, ^##^*P* < 0.01, and ^###^*P* < 0.001 vs. silicosis group.

To evaluate the therapeutic potential of daphnetin against silicosis, a rat model was established via a single intratracheal instillation of silica suspension, as illustrated in Fig. 1A. The body weight (Fig. S1A) and food intake (Fig. S1B) of rats were evaluated and showed no significant differences among groups. Additionally, previous studies have reported that patients with silicosis commonly exhibit dyspnea, wheezing, and reduced exercise capacity [2]. Accordingly, respiratory rate and motor performance were assessed. As shown in Fig. 1B, silica exposure significantly increased the respiratory rate of rats from Week 1 to Week 4. Treatment with daphnetin (15, 30, and 60 mg/kg) effectively alleviated dyspnea, with efficacy superior to that of tetrandrine. Furthermore, the rotarod test revealed a significant reduction in retention time in silicotic rats compared to controls, which was significantly ameliorated by 15 mg/kg daphnetin treatment (Fig. 1C). Pulmonary anatomical examination showed that lungs from the silicosis group exhibited rough surfaces with prominent white or grayish-white nodules, whereas the daphnetin-treated groups showed obvious improvement (Fig. 1D). In addition, silica exposure significantly increased the lung index, and this increase was markedly reduced by treatment with 15 and 60 mg/kg daphnetin (Fig. 1E). Moreover, the serum levels of TGF-β1, a key profibrotic cytokine, were markedly elevated in silicosis rats and significantly suppressed by daphnetin (Fig. 1F).

To evaluate the effect of daphnetin on the fibrotic changes in lung tissues, H&E, Sirius red, and Masson's trichrome staining were performed. As shown in Fig. 1G, H&E staining revealed severe pathological changes in the silicosis group, characterized by alveolar thickening, structural destruction, foamy macrophage aggregation, and tracheal compression. Both tetrandrine and daphnetin treatments markedly alleviated nodule formation and improved lung architecture. Ashcroft scoring based on H&E staining confirmed significantly lower pathological scores in all treated groups. Sirius -red staining showed that tetrandrine and daphnetin markedly reduced silica-induced collagen deposition (Fig. 1H). Subsequently, Masson's staining demonstrated that both treatments significantly reduced silica-induced pulmonary nodule formation, with the greatest reduction in nodule area observed in the 60 mg/kg daphnetin group (Fig. 1I). Immunohistochemical analysis revealed elevated expression of markers associated with fibrosis and epithelial-mesenchymal transition (EMT), including FN (Fig. 1J), α-SMA (Fig. 1K), COL-I (Fig. 1L), and VIM (Fig. 1M), which were primarily localized within silicotic nodules. These proteins were significantly downregulated by all doses of daphnetin, with efficacy comparable to that of tetrandrine.

In summary, daphnetin at all tested doses effectively ameliorated silica-induced pulmonary dysfunction, structural damage, fibrosis, and EMT *in vivo*. Notably, 15 mg/kg daphnetin exhibited the greatest effect in relieving dyspnea, reducing the lung index, and improving motor performance. Its anti-fibrotic efficacy, especially in reducing collagen deposition, was comparable to that of tetrandrine (30 mg/kg), highlighting daphnetin's strong therapeutic potential for silicosis.

### 3.2 Daphnetin attenuated silica- and TGF-β1-induced EMT and fibroblast activation *in vitro*

**Fig. 2:**
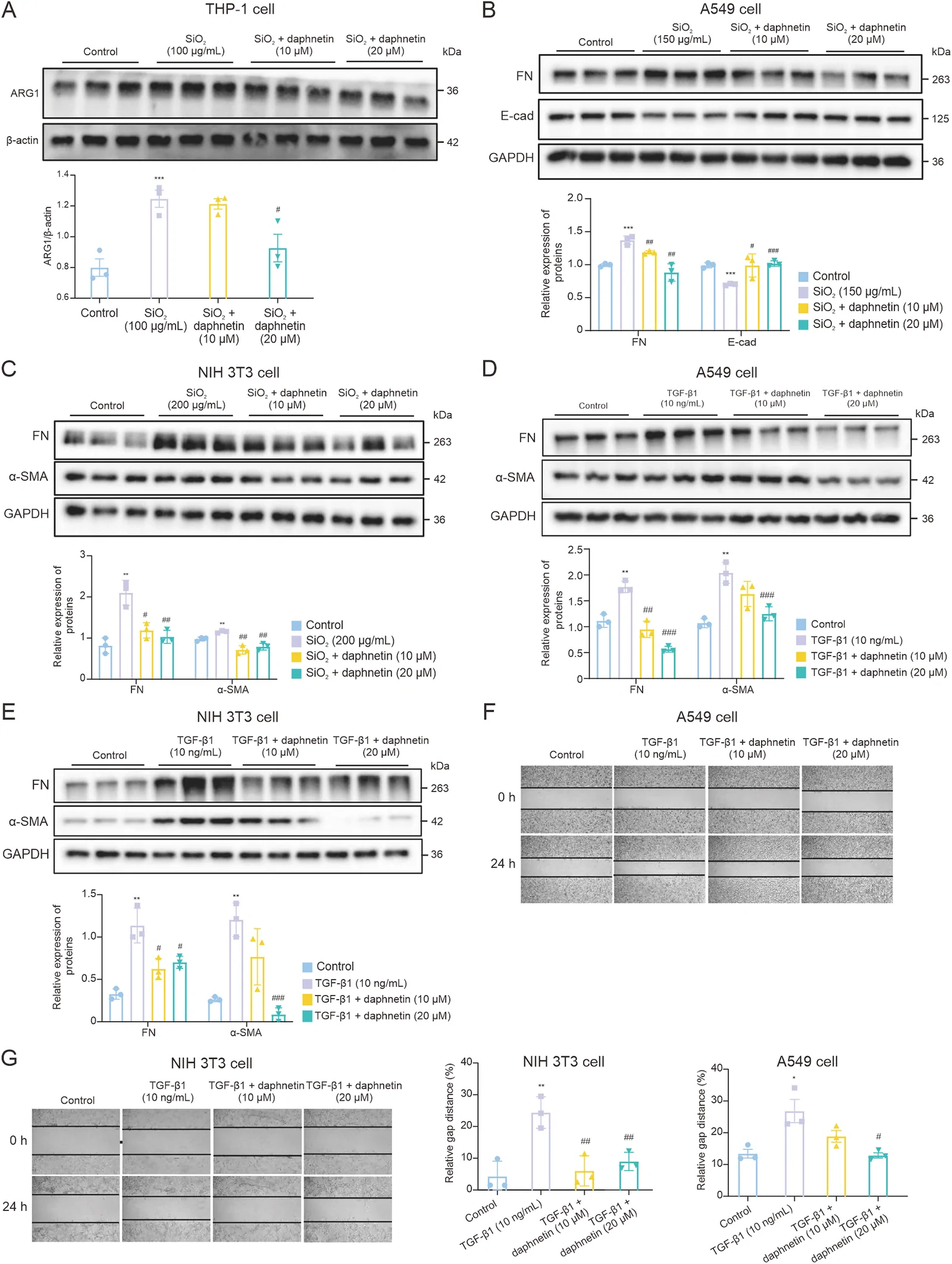
The effect of daphnetin on macrophage polarization, epithelial-mesenchymal transition (EMT), and fibroblast activation (*n* = 3). (A) Arginase 1 (ARG1) expression in THP-1 cells exposed to silica (100 μg/mL). (B) Fibronectin (FN) and E-cadherin (E-cad) expression in A549 cells exposed to silica (150 μg/mL). (C) FN and alpha-smooth muscle actin (α-SMA) expression in NIH 3T3 cells exposed to silica (200 μg/mL). (D, E) FN and α-SMA expression in A549 (D) and NIH 3T3 (E) cells treated with transforming growth factor beta 1 (TGF-β1) (10 ng/mL). (F, G) The scratch wound healing assay in A549 (F) and NIH 3T3 (G) cells treated with TGF-β1. ^∗^*P* < 0.05, ^∗∗^*P* < 0.01, and ^∗∗∗^*P* < 0.001 vs. control group; ^#^*P* < 0.05, ^##^*P* < 0.01, and ^###^*P* < 0.001 vs. SiO_2_ group. GAPDH: glyceraldehyde 3-phosphate dehydrogenase.

M2 macrophage polarization, EMT, and fibroblast activation are key processes in pneumoconiosis. Silica exposure not only induces EMT and fibroblast activation [21], but also promotes TGF-β1 release by M2 macrophages, further amplifying these fibrotic processes [22]. To determine whether orally administered daphnetin could be distributed in the lung tissue and exert anti-fibrotic effects, LC-MS/MS analysis was performed, followed by *in vitro* experiments to assess its inhibitory effects on the above key processes in pneumoconiosis. As illustrated in Figs. S1C–H, daphnetin (*m/z* 177.0188) was detected in both serum and lung tissue, confirming pulmonary distribution, and the metabolites daphnetin-*O*-methyl (191.0350) and daphnetin-*O*-sulfonate (257.1794) were also found. We then evaluated daphnetin's effect on macrophage polarization in THP-1 cells and found that daphnetin inhibited silica-induced ARG1 upregulation, a marker of M2 polarization (Fig. 2A). The anti-fibrotic effects of daphnetin were then assessed using A549 cells, BEAS-2B epithelial cells, and NIH 3T3 fibroblasts. Silica exposure significantly increased FN levels and decreased E-cad expression in A549 (Fig. 2B) and BEAS-2B cells (Figs. S2A and B), which was reversed by daphnetin in a dose-dependent manner. Daphnetin also corrected FN and α-SMA expression abnormalities in NIH 3T3 fibroblasts (Fig. 2C). To further confirm daphnetin's effects, TGF-β1 (10 ng/mL) was used to induce fibrosis. TGF-β1 significantly upregulated FN and α-SMA expression in A549 (Fig. 2D), BEAS-2B (Figs. S2C and D), and NIH 3T3 cells (Fig. 2E), while daphnetin (10 and 20 μM) significantly reduced these levels. Additionally, scratch assays showed that daphnetin inhibited TGF-β1-induced cell migration in a dose-dependent manner in A549 (Fig. 2F) cells, BEAS-2B (Fig. S2E) cells, and NIH 3T3 fibroblasts (Fig. 2G), reducing EMT and fibroblast activation as evidenced by a lower wound healing rate.

### 3.3 A multi-omics and AI-driven hierarchical strategy for targets identification of daphnetin in silicosis

**Fig. 3:**
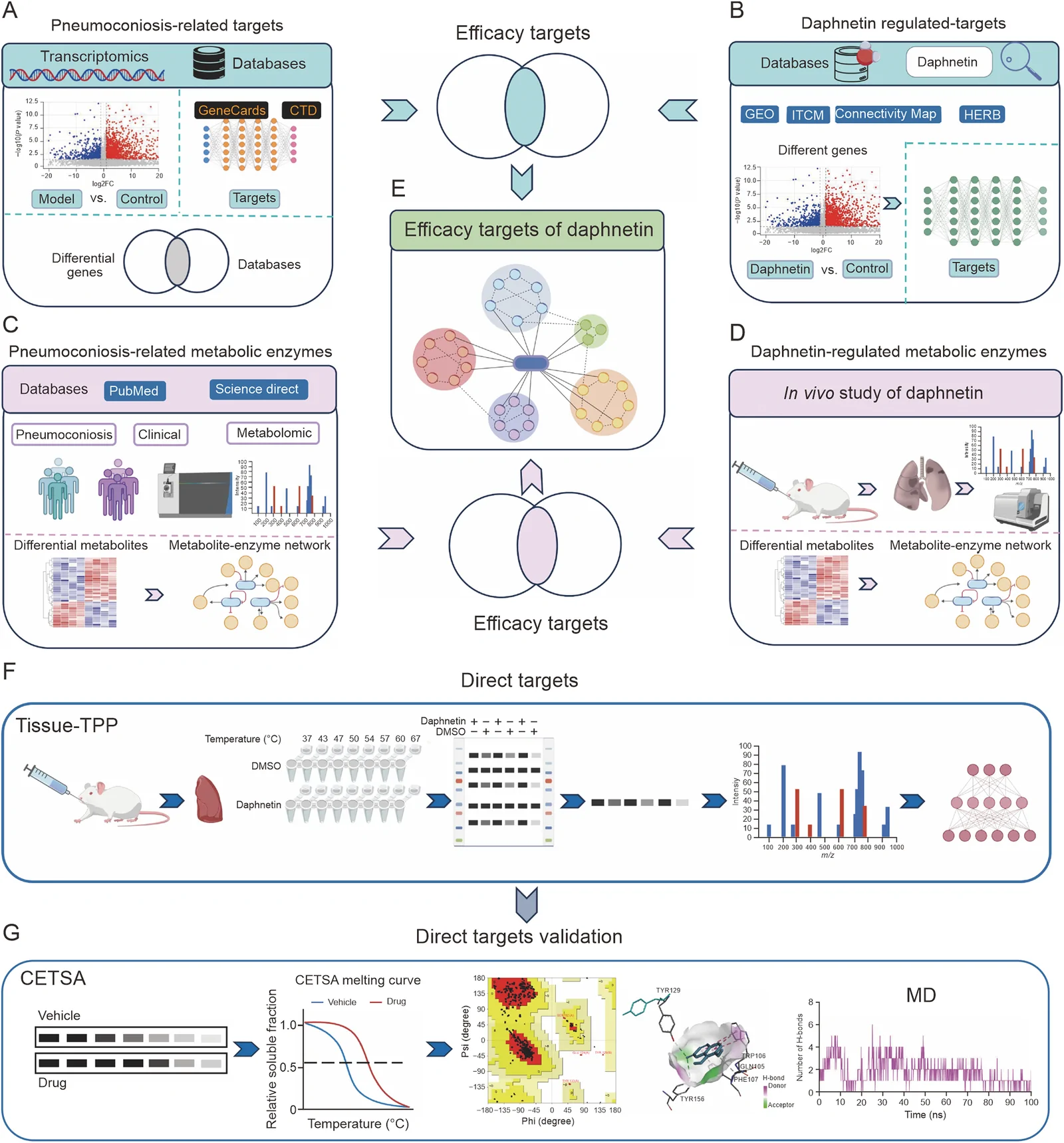
The multi-layered strategy for natural products (NPs) target identification. (A) Construction of a pneumoconiosis gene expression signature. (B) Identification of candidate efficacy targets of daphnetin using gene expression signatures. (C) Construction of a metabolite-enzyme association network for pneumoconiosis. (D) Identification of metabolomics-derived efficacy targets of daphnetin. (E) Integration of daphnetin efficacy targets. (F) Identification of direct targets using tissue-based thermal proteome profiling (Tissue-TPP). (G) Validation and mechanistic characterization of the direct targets. FC: fold change; CTD: Comparative Toxicogenomics Database; GEO: Gene Expression Omnibus; ITCM: Ingredients of traditional Chinese medicine; DMSO: dimethyl sulfoxide; CETSA: cell thermal shift assay; MD: molecular dynamics.

Although *in vivo* and *in vitro* studies have confirmed the therapeutic effects of daphnetin in silicosis, its efficacy targets remain unclear. Accordingly, we proposed a multi-layered strategy for NPs target identification by integrating transcriptomics, clinical metabolomics, public databases, AI techniques, and Tissue-TPP, which achieved the stepwise identification from disease targets to efficacy targets, and ultimately to direct and indirect targets of NPs (Fig. 3). Specifically, this strategy comprises nine steps: i) constructing a pneumoconiosis gene signature, ii) identification of daphnetin candidate efficacy targets from transcriptomics, iii) collecting differential metabolites and corresponding metabolic enzymes from clinical pneumoconiosis metabolomics, iv) identification of daphnetin candidate efficacy targets from metabolomics, v) integrating potential efficacy targets of daphnetin from steps ii) and iv), vi) identification of direct targets via Tissue-TPP, and vii) validating direct targets through CETSA and MD simulations.

### 3.4 Multi-layered strategy for identifying daphnetin targets in silicosis via multi-omics and AI integration

#### 3.4.1 Construction of pneumoconiosis gene expression signature

**Fig. 4:**
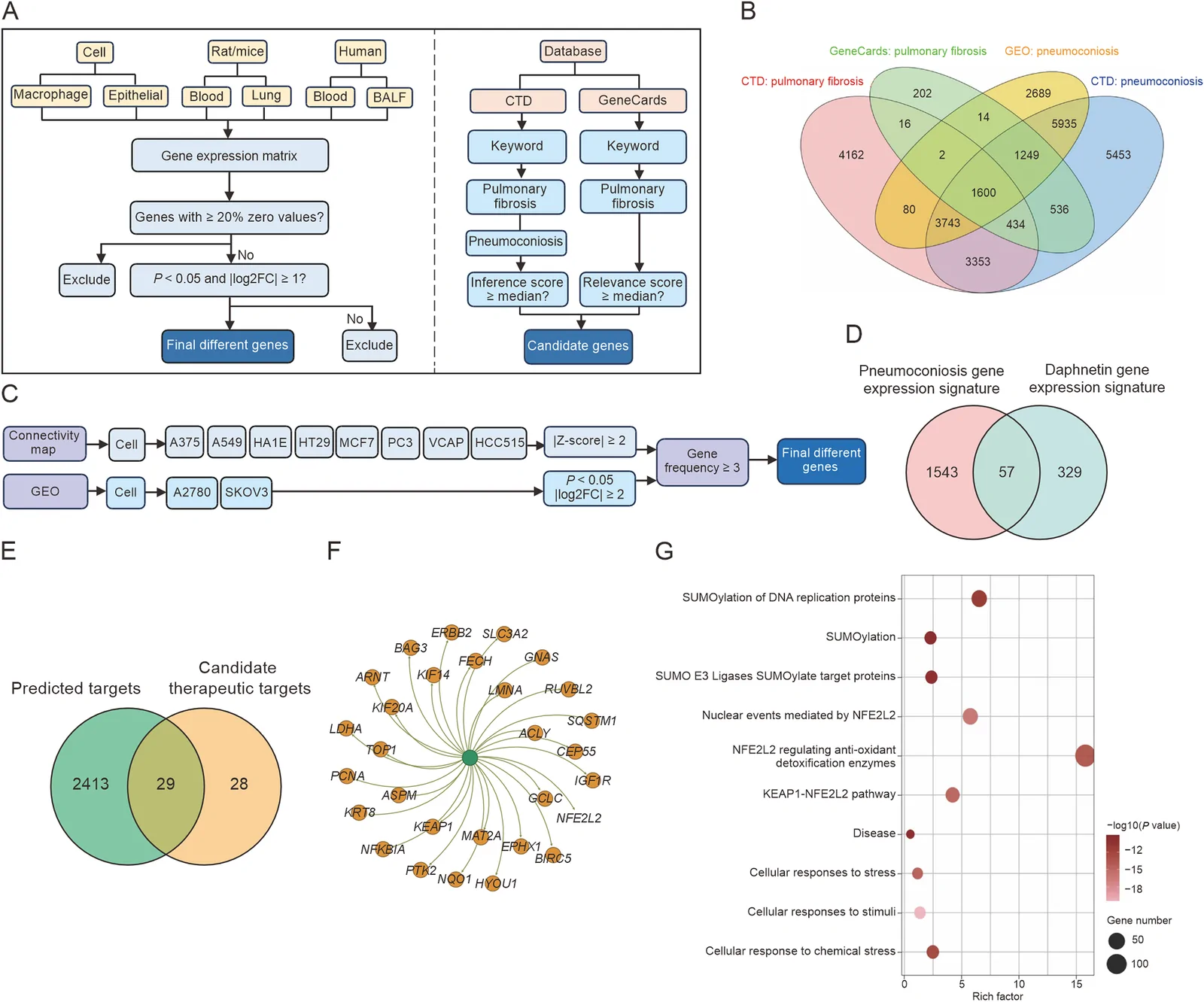
Identification of daphnetin's efficacy targets through transcriptome data analysis. (A) Workflow for constructing the pneumoconiosis gene expression signature. (B) Venn diagram of pneumoconiosis-related genes from different sources. (C) Workflow for constructing the daphnetin-associated gene expression signature. (D) Venn analysis between daphnetin-associated targets and pneumoconiosis-related targets. (E) Venn analysis between the 57 candidate targets and Functional Representation of Gene Signatures (FRoGS)-predicted targets. (F) Network diagram of 29 overlapping efficacy targets. (G) Reactome pathway enrichment analysis of the 29 efficacy targets. BALF: bronchoalveolar lavage fluid; FC: fold change; CTD: Comparative Toxicogenomics Database; GEO: Gene Expression Omnibus; NFE2L2: nuclear factor erythroid 2-related factor 2; KEAP1: Kelch-like ECH-associated protein 1.

The construction process of the pneumoconiosis gene expression signature is illustrated in Fig. 4A. A total of 41 transcriptomic datasets related to pneumoconiosis were retrieved from the GEO database (Table S4), including clinical samples (alveolar lavage fluid (BALF) and blood from pneumoconiosis patients), animal samples (blood and lung tissues from rats or mice), and cellular samples (epithelial cells and macrophages). After preprocessing and differential analysis using R packages, 15,312 potential pneumoconiosis-related genes were identified using a threshold of *P* < 0.05 and |log2FC| ≥ 1. Additionally, 13,389 pneumoconiosis-related and 22,303 pulmonary fibrosis-related genes were obtained from the CTD database, along with 4052 pulmonary fibrosis-related genes from GeneCards. Intersecting these gene sets resulted in 1600 genes, which defined the final pneumoconiosis gene signature (Fig. 4B and Table S5).

#### 3.4.2 Identification of daphnetin candidate efficacy targets from transcriptomics and the FRoGS model

The construction of the daphnetin gene expression signature is illustrated in Fig. 4C. A total of 16 transcriptomic datasets were obtained from the CMap database and 4 additional datasets from the GEO database. Genes with |*Z*-score| ≥ 2 (CMap) or |log2FC| ≥ 2 (GEO) and *P*-value < 0.05 were defined as daphnetin-associated DEGs. After combining all DEGs, 1264 DEGs were identified (Table S6). These were then organized according to the “Gene-Cell line-treatment” structure, and 386 genes with a frequency ≥ 3 were defined as the daphnetin gene expression signature (Table S7). Intersection analysis between these 386 genes and 1600 pneumoconiosis-associated genes yielded 57 overlapping targets, which were proposed as candidate efficacy targets of daphnetin (Figs. 4D and S3A).

To further focus on the most relevant efficacy targets of daphnetin, we used the FRoGS model to predict daphnetin targets. As shown in Fig. S3B, FRoGS learns high-level gene function features by integrating perturbation-based transcriptomic profiles from compound treatments (e.g., CMap) and from genetic perturbations, including shRNA-mediated knockdown and cDNA-mediated OE, enabling it to distinguish key targets from downstream effectors and improve prediction accuracy. After applying a score threshold of ≥0.95, 1412 and 1566 potential targets were identified from the cDNA and shRNA datasets, respectively. Further merging and deduplicating yielded 2442 predicted targets (Table S8). Intersecting these genes with the 57 candidate targets yielded 29 overlapping targets (Fig. 4E), such as *NQO1*, *NRF2*, Kelch-like ECH-associated protein 1 (*KEAP1*), glutamate-cysteine ligase catalytic subunit (*GCLC*), and baculoviral IAP repeat-containing 5 (*BIRC5*) (Fig. 4F). Reactome pathway enrichment analysis revealed that KEAP1-NRF2 pathway activation, NRF2-dependent nuclear transcriptional events, and NRF2-governed antioxidant/detoxification enzyme were probably related to daphnetin's anti-pneumoconiosis effects (Fig. 4G). Notably, the KEAP1-NRF2-NQO1 pathway, a critical axis for cellular defense against oxidative stress, has been demonstrated by multiple studies to participate in fibrotic progression [23].

#### 3.4.3 Identification of differential metabolites and corresponding metabolic enzymes from clinical pneumoconiosis metabolomics

**Fig. 5:**
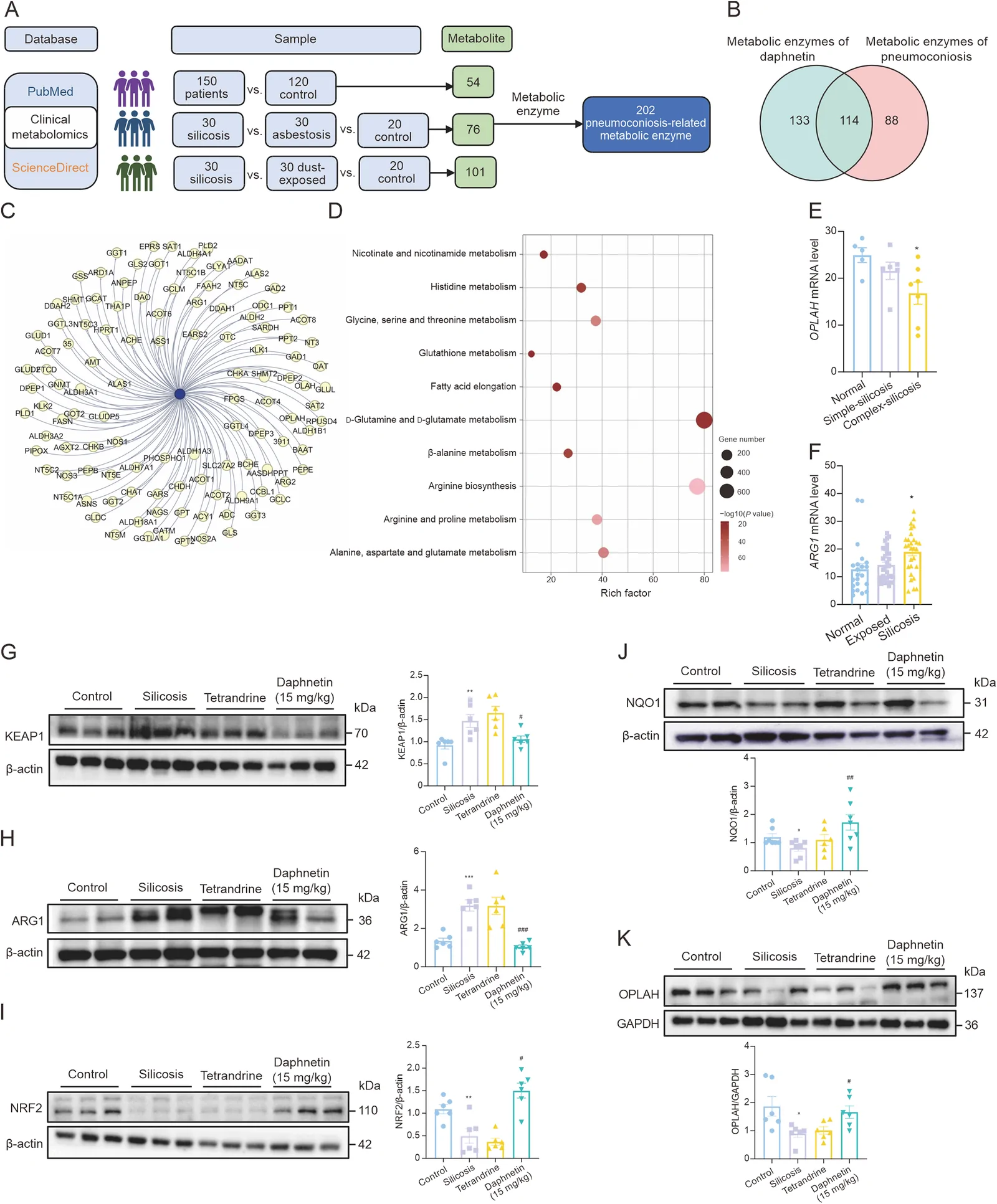
Construction, analysis and validation of the efficacy targets of daphnetin (*n* = 6). (A) Workflow for constructing the metabolite-enzyme network related to pneumoconiosis. (B) Venn diagram showing the overlap between daphnetin-regulated enzymes and pneumoconiosis-related enzymes. (C) Network of 114 metabolism-related efficacy targets of daphnetin. (D) Kyoto Encyclopedia of Genes and Genomes (KEGG) pathway analysis of the 114 efficacy targets. (E, F) The messenger RNA (mRNA) levels of 5-oxoprolinase (*OPLAH*) (E) and arginase 1 (*ARG1*) (F) in silicosis patients based on GSE264182 and GSE165489 datasets, respectively. (G–K) Western blot and semi-quantitative analysis of Kelch-like ECH-associated protein 1 (KEAP1) (G), ARG1 (H), nuclear factor erythroid 2-related factor 2 (NRF2) (I), nicotinamide adenine dinucleotide phosphate hydrogen (NAD(P)H) quinone oxidoreductase 1 (NQO1) (J), and OPLAH (K) in rat lung tissues. ^∗^*P* < 0.05, ^∗∗^*P* < 0.05, and ^∗∗∗^*P* < 0.001 vs. control; ^#^*P* < 0.05, ^##^*P* < 0.01, and ^###^*P* < 0.001 vs. silicosis group. GAPDH: glyceraldehyde 3-phosphate dehydrogenase.

Enzymes play a crucial role in key physiological processes. Identifying disease- or drug-related enzymes through metabolomics is a powerful approach in research. To elucidate the metabolic disturbances and enzyme alterations associated with pneumoconiosis, we analyzed three independent clinical cohorts based on serum metabolomics. Specifically, cohort 1 included 150 patients with coal workers’ pneumoconiosis and 120 healthy controls [24]; cohort 2 included 30 silicosis patients, 30 asbestosis patients, and 20 controls [25]; and cohort 3 involved 30 silicosis patients, 30 dust-exposed workers, and 20 controls [26] (Fig. 5A). A total of 71 differential metabolites were identified (Table S9) after excluding duplicates. Using KEGG annotation, a metabolite-enzyme interaction network was constructed with Cytoscape, yielding 202 pneumoconiosis-related enzymes (Table S10). KEGG pathway enrichment analysis indicated that these enzymes were enriched in amino acid metabolism pathways, particularly in arginine and proline metabolism (Fig. S4A).

#### 3.4.4 Identification of daphnetin candidate efficacy targets from metabolomics

To further investigate the metabolite-enzyme network regulated by daphnetin, pseudo-targeted metabolomics was performed on lung tissues from control, silicosis, tetrandrine, and daphnetin (15 mg/kg) groups. Chromatograms of the QC sample in both electrospray ionization positive (ESI^+^) and ESI negative (ESI^–^) are shown in Figs. S4B and C. All detected metabolites showed peak area RSD% values below 30% in QC samples (Table S11). Among 116 silicosis-associated metabolites, 55 were significantly reversed by daphnetin (*P* < 0.05, FC ≥ 1.2 or FC < 0.8), as shown in Table S12. Heatmaps confirmed a clear distinction between control and silicosis groups, with daphnetin treatment showing a stronger reversal effect than tetrandrine (Fig. S4D). KEGG pathway analysis revealed that daphnetin primarily regulated the tricarboxylic acid cycle, arginine biosynthesis, and arginine and proline metabolism (Fig. S4E). Therefore, pseudo-targeted metabolomics of amino acids in lung tissues was further performed. To correct for batch effects, ComBat normalization was applied to the two metabolomic datasets, resulting in good overlap of QC samples in the PCA plot and no significant differences in the abundance of common metabolites between batches (Figs. S4F and G), confirming the reliability of the results. As shown in Fig. S4H, the corrected data indicated that daphnetin significantly reversed the levels of ornithine, 5-oxoproline, serine, citrulline, valine, glutamate, asparagine, glycine, and spermine. Based on KEGG annotations of all metabolites associated with daphnetin, 247 corresponding enzymes were identified, which were intersected with 202 pneumoconiosis-related enzymes, resulting in 114 overlapping enzymes that were defined as the metabolomics-based efficacy targets of daphnetin (Figs. 5B and C; Table S10). KEGG pathway analysis revealed these enzymes were involved in arginine and proline metabolism, and GSH metabolism (Fig. 5D). In the arginine and proline metabolism pathway, three differential metabolites (ornithine, spermine, and citrulline) and 19 related proteins (e.g., ARG1) were identified. In the GSH metabolism pathway, two metabolites (5-oxoproline and glutamate) and seven related proteins (e.g., OPLAH) were detected. Notably, ornithine and 5-oxoproline showed significant increases in the silicosis group and were significantly decreased after daphnetin treatment (Fig. S4H). Ornithine is produced from arginine via ARG1, and OPLAH converts 5-oxoproline to glutamate, a key step in GSH synthesis. Transcriptomic analysis revealed that *OPLAH* was significantly downregulated in the BALF of silicosis patients (GSE264182) (Fig. 5E), and *ARG1* was elevated in peripheral blood from dust-exposed workers and silicosis patients (GSE165489) (Fig. 5F). These findings suggest that ARG1 and OPLAH may serve as key efficacy targets mediating daphnetin's protective effects in silicosis.

#### 3.4.5 Integration and validation of efficacy targets of daphnetin

Based on the multi-layered target identification strategy, a total of 142 efficacy targets of daphnetin were identified, including 29 transcriptomics-derived targets and 114 metabolomics-derived targets (Fig. S5A and B), indicating that metabolomics is a valuable complement to target discovery. Moreover, transcriptomic analysis suggested that the KEAP1/NRF2/NQO1 signaling pathway may be closely associated with the anti-silicosis effects of daphnetin, while metabolomic evidence highlighted ARG1 and OPLAH as key efficacy targets. Thus, western blotting was performed to determine the expression levels of these proteins in rat lung tissues. As shown in Figs. 5G–K, silica exposure significantly upregulated KEAP1 (Fig. 5G) and ARG1 (Fig. 5H), while downregulating NRF2 (Fig. 5I), NQO1 (Fig. 5J), and OPLAH (Fig. 5K). Interestingly, all these changes could be reversed after treatment with daphnetin. These results further support the notion that these proteins may be key efficacy targets of daphnetin against silicosis, and thus warrant further investigation.

#### 3.4.6 Identification of direct targets of daphnetin via Tissue-TPP

**Fig. 6:**
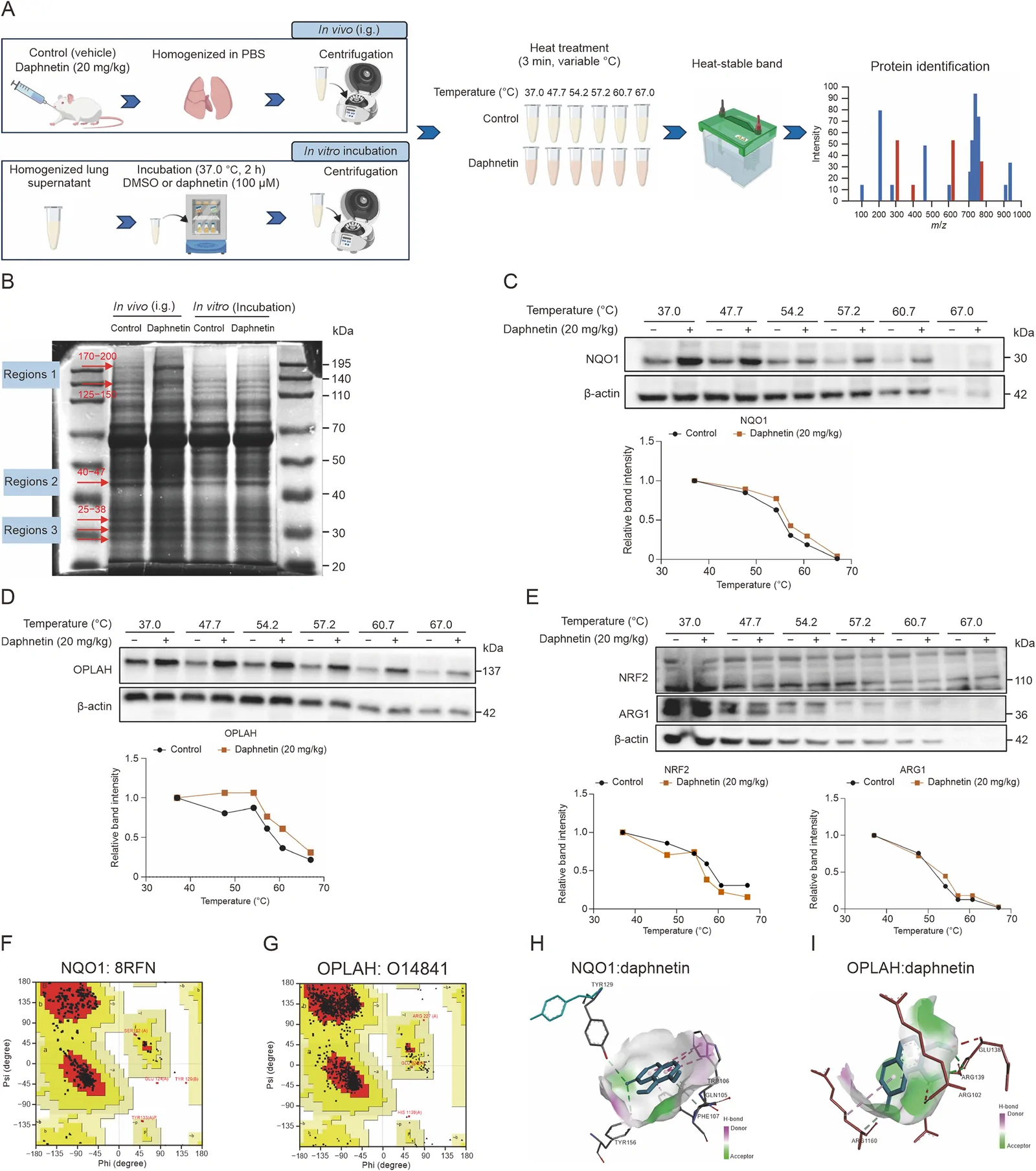
Screening and validation of direct binding targets of daphnetin. (A) Schematic of the direct target screening workflow using tissue-based thermal proteome profiling (Tissue-TPP). (B) Thermal stability profiles of lung tissue homogenates at 57.2 °C following oral or *in vitro* daphnetin treatment. (C, D) *In vivo* cellular thermal shift assay (CETSA) validating direct binding, showing immunoblots and quantified melting curves for nicotinamide adenine dinucleotide phosphate hydrogen (NAD(P)H) quinone oxidoreductase 1 (NQO1) (C) and 5-oxoprolinase (OPLAH) (D) after oral daphnetin administration. (E) CETSA analysis of nuclear factor erythroid 2-related factor 2 (NRF2) and arginase 1 (ARG1) in lung tissue shows no thermal shift upon daphnetin treatment. (F, G) Ramachandran plots evaluating the structural quality of the NQO1 (F) and OPLAH (G) models used for docking. (H, I) Predicted binding modes of daphnetin with NQO1 (H) and OPLAH (I) from molecular docking. PBS: phosphate-buffered saline; DMSO: dimethyl sulfoxide.

To further identify whether any of the five key efficacy targets are direct binding targets of daphnetin, a simplified Tissue-TPP approach was employed, as illustrated in Fig. 6A. Lung tissues, either collected from rats after oral administration of daphnetin or incubated with daphnetin *in vitro*, were subjected to a temperature gradient, followed by SDS-PAGE analysis and Coomassie blue staining. Three gel regions that exhibited altered thermal stability in both *in vivo* and *in vitro* samples were excised for LC-MS/MS analysis (Figs. 6B and S5C). A total of 564, 468, and 679 proteins were identified from regions 1, 2, and 3, respectively. Following filtration based on protein identification scores (score >100), 199 candidate binding proteins were identified (Table S13). Cross-referencing these proteins with the five key efficacy targets of daphnetin revealed NQO1 and OPLAH as the direct binding targets, while KEAP1, NRF2, and ARG1 were identified as indirect targets.

To validate the direct targets of daphnetin, CETSA experiments were performed on representative proteins. As shown in Fig. 6C, oral administration of daphnetin significantly enhanced the thermal stability of NQO1, with a *T*_m_ value of 57.27 °C for the daphnetin group, compared to 55.54 °C for the control group (Fig. S6A), indicating a substantial increase in protein stability following daphnetin treatment. A similar effect was observed in *ex vivo*-incubated lung tissues (Fig. S6B), confirming enhanced stability of NQO1 following daphnetin treatment. Similarly, OPLAH exhibited consistent thermal stabilization both *in vivo* (Figs. 6D and S6C) and *in vitro* (Fig. S6D), with a *T*_m_ value of 61.72 °C for the daphnetin group, compared to 57.95 °C for the control group supporting its direct interaction with daphnetin. In contrast, the indirect targets NRF2 and ARG1 (Figs. 6E and S6E–H) revealed no significant changes in thermal stability under either *in vivo* or *in vitro* conditions in the CETSA analysis. These results suggest that daphnetin may exert anti-fibrotic effects through multiple pathways and targets, while its interaction with NQO1 and OPLAH requires further investigation.

#### 3.4.7 Binding interaction between daphnetin and NQO1/OPLAH proteins

To elucidate the binding mechanism, molecular docking was employed to predict potential interaction sites and binding affinities. The docking analysis was performed using the crystal structure of NQO1 (PDB ID: 8RFN) and the predicted structure of OPLAH from AlphaFold DB (ID: O14841). As accurate protein structures are essential for reliable docking results, both models were evaluated using the SAVES 6.0 platform, yielding high quality scores of 97.62 for NQO1 and 90.75 for OPLAH. Ramachandran plot analysis further confirmed their structural integrity, with 99.6% and 93.5% of residues located in allowed regions, respectively (Figs. 6F and G). Docking results showed that daphnetin can directly form hydrogen bonds with residues TYR129 and TYR156 of NQO1 (binding energy: −7.6 kcal/mol) (Figs. 6H and S7A). Additionally, daphnetin forms hydrogen bonds with residue GLU138 and a carbon-hydrogen bond with ARG1160, and a PI-AlKyl bond with ARG139 of OPLAH (binding energy: −5.8 kcal/mol) (Figs. 6I and Fig. S7B). To assess the reliability of the binding energies, we performed molecular docking of NQO1 with its classical inhibitor, dicoumarol, which yielded a binding energy of −7.2 kcal/mol (Figs. S7C and D), similar to that of the NQO1-daphnetin complex. For the OPLAH-daphnetin complex, we performed virtual alanine scanning analysis on the key bond positions. The results showed that when ARG139 was mutated to alanine (ARG139 > ALA), the mutation energy was 1.52. When GLU138 > ALA, ARG139 > ALA, and ARG1160 > ALA were simultaneously mutated, the mutation energy increased to 1.63, suggesting that these mutations reduce binding affinity and weaken interactions, with ARG139 potentially serving as a key site in the OPLAH-daphnetin complex (Fig. S7E). These findings indicate that daphnetin can stably bind to both NQO1 and OPLAH.

To characterize the binding mechanisms between daphnetin and its targets, 100 ns MD simulations were performed for the NQO1-daphnetin and OPLAH-daphnetin complexes. Structural stability and interaction dynamics were evaluated using root mean square deviation (RMSD), root mean square fluctuation (RMSF), hydrogen bonds, radius of gyration (Rg), and solvent-accessible surface area (SASA). RMSD analysis showed that both complexes stabilized after approximately 20 ns for NQO1-daphnetin (σ = 2 Å) and approximately 30 ns for OPLAH-daphnetin (σ = 3 Å) (Figs. S7F and G), indicating stable conformations throughout the simulation. RMSF analysis revealed that protein residues within the binding pockets of both complexes remained stable (<4 Å) (Figs. S7H and I), with increased flexibility observed only at the N- and C-terminal regions in the OPLAH-daphnetin complex. Hydrogen bond analysis showed that the daphnetin-NQO1 complex maintained 2–3 hydrogen bonds initially, which decreased to 1–2 after 75 ns (Fig. S7J), while the daphnetin-OPLAH complex formed 2–3 stable hydrogen bonds at the beginning and increased to 5–6 bonds between 40 and 50 ns (Fig. S7K), indicating enhanced interaction strength. Both complexes exhibited stable Rg and SASA values, with daphnetin-NQO1 ranging from 23.1 to 23.4 Å (Fig. S7L) and daphnetin-OPLAH from 31.6 to 32.0 Å for Rg (Fig. S7M), and SASA values ranging from 240 to 250 nm^2^ for daphnetin-NQO1 (Fig. S7N) and 485 to 505 nm^2^ for daphnetin-OPLAH (Fig. S7O). Together, these findings provide a detailed molecular basis for the stable and high-affinity binding of daphnetin to both NQO1 and OPLAH.

### 3.5 Daphnetin could directly regulate NQO1 expression

**Fig. 7:**
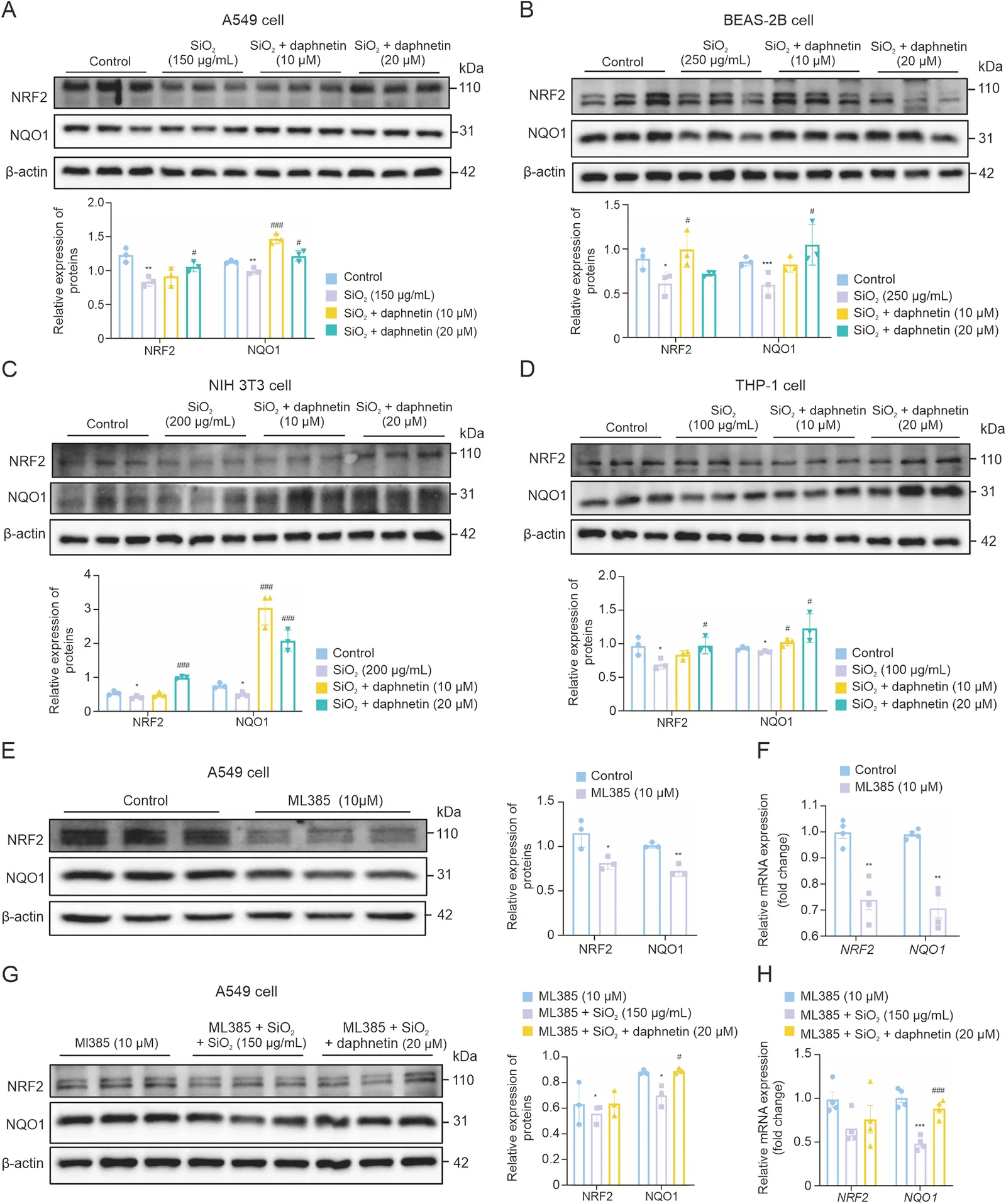
Daphnetin directly regulated the expression of nicotinamide adenine dinucleotide phosphate hydrogen (NAD(P)H) quinone oxidoreductase 1 (NQO1) independent of nuclear factor erythroid 2-related factor 2 (NRF2) (*n* = 3). (A–D) The effect of daphnetin on the expression levels of NRF2 and NQO1 in A549 (A), BEAS-2B (B), NIH 3T3 (C), and THP-1 (D) cells exposed to silica. (E, F) The effect of ML385 on the protein (E) and messenger RNA (mRNA) (F) levels of NRF2 and NQO1 in A549 cells. (G, H) The effect of ML385 combined with daphnetin on the protein (G) and mRNA (H) levels of NRF2 and NQO1 in A549 cells exposed to silica (150 μg/mL). ^∗^*P* < 0.05, ^∗∗^*P* < 0.01, and ^∗∗∗^*P* < 0.001 vs. control group; ^#^*P* < 0.05 and ^###^*P* < 0.001 vs. model group.

NRF2 is a pivotal redox-sensitive transcription factor that, under oxidative stress, translocates to the nucleus and leads to the expression of various antioxidant genes to alleviate oxidative damage, such as NQO1. Our results identified NQO1 as a direct target of daphnetin, whereas NRF2 functions as an indirect target. To further elucidate the relationship between daphnetin, NQO1, and NRF2, we first investigated the effect of daphnetin on the expression of NQO1 and NRF2 at the cellular levels. As shown in Figs. 7A–D, daphnetin treatment significantly reversed the reduction in NQO1 and NRF2 expression caused by silica exposure in epithelial cells (A549 and BEAS-2B), NIH 3T3 fibroblasts and THP-1 macrophages, further confirming that both NQO1 and NRF2 are important targets of daphnetin. Subsequently, to determine whether daphnetin's regulation of NQO1 depends on NRF2, we performed experiments in A549 cells using the NRF2 inhibitor ML385. The results showed that ML385 supplementation suppressed NRF2 expression at both the protein (Fig. 7E) and mRNA levels (Fig. 7F), which was accompanied by a decrease in NQO1 expression, confirming that NQO1 is partially regulated by NRF2. Next, we investigated whether ML385 could inhibit daphnetin's regulation of NRF2. The results revealed that after ML385 treatment, silica exposure further decreased NRF2 levels at both the protein (Fig. 7G) and mRNA levels (Fig. 7H), while daphnetin was unable to restore NRF2 expression. However, despite the absence of significant recovery of NRF2, daphnetin treatment notably increased the expression of NQO1. These findings further suggest that NQO1 is a direct target of daphnetin.

### 3.6 OPLAH-mediated antifibrotic effects of daphnetin

**Fig. 8:**
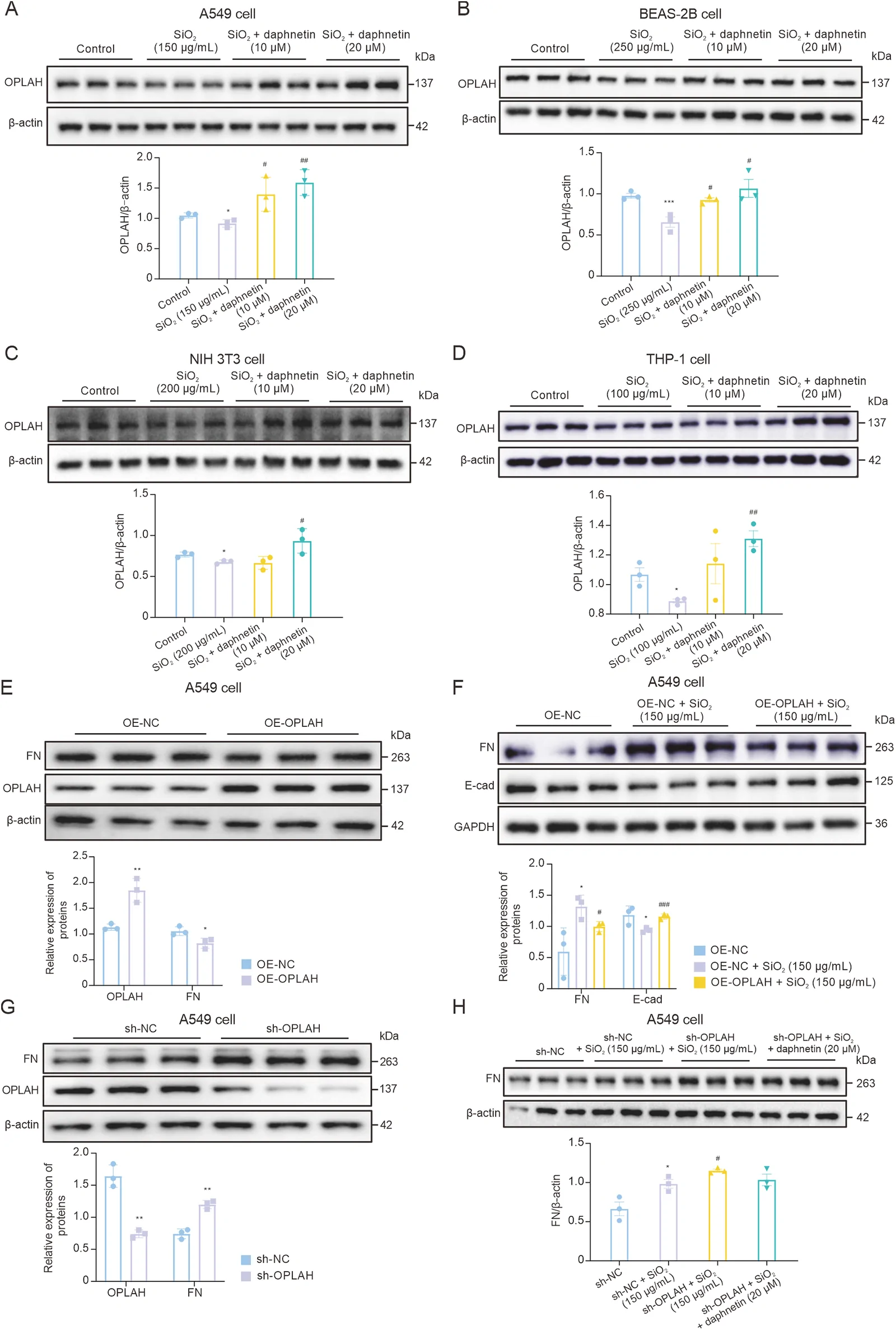
5-Oxoprolinase (OPLAH)-mediated antifibrotic effects of daphnetin (*n* = 3). (A–D) The effect of daphnetin on the expression levels of OPLAH in A549 (A), BEAS-2B (B), NIH 3T3 (C), and THP-1 (D) cells exposed to silica. (E) Expression levels of OPLAH and fibronectin (FN) in A549 cells 48 h after transfection with the OPLAH overexpression (OE) plasmid. (F) The effect of OPLAH OE on the expression of FN and E-cadherin (E-cad) in A549 cells exposed to silica. (G) Expression levels of OPLAH and FN in A549 cells 48 h after transfection with the OPLAH knockdown plasmid. (H) The effect of daphnetin on FN expression in A549 cells with OPLAH knockdown exposed to silica (150 μg/mL). ^∗^*P* < 0.05, ^∗∗^*P* < 0.01, and ^∗∗∗^*P* < 0.001 vs. control group; ^#^*P* < 0.05, ^##^*P* < 0.01, and ^###^*P* < 0.001 vs. model group. GAPDH: glyceraldehyde 3-phosphate dehydrogenase; sh-NC: short hairpin RNA (shRNA) negative control.

In addition to NQO1, OPLAH was identified in this study as a previously unrecognized direct target of daphnetin. We also evaluated the effect of daphnetin on the expression of OPLAH at the cellular levels. Consistent with the effect on NQO1, treatment with daphnetin significantly reversed the decrease in OPLAH expression caused by silica exposure in A549 cells (Fig. 8A), BEAS-2B cells (Fig. 8B), NIH 3T3 fibroblasts (Fig. 8C) and THP-1 macrophages (Fig. 8D). To investigate the role of OPLAH in pulmonary fibrosis, we first overexpressed OPLAH in A549 alveolar epithelial cells. This OE directly suppressed FN expression (Fig. 8E). This anti-fibrotic effect was further confirmed in a silica-induced model, where OPLAH OE attenuated the silica-induced FN upregulation and E-cad downregulation (Fig. 8F), indicating a critical role for OPLAH in modulating fibrotic responses. To determine the necessity of OPLAH for daphnetin's anti-fibrotic effects, we knocked down OPLAH expression, which significantly increased basal FN levels (Fig. 8G). Moreover, in these knockdown cells, the inhibitory effect of 20 μM daphnetin on silica-induced FN expression was partly abolished (Fig. 8H). Furthermore, OPLAH knockdown exacerbated the fibrotic response to silica. These results confirmed that OPLAH partly mediated the antifibrotic effect of daphnetin, and that OPLAH may also serve as a potential therapeutic target for pulmonary fibrosis.

### 3.7 Daphnetin alleviates oxidative stress by modulating NQO1 and OPLAH expression

**Fig. 9:**
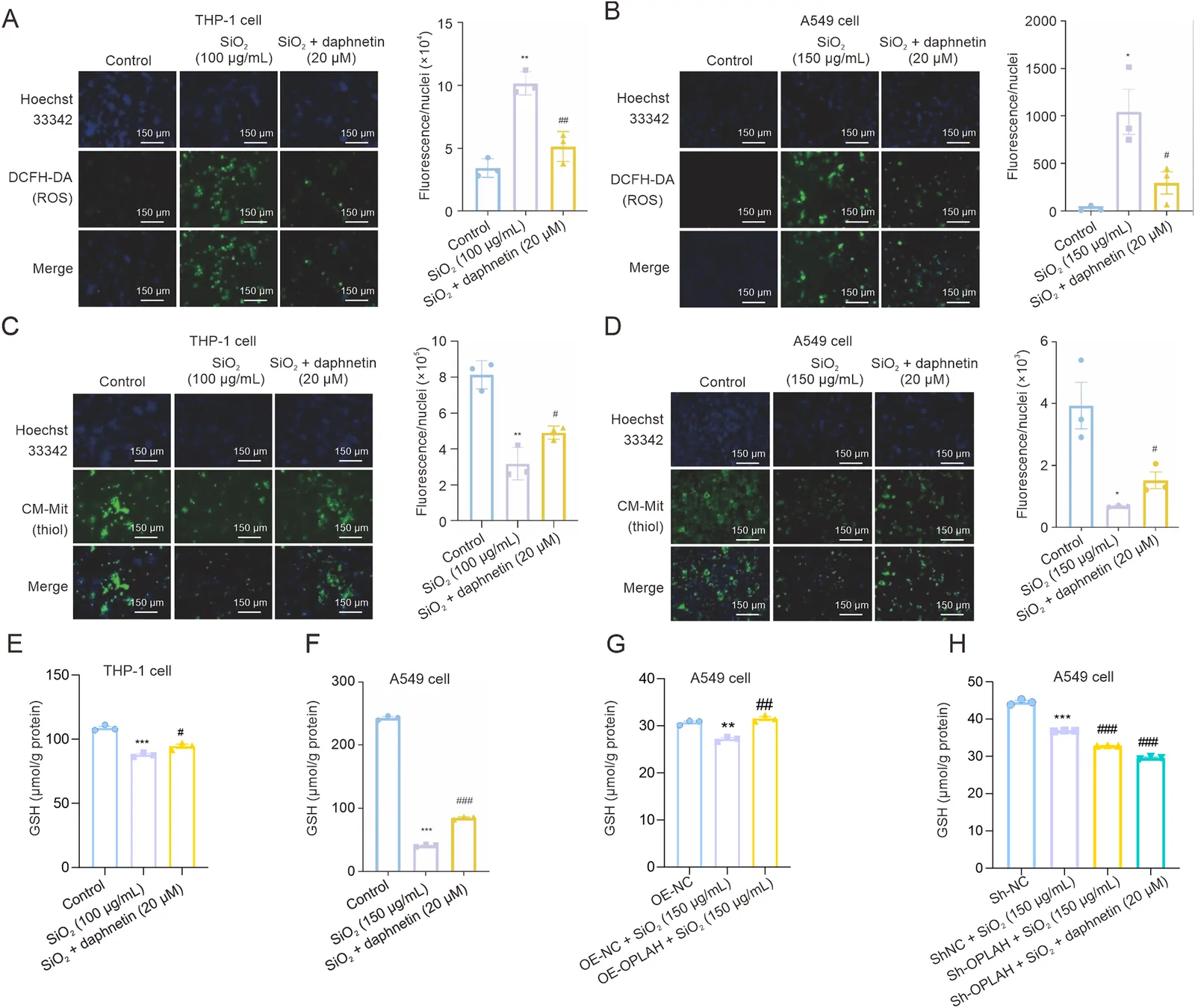
Daphnetin attenuates silica-induced oxidative stress by preserving intracellular thiol levels (*n* = 3). (A, B) Representative fluorescence images and quantification of reactive oxygen species (ROS) in THP-1 (A) and A549 (B) cells treated with silica for 24 h in the presence or absence of daphnetin. (C, D) Representative fluorescence images and quantification of intracellular thiol levels in THP-1 (C) and A549 (D) cells under the same treatment conditions. (E, F) Glutathione (GSH) levels in THP-1 (E) and A549 (F) cells after treatment with silica in the presence or absence of daphnetin. (G) GSH levels in A549 cells with 5-oxoprolinase (OPLAH) overexpression (OE) following treatment with silica (150 μg/mL). (H) GSH levels in A549 cells with OPLAH knockdown following treatment with silica (150 μg/mL) in the presence or absence of daphnetin. ^∗^*P* < 0.05, ^∗∗^*P* < 0.01, and ^∗∗∗^*P* < 0.001 vs. control; ^#^*P* < 0.05, ^##^*P* < 0.01, and ^###^*P* < 0.001 vs. model group. DCFH-DA: 2′,7′-dichlorodihydrofluorescein diacetate; NC: negative control; sh-NC: short hairpin RNA (shRNA) NC.

Both NQO1 and OPLAH are key regulators of redox homeostasis, suggesting that daphnetin may exert its antioxidant effects by modulating them. Previous studies have demonstrated that silica exposure induces oxidative stress in macrophages, epithelial cells, and fibroblasts. Among these, macrophages can produce large amounts of ROS after phagocytosing silica particles [27]. Therefore, we first measured ROS levels in THP-1 and A549 cells. As shown in Figs. 9A and B, silica exposure significantly increased ROS levels after 24 h in both cell types, while daphnetin markedly decreased ROS accumulation.

Given that thiols act as major antioxidants by directly eliminating ROS [28], intracellular thiol levels were evaluated using the CM-Mit probe. As illustrated in Figs. 9C and D, daphnetin significantly increased thiol levels in THP-1 and A549 cells, which were otherwise reduced by silica exposure. Since GSH is a crucial thiol and is closely associated with OPLAH-mediated glutamate production, we also examined GSH levels in THP-1 (Fig. 9E) and A549 cells (Fig. 9F). Similar results were observed with the CM-Mit probe assay, indicating that daphnetin effectively reversed the decrease in GSH levels caused by silica exposure. To further investigate whether daphnetin alleviates oxidative stress by upregulating OPLAH expression and enhancing GSH synthesis, we first measured GSH levels in OPLAH-overexpressing A549 cells following silica exposure (Fig. 9G). The results showed that OPLAH OE significantly attenuated the silica-induced decrease in intracellular GSH. We next quantified GSH levels in OPLAH-knockdown A549 cells (Fig. 9H). Notably, OPLAH knockdown further reduced GSH levels compared with silica treatment alone, and daphnetin failed to restore GSH under OPLAH-deficient conditions. These findings suggest that daphnetin alleviates oxidative stress, at least in part, by enhancing the expression of NQO1 and OPLAH, contributing to its antifibrotic effects.

## 4 Discussion

Silicosis, a serious occupational lung disease, is characterized by progressive pulmonary parenchymal damage, chronic inflammation, and fibrosis. Although its pathogenesis has been extensively investigated using multi-omics approaches, effective pharmacological treatments remain unavailable. Daphnetin, a natural coumarin derivative, is a commercially available drug in China for the treatment of thromboangiitis obliterans and other occlusive vascular diseases [29]. This study systematically investigated the efficacy of daphnetin using a silica-induced rat model with tetrandrine as a positive control. Remarkably, daphnetin at a dose of 15 mg/kg exhibited superior efficacy to tetrandrine (30 mg/kg) in improving the lung index and suppressing TGF-β1 secretion. Furthermore, 6-min walk tests have demonstrated reduced exercise tolerance in silicosis patients [30]. Here, we show for the first time that daphnetin significantly improves motor performance in silicosis rats, whereas tetrandrine shows a weak effect, highlighting daphnetin's unique therapeutic potential in restoring exercise capacity. In addition, daphnetin was effectively distributed to lung tissue and directly inhibited EMT and fibroblast activation, thereby mitigating fibrotic remodeling. Meanwhile, previous studies have also provided further insight into the therapeutic potential of daphnetin in pulmonary diseases, such as attenuating chronic obstructive pulmonary disease [31], reducing bleomycin-induced pulmonary fibrosis [32], and alleviating lipopolysaccharide-induced lung injury [33]. These findings suggest that daphnetin could be a potential therapeutic candidate for silicosis.

NPs typically exert pharmacological effects by directly binding to disease-related proteins (direct targets) in organisms, or by modulating the expression or activity of indirect targets, thereby correcting disease-induced imbalances in the body [4]. Direct targets are typically identified using chemical proteomics, CETSA, and molecular docking, while indirect targets are identified using phenotype-based strategies, such as transcriptomics, proteomics, and cell painting. The availability of public databases such as GEO [8], GeneCards [10], and CMap [11] has enabled large-scale integration of omics data and facilitated systematic target discovery. In this study, we developed a multi-layered target identification strategy that proceeds stepwise from disease-associated targets to drug-responsive targets and ultimately to direct binding proteins. By integrating multi-omics data, public databases, the FRoGS target prediction model, and Tissue–TPP technology, this framework enabled stepwise refinement from indirect to direct targets. Using this approach, we identified three key efficacy targets of daphnetin (KEAP1, NRF2, and NQO1) based on transcriptomic data, and two additional key efficacy targets (ARG1 and OPLAH) based on metabolomic analysis. Among them, NQO1 and OPLAH were further validated as direct binding targets, while KEAP1, NRF2, and ARG1 were identified as indirect targets. Our study also observed that daphnetin significantly upregulated OPLAH and NQO1 expression in lung tissue and reduced 5-oxoproline accumulation. Furthermore, we found that silica exposure led to a decrease in NQO1 and OPLAH in several cells, suggesting that they may be important targets in silicosis. More importantly, daphnetin significantly restored the expression of these proteins, indicating their potential as key targets for daphnetin in mitigating silicosis.

OPLAH is a rate-limiting enzyme in the GSH recycling pathway that converts 5-oxoproline into glutamate. Its deficiency leads to elevated 5-oxoproline, impaired GSH synthesis, and increased ROS accumulation, contributing to fibrosis progression [34]. Notably, OPLAH OE significantly inhibited silica-induced EMT, while knockdown of OPLAH reduced the inhibitory effect of daphnetin on FN expression. We also confirmed that OPLAH promotes GSH synthesis and enhances thiol levels, thereby inhibiting silica-induced ROS. Interestingly, studies have shown that OPLAH knockout mice not only developed a cardiac phenotype but also exhibited increased renal fibrosis, with accumulation of 5-oxoproline in myocardial dysfunction [35]. These findings suggest that OPLAH may represent a novel target for the discovery of drugs against organ fibrosis. Meanwhile, NQO1, a downstream antioxidant enzyme regulated by NRF2, can reduce ROS levels and inhibit the TGF-β/Smad signaling pathway [36]. As a direct target of daphnetin, we found that daphnetin might directly regulate NQO1 expression, partly independent of NRF2 regulation. Moreover, OE of NQO1 significantly suppressed the secretion of pro-inflammatory cytokines, ECM accumulation, and EMT, thereby alleviating renal fibrosis [36]. These results collectively suggest that daphnetin may inhibit oxidative stress by directly targeting OPLAH and NQO1, thus mitigating the progression of silicosis.

Finally, this study proposes a rapid target identification strategy for NPs and successfully identifies both direct and indirect targets of daphnetin in alleviating pneumoconiosis. However, target discovery for NPs remains challenging. Both direct chemical proteomics, which modifies small molecules to “fish” for targets, and label-free TPP have limitations. For example, chemical modifications in direct chemical proteomics can alter the activity of NPs and cause non-specific binding, affecting the accuracy of target identification [37]. Therefore, excluding non-specific binding and focusing on key targets is crucial. To address this, we integrated transcriptomics and metabolomics data to identify targets at the phenotypic level [3], complemented by Tissue-TPP for refined direct target identification. This narrowed the validation scope and accelerated target discovery. However, further exploration is needed to precisely identify disease or small molecule targets using AI in bioinformatics. Furthermore, although daphnetin shows promise for pneumoconiosis treatment, its long-term use requires further safety studies.

## 5 Conclusion

In summary, this study proposed a multi-layered strategy for small-molecule target identification by integrating transcriptomics, metabolomics, the FRoGS target prediction model, public databases, and Tissue-TPP technology. Using daphnetin as a case study, we found that its therapeutic effects against silicosis may be partially attributed to the regulation of five key targets: NQO1, OPLAH, KEAP1, NRF2, and ARG1. Among them, NQO1 and OPLAH were validated as direct binding targets, while KEAP1, NRF2, and ARG1 were identified as indirect targets. Furthermore, NQO1 and OPLAH are confirmed as important targets for silicosis, as silica exposure led to decreased expression of these proteins in epithelial cells, macrophages, and fibroblasts, which was significantly restored by daphnetin treatment. Functional studies using OPLAH OE and knockdown models showed that OPLAH partially mediates the anti-fibrotic effects of daphnetin, likely through promoting GSH synthesis, increasing intracellular thiol levels, and reducing ROS accumulation. Collectively, these findings provide mechanistic insight into the anti-fibrotic actions of daphnetin and support its potential as a promising therapeutic agent for silicosis.

## CRediT authorship contribution statement

**Ling Wang:** Writing – original draft, Software, Project administration, Investigation, Formal analysis, Data curation, Conceptualization, Validation. **Bo-Yan Ma:** Visualization, Software, Formal analysis, Methodology, Validation. **Sheng-Ping Jiang:** Visualization, Software, Data curation, Validation. **Shu-Ting Wei:** Validation, Resources, Project administration. **Xue-Mei Qin:** Methodology, Conceptualization, Project administration, Supervision, Writing – review & editing. **Zhen-Yu Li:** Writing – review & editing, Supervision, Resources, Methodology, Funding acquisition, Conceptualization, Project administration.

## Declaration of competing interest

The authors declare that they have no known competing financial interests or personal relationships that could have appeared to influence the work reported in this paper.
