# Supplementary material for: Multi-layered target identification strategy of natural products: Daphnetin targets NQO1 and OPLAH in silicosis alleviation
Source: J Pharm Anal. 2026 Apr 2;16(9):101629. doi: 10.1016/j.jpha.2026.101629 (PMC13602248; doi:10.1016/j.jpha.2026.101629)

Fig. 2A

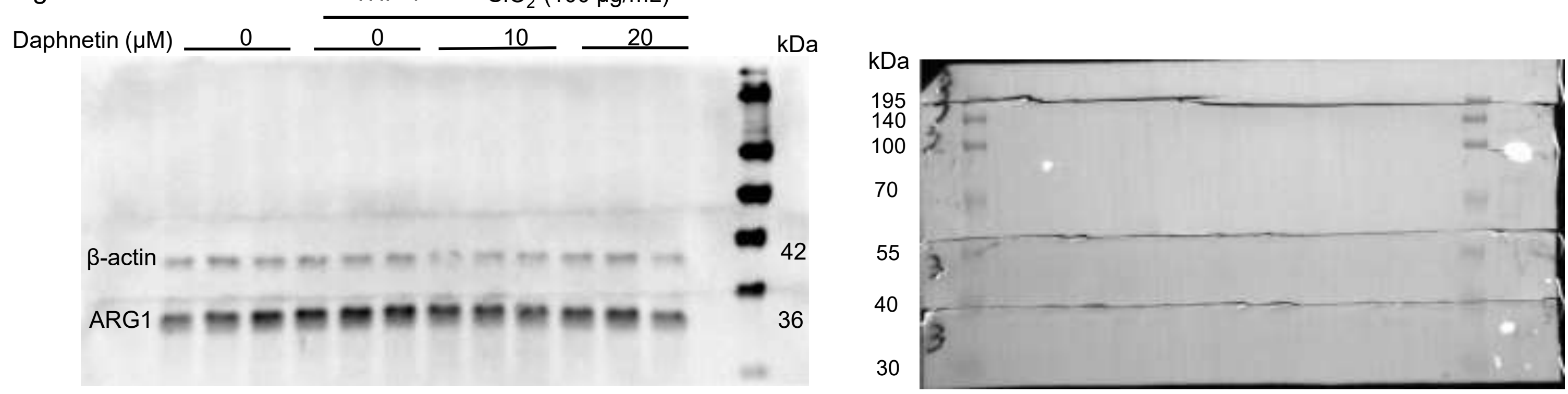

Fig. 2B

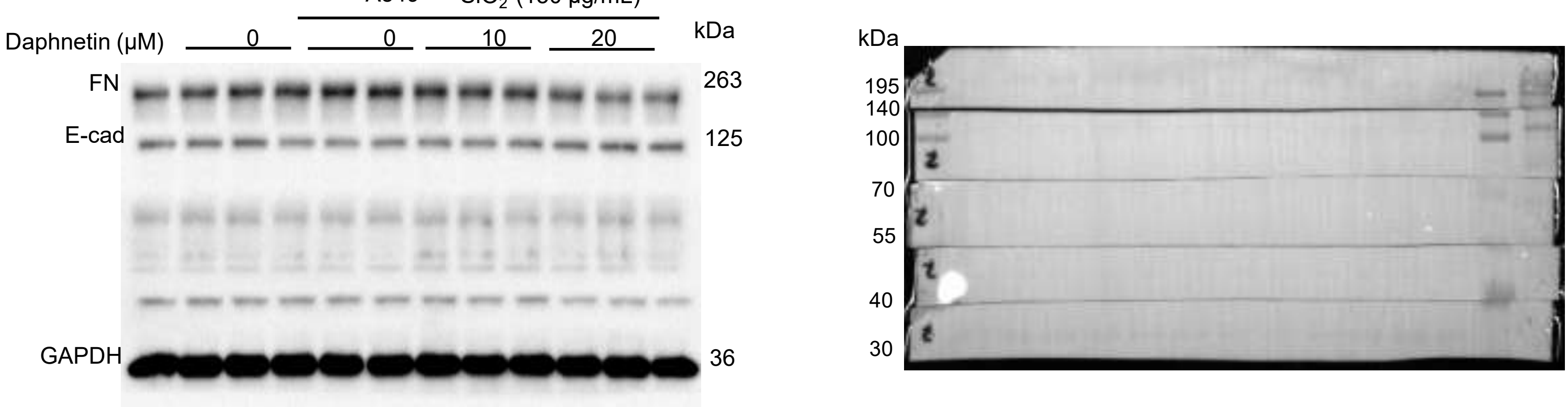

Fig. 2C

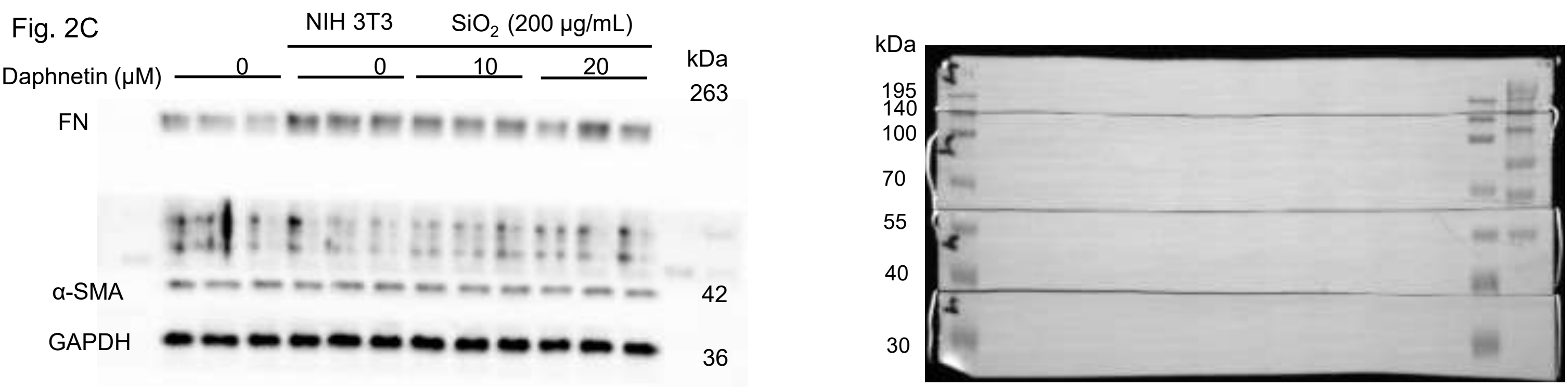

Fig. 2D

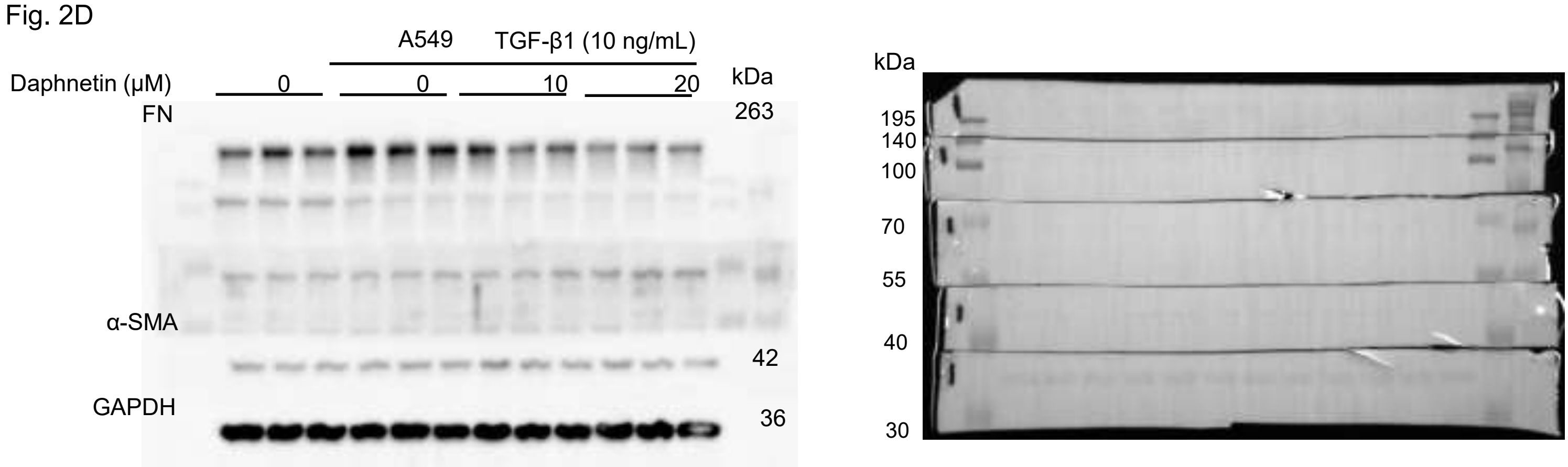

Fig. 2E

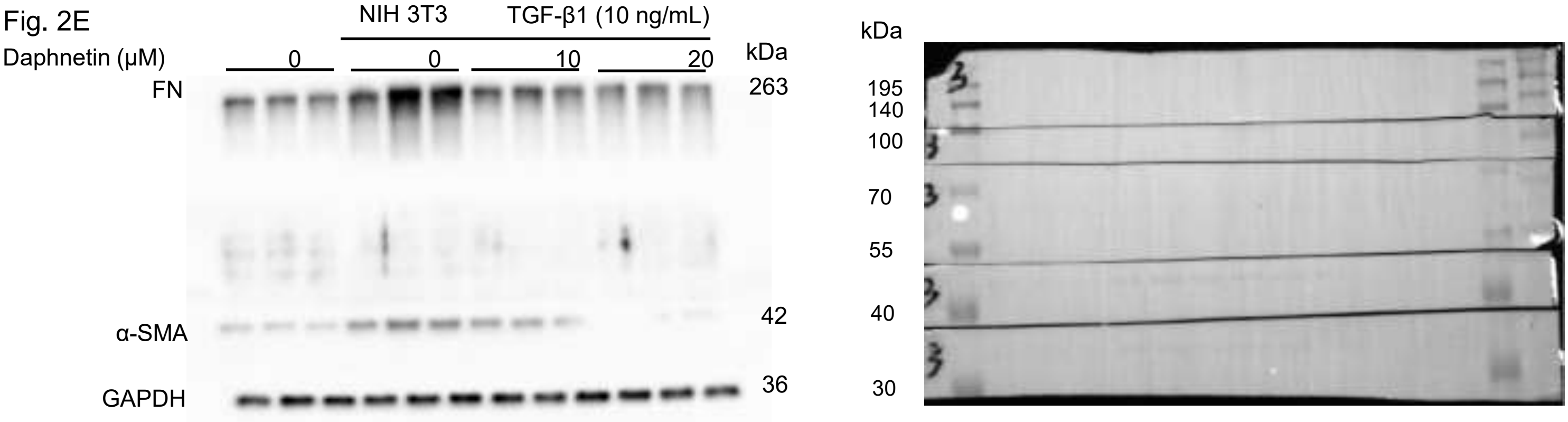

Fig. 5G

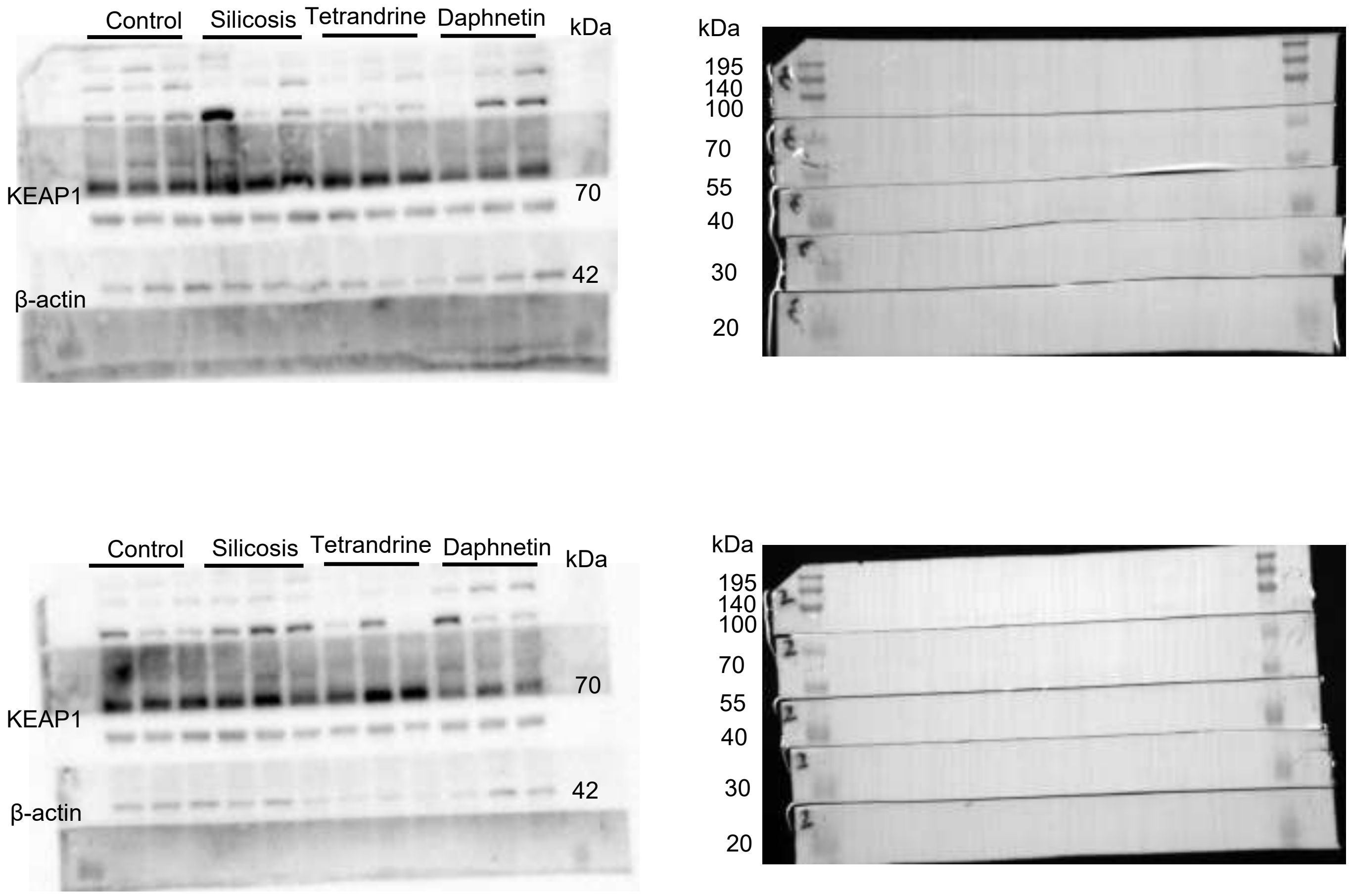

Control
Silicosis
Tetrandrine
Daphnetin
kDa
KEAP1
70
β-actin
42
kDa
195
140
100
70
55
40
30
20

Fig. 5H

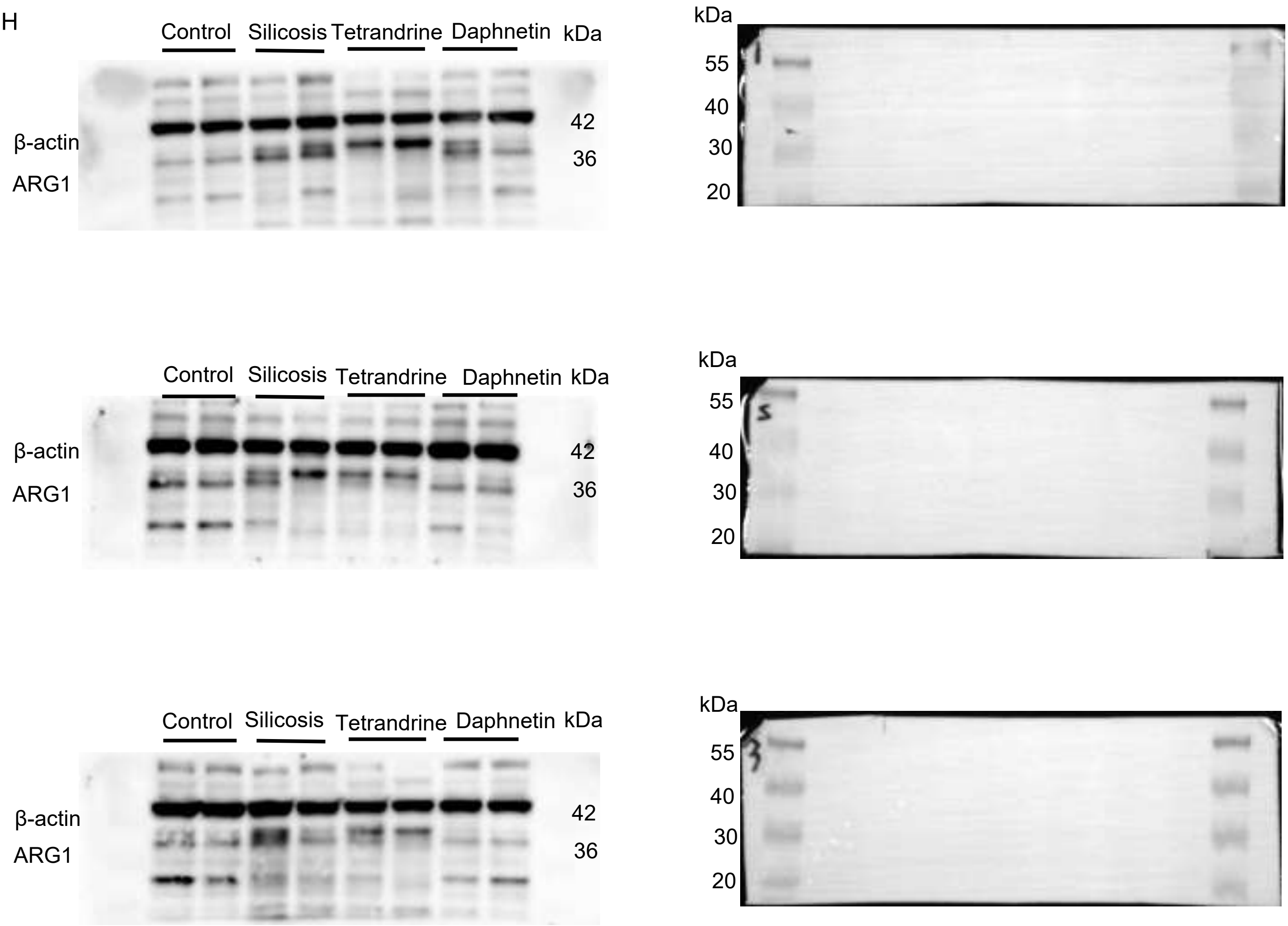

Control
Silicosis
Tetrandrine
Daphnetin
kDa
β-actin
42
ARG1
36
kDa
55
40
30
20

Fig. 5I

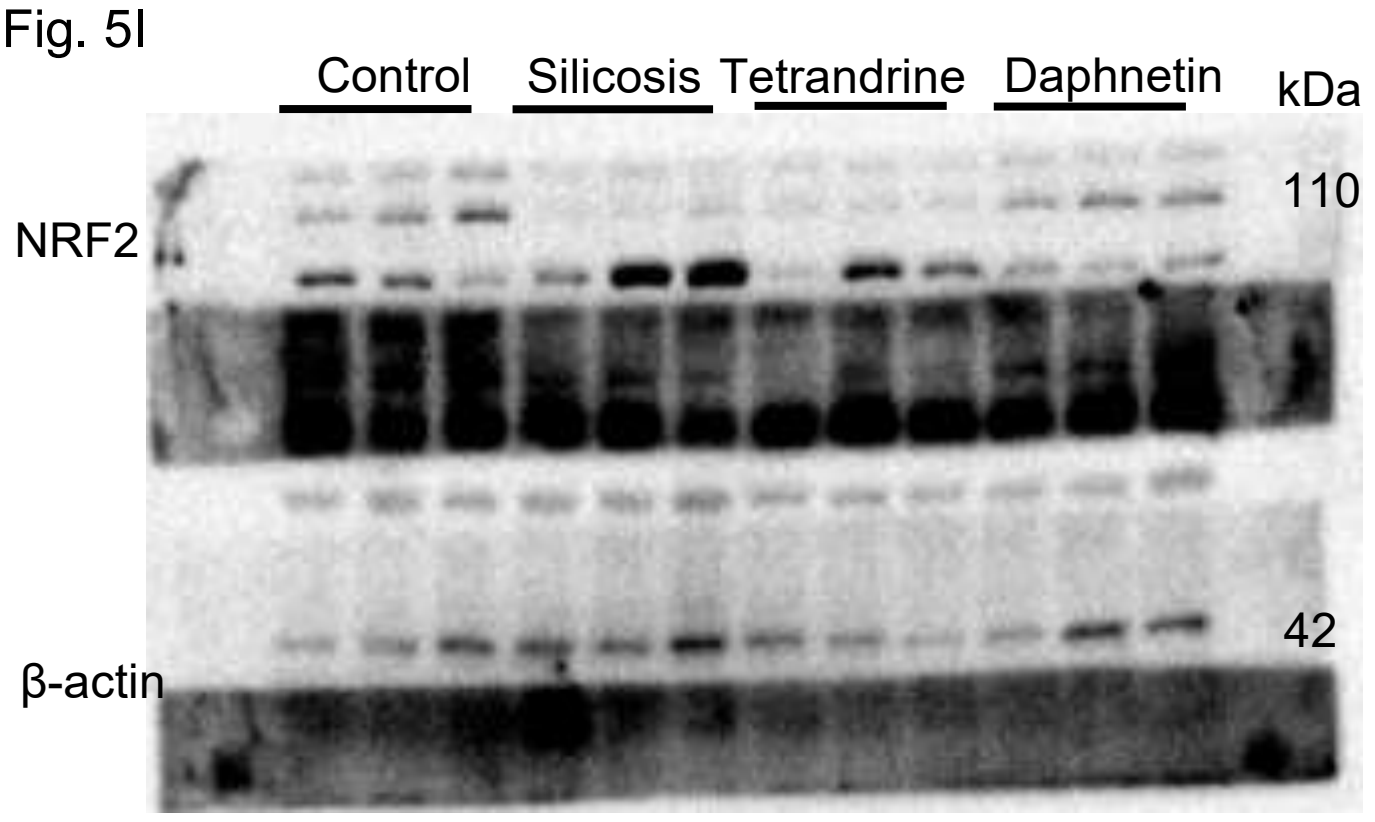
Control
Silicosis
Tetrandrine
Daphnetin
kDa
NRF2
110
β-actin
42

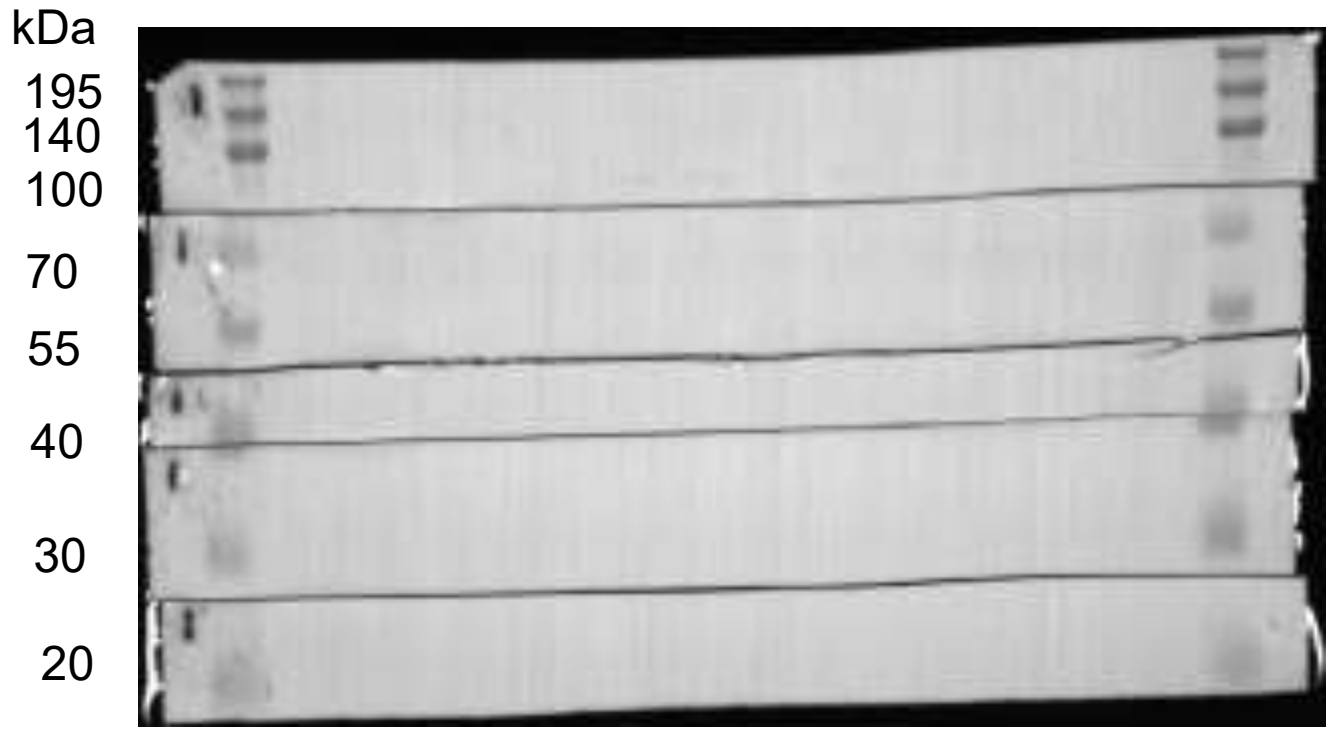
kDa
195
140
100
70
55
40
30
20

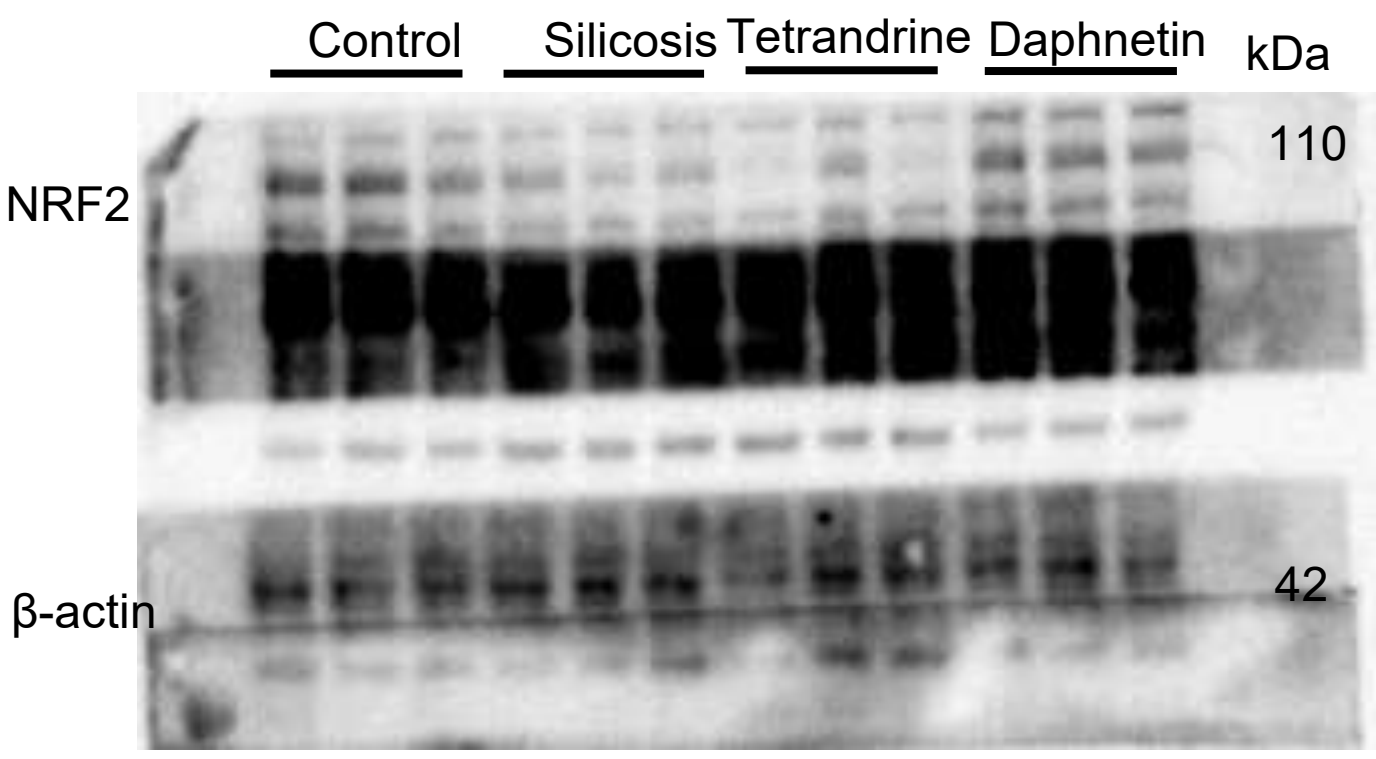
Control
Silicosis
Tetrandrine
Daphnetin
kDa
NRF2
110
β-actin
42

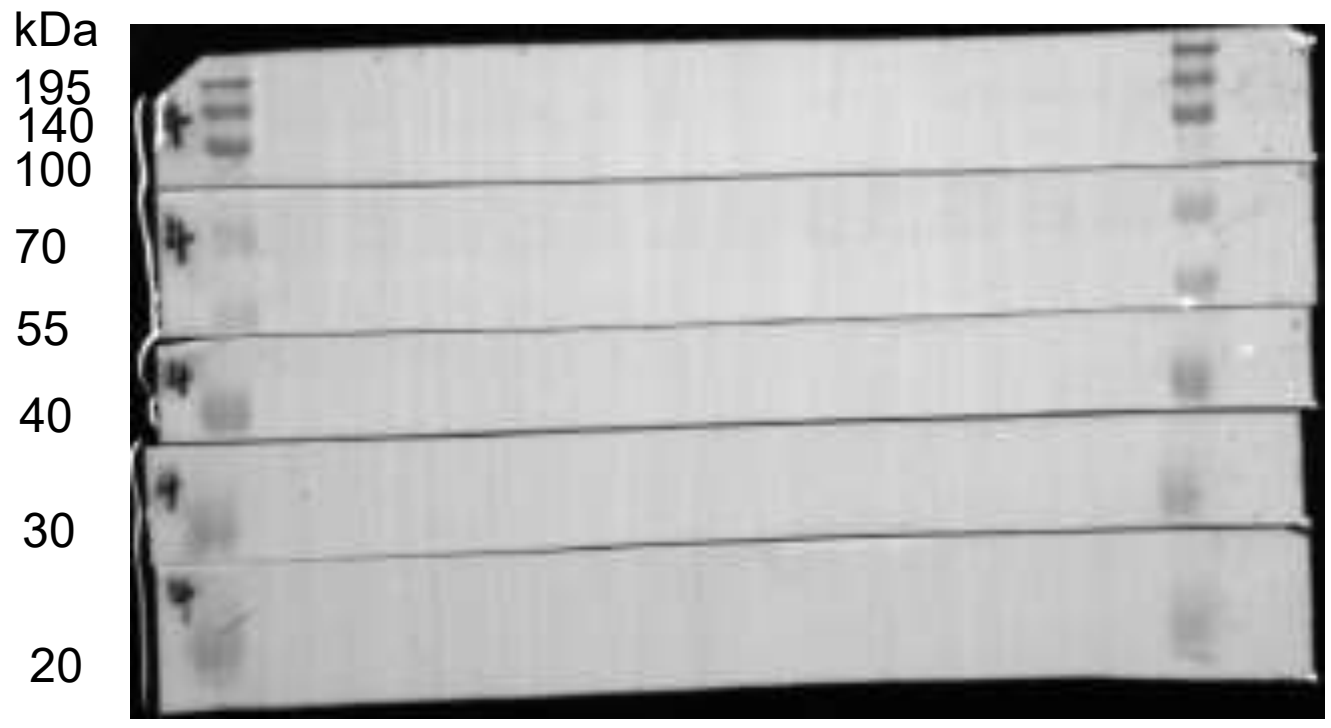
kDa
195
140
100
70
55
40
30
20

Fig. 5J

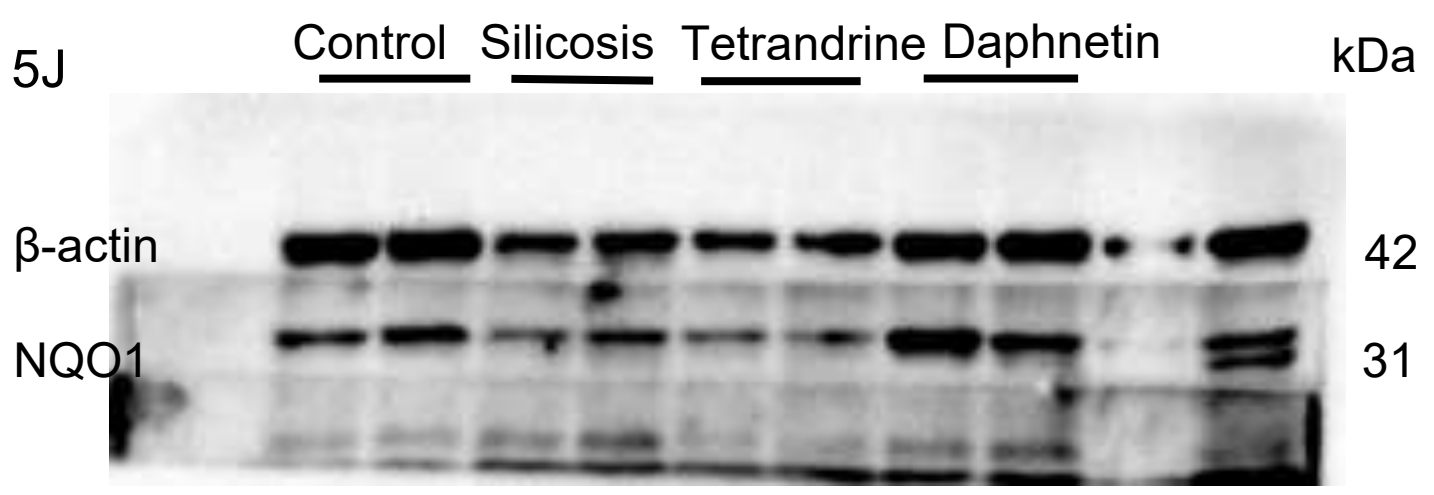
Control
Silicosis
Tetrandrine
Daphnetin
kDa
β-actin
42
NQO1
31

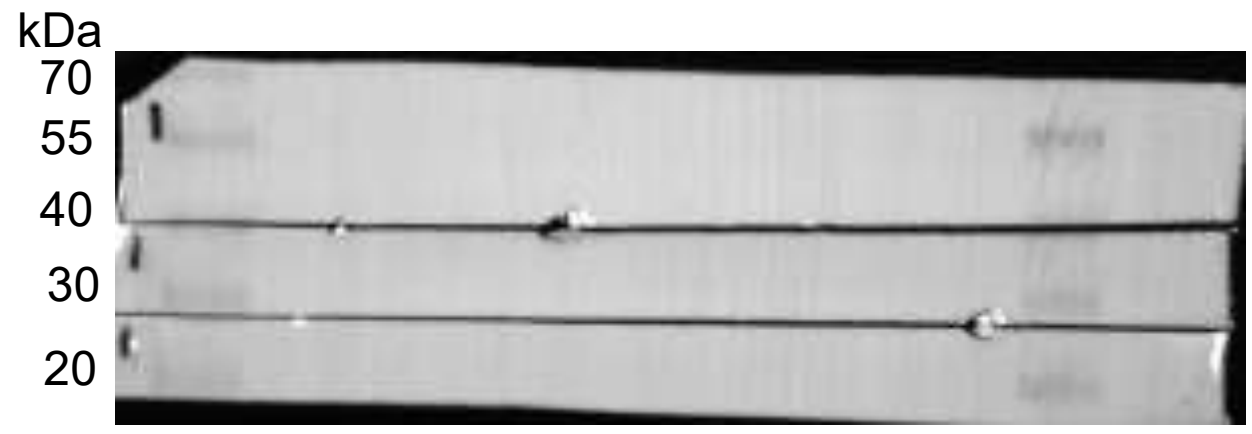
kDa
70
55
40
30
20

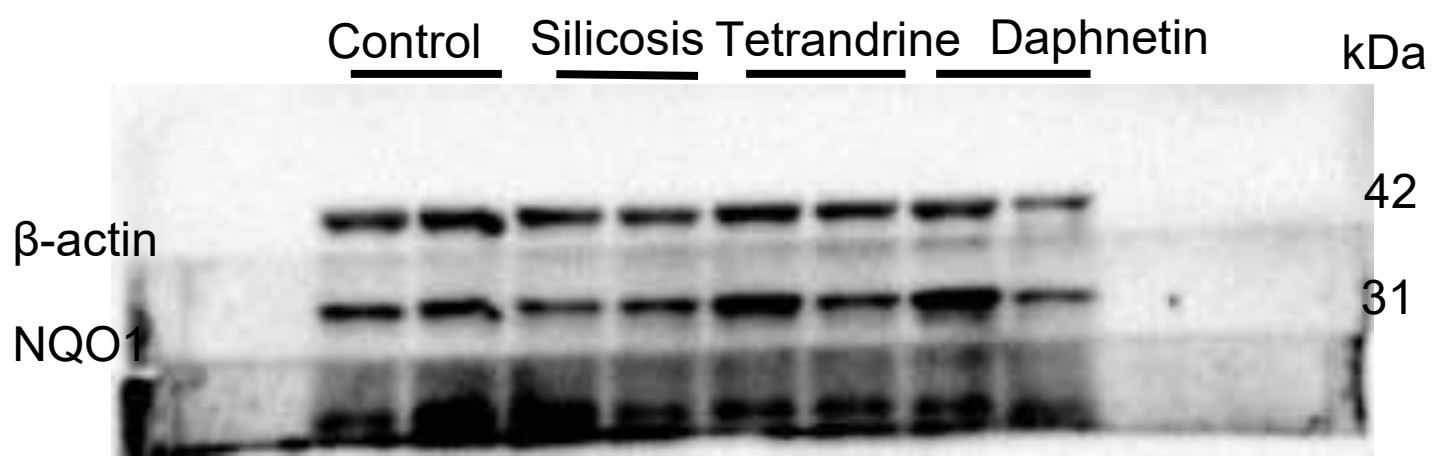
Control
Silicosis
Tetrandrine
Daphnetin
kDa
β-actin
42
NQO1
31

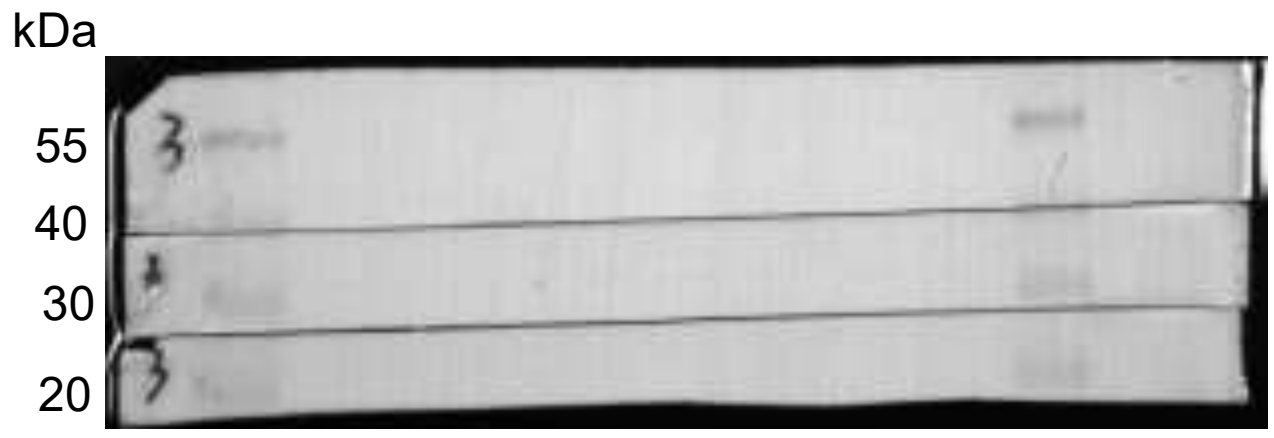
kDa
55
40
30
20

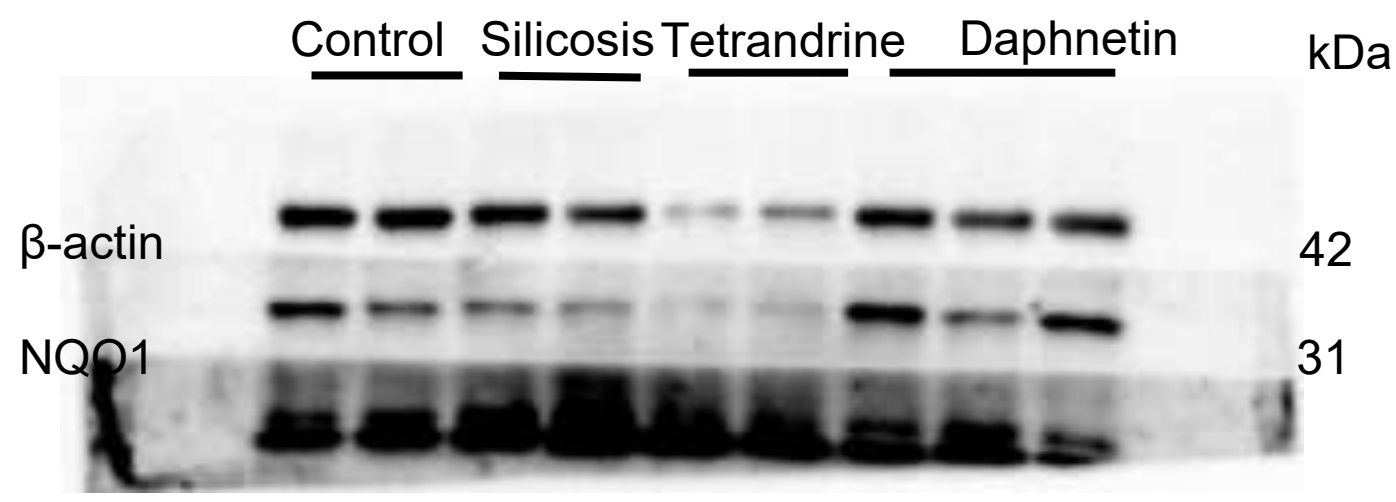
Control
Silicosis
Tetrandrine
Daphnetin
kDa
β-actin
42
NQO1
31

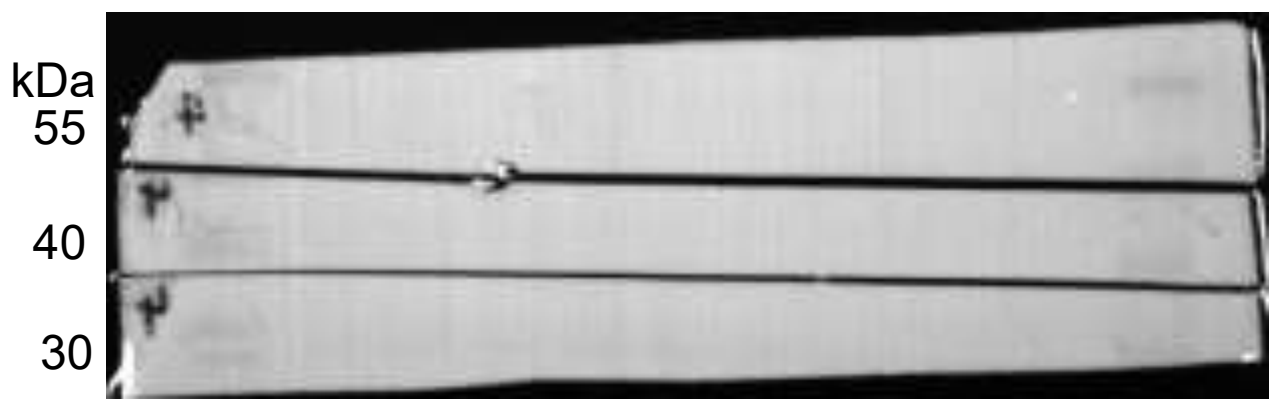
kDa
55
40
30

Fig. 5K

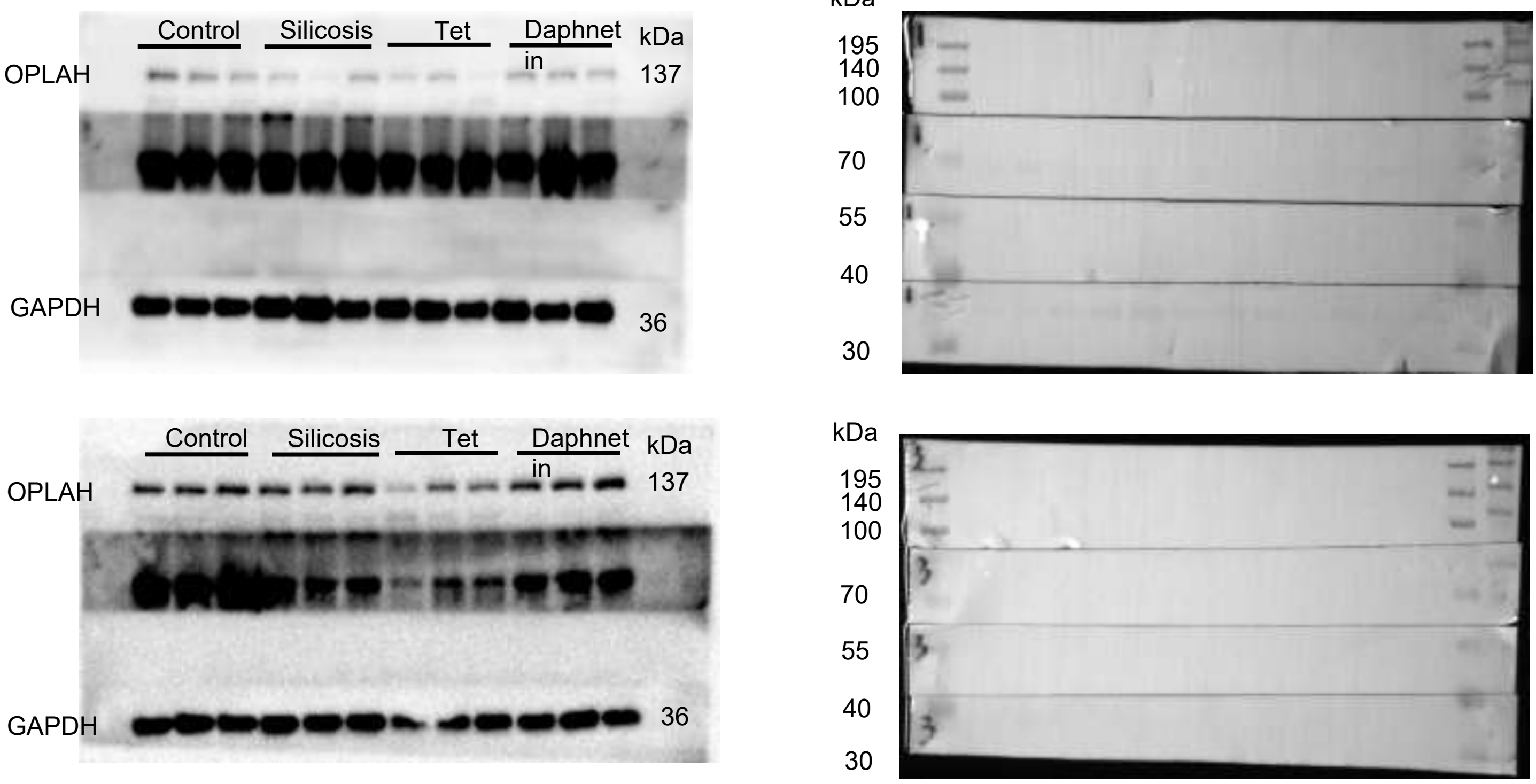

Fig. 6C

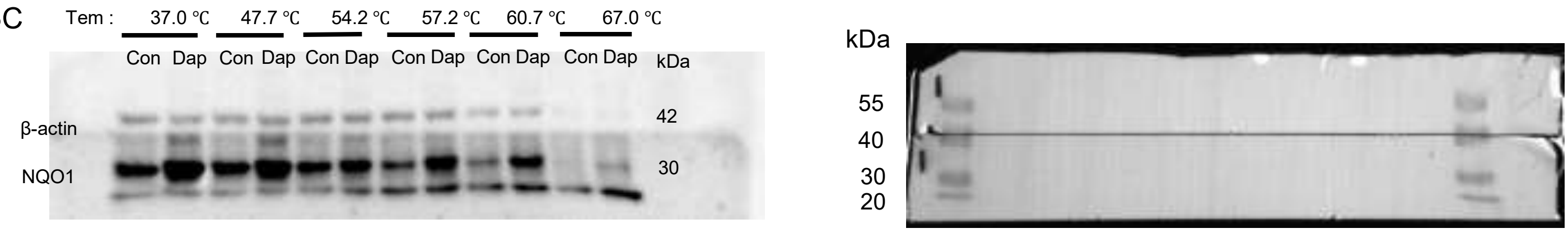

Fig. 6D

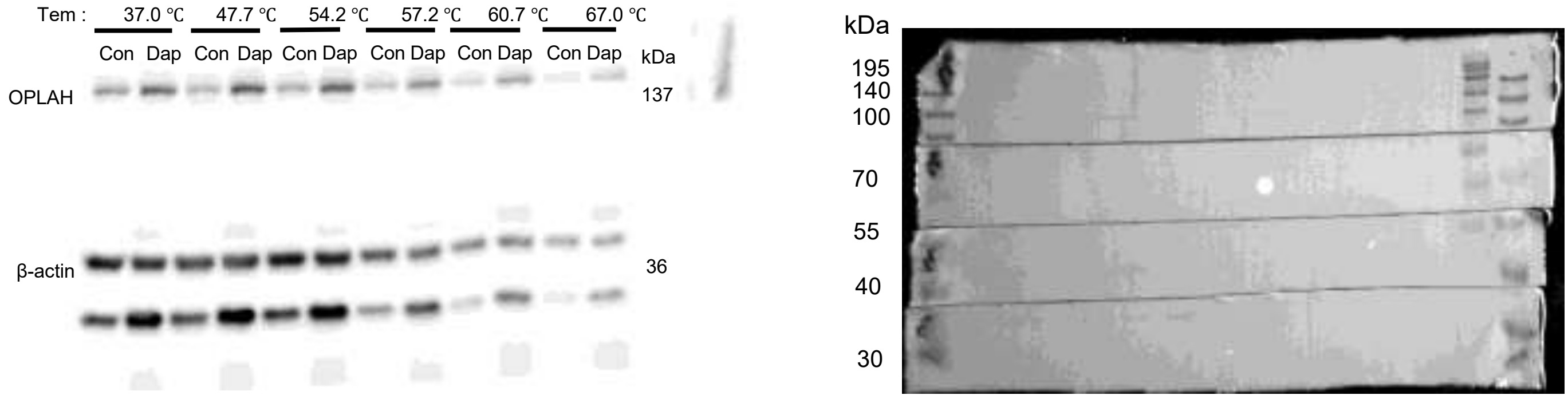

Fig. 6E

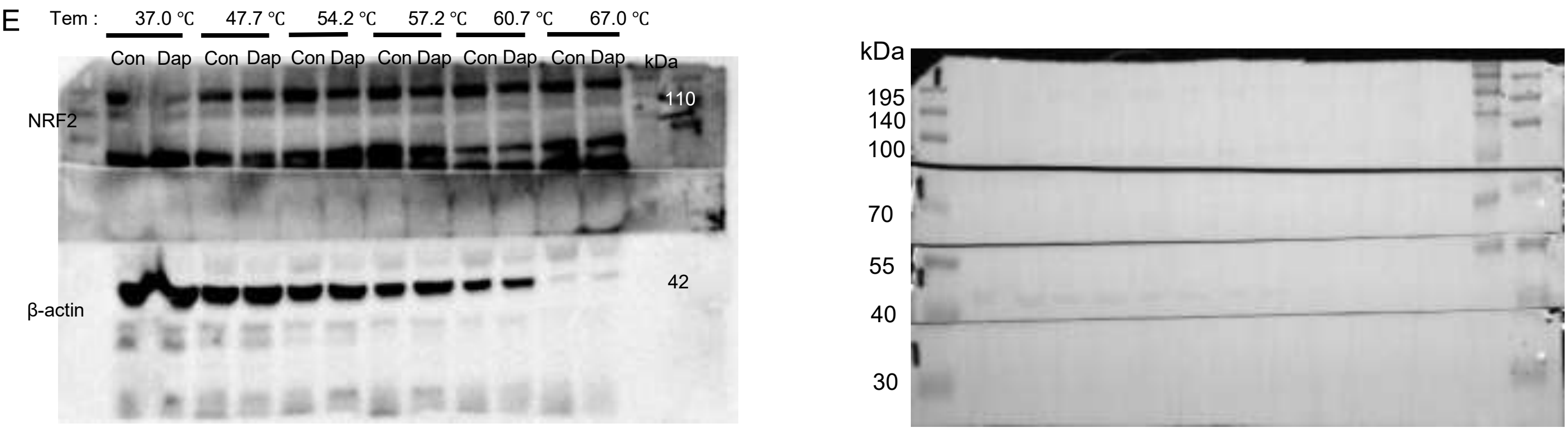

Fig. 6E

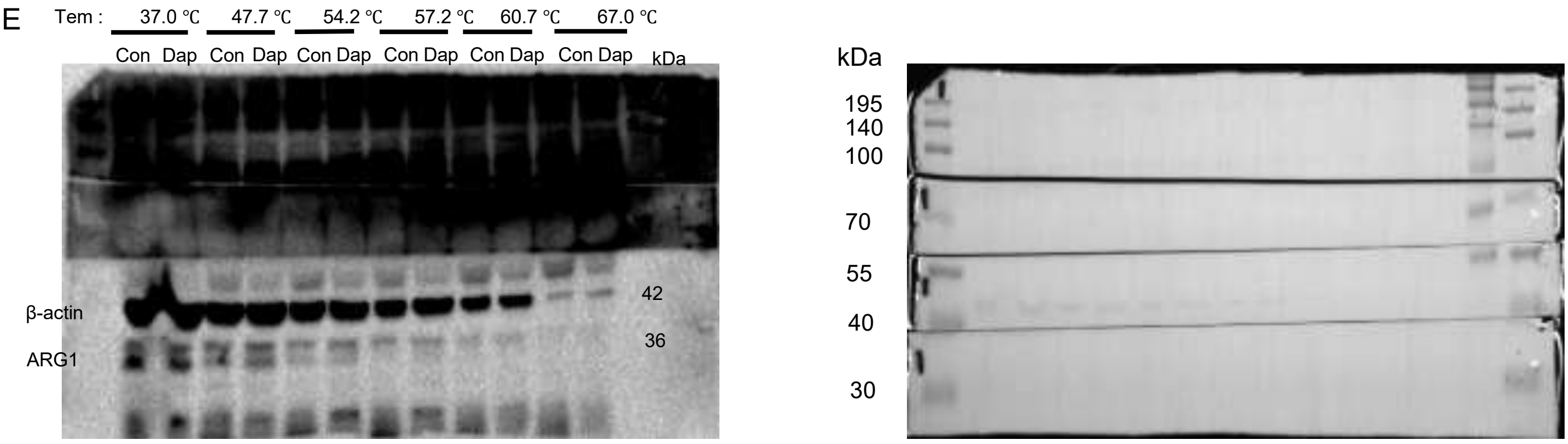

Fig. 7A

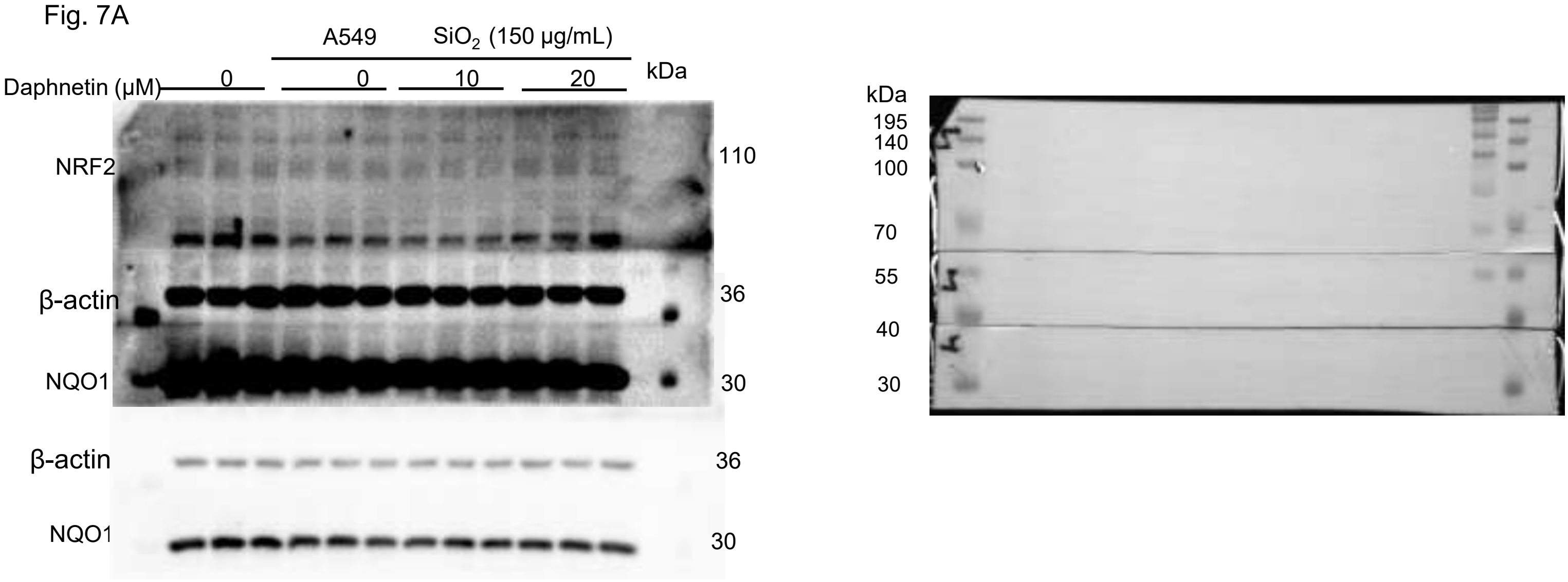

Fig. 7B

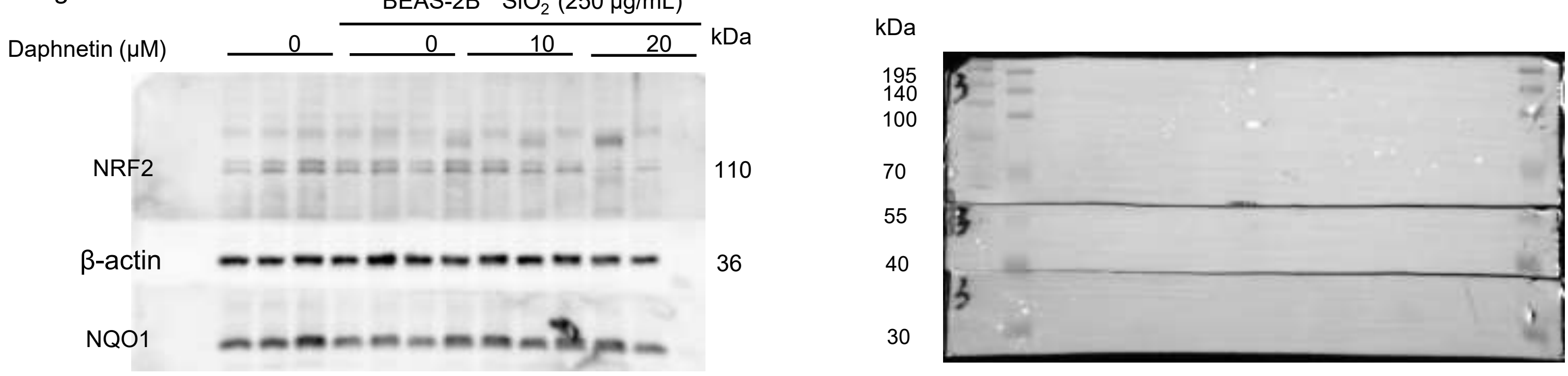

Fig. 7C

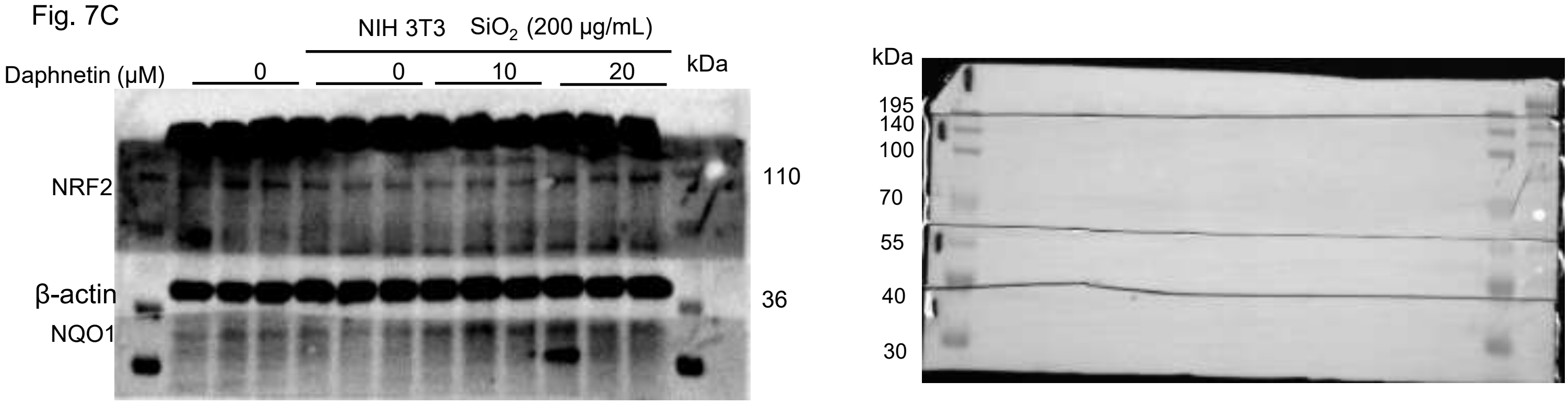

Fig. 7D

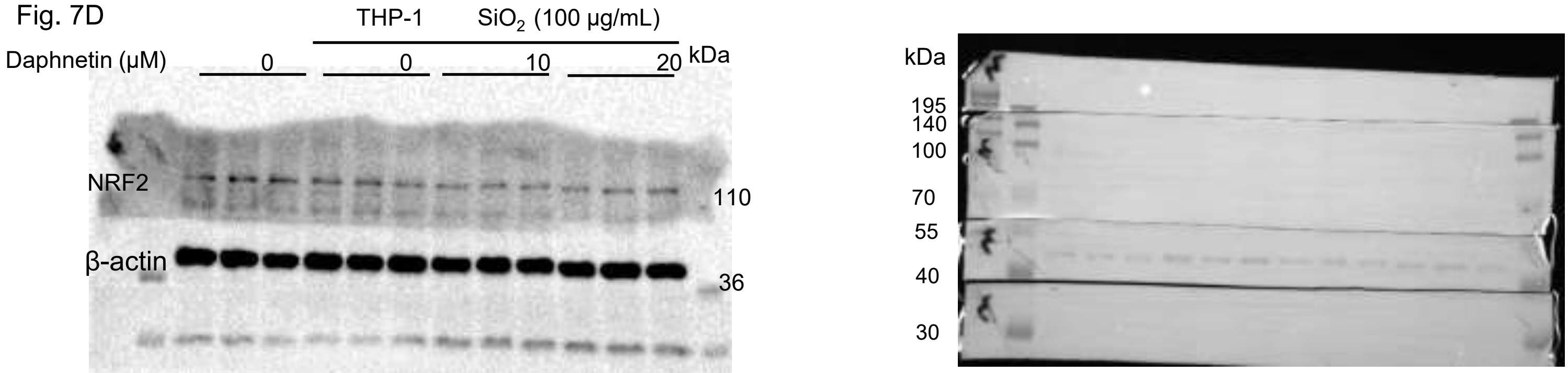

Fig. 7E

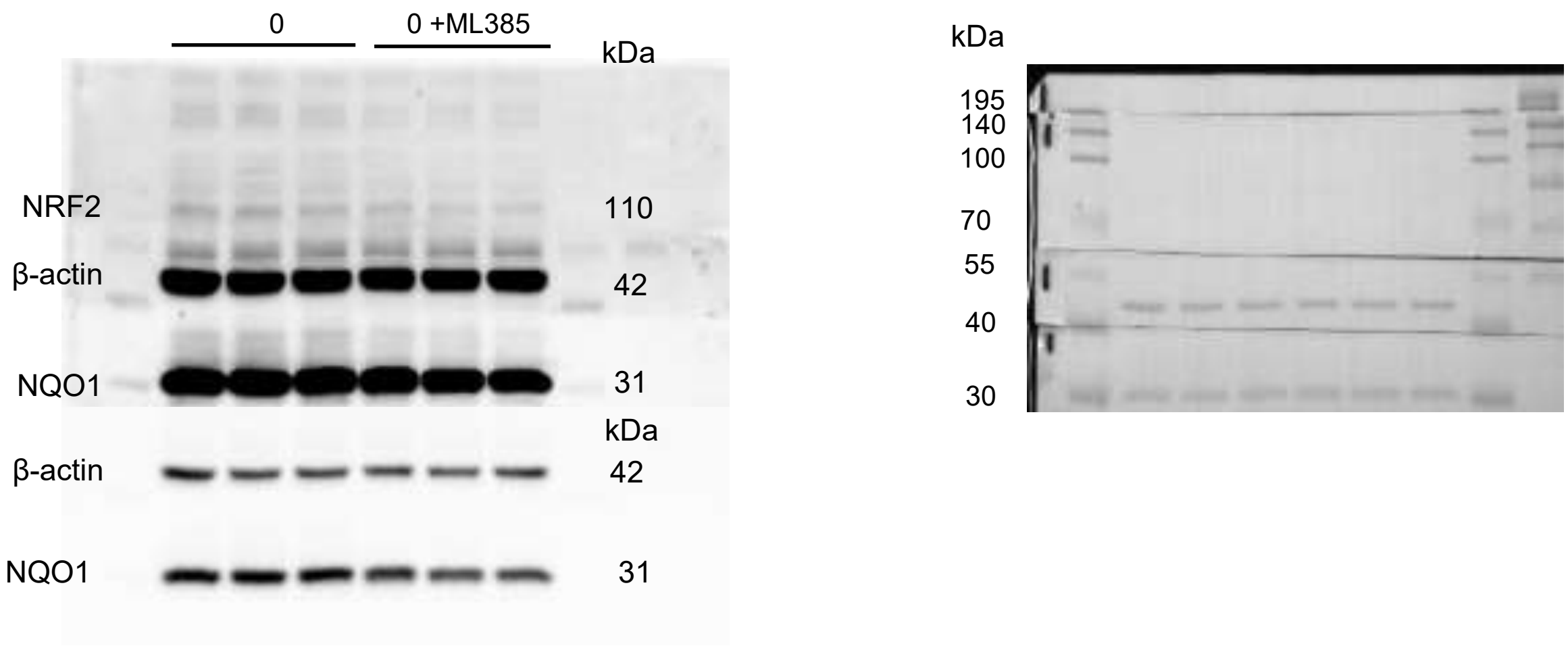

Fig. 7G

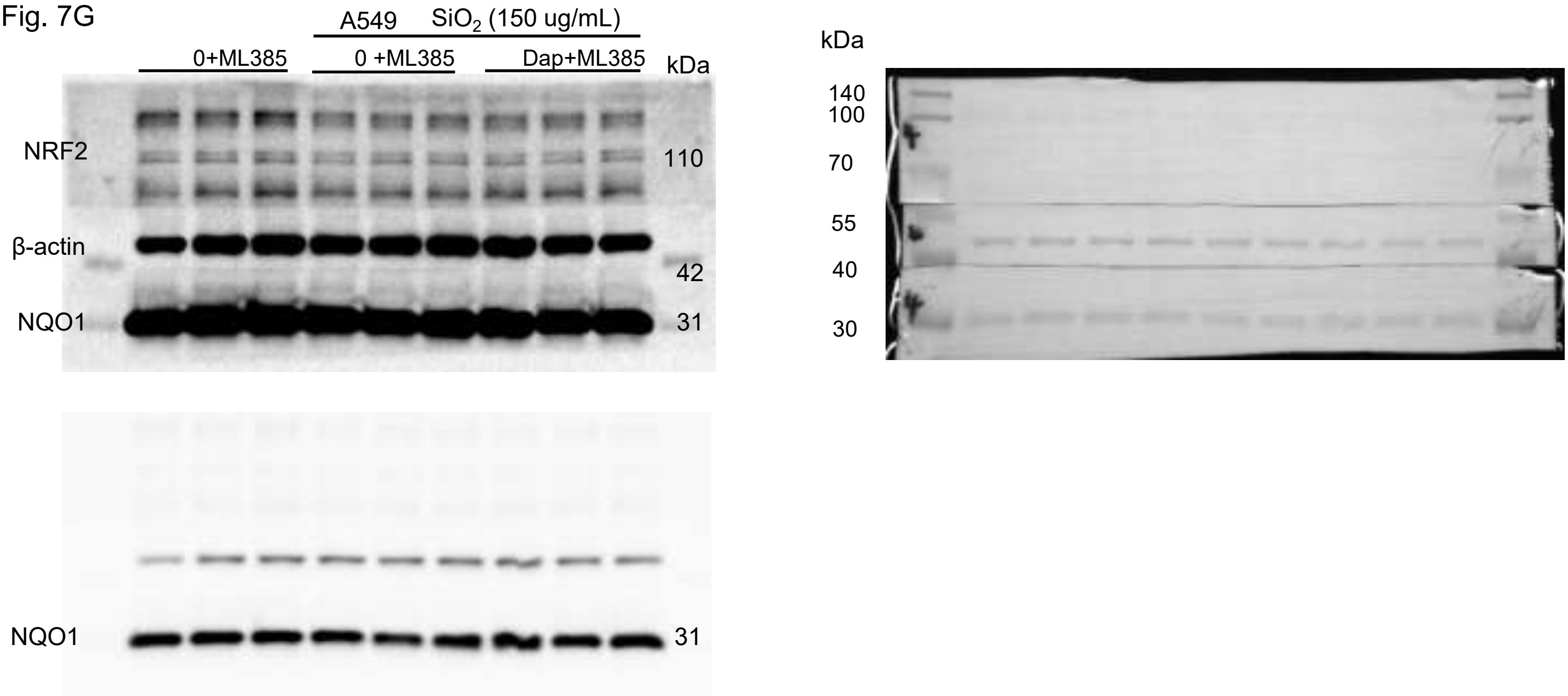

Fig. 8A

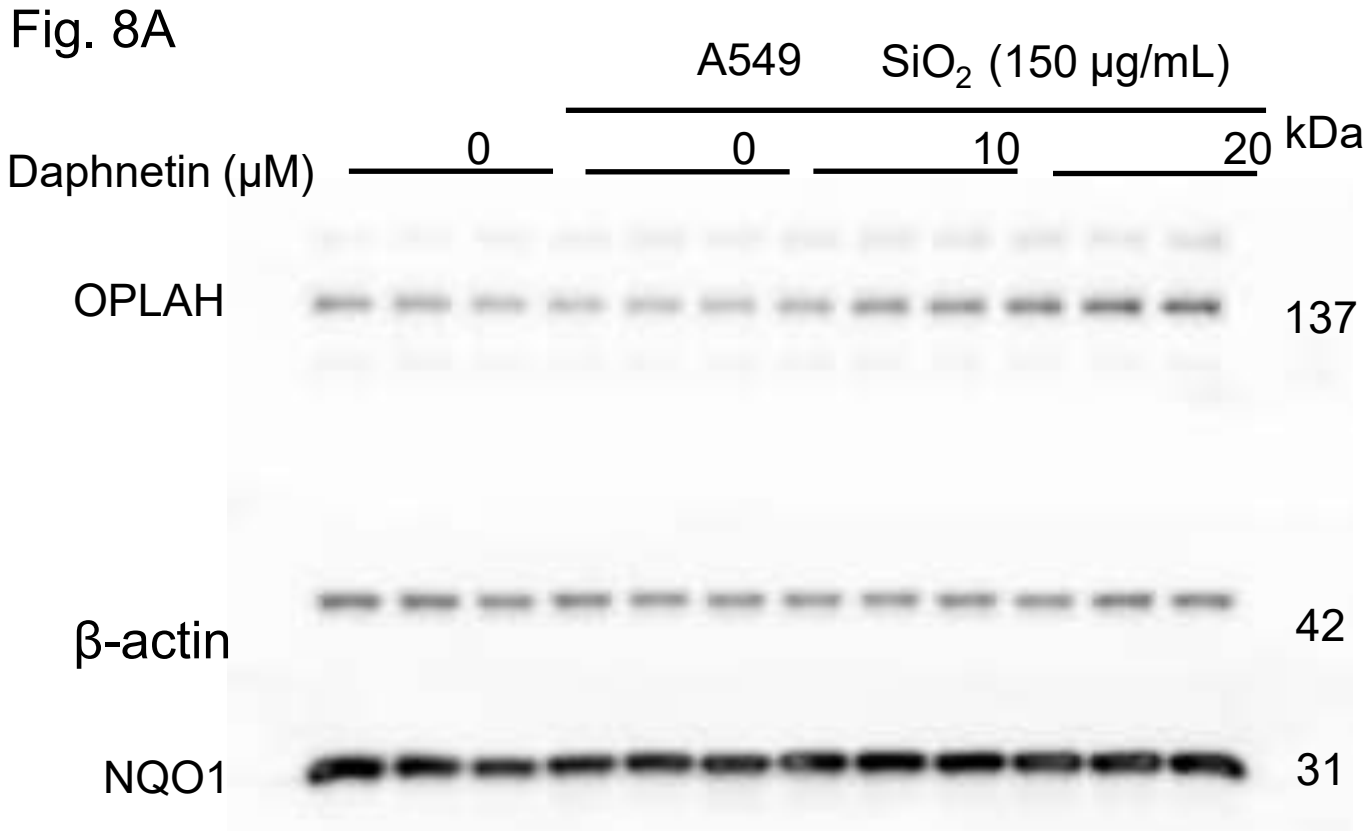

Figure 10A

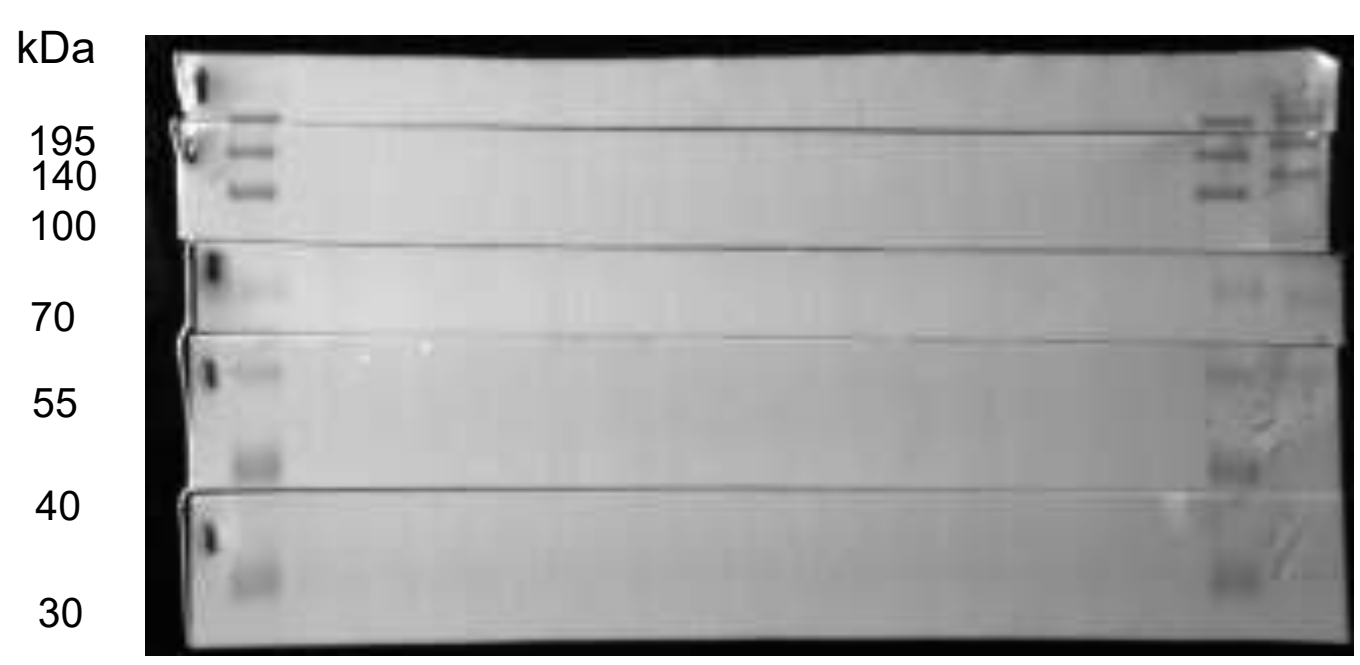

Fig. 8B

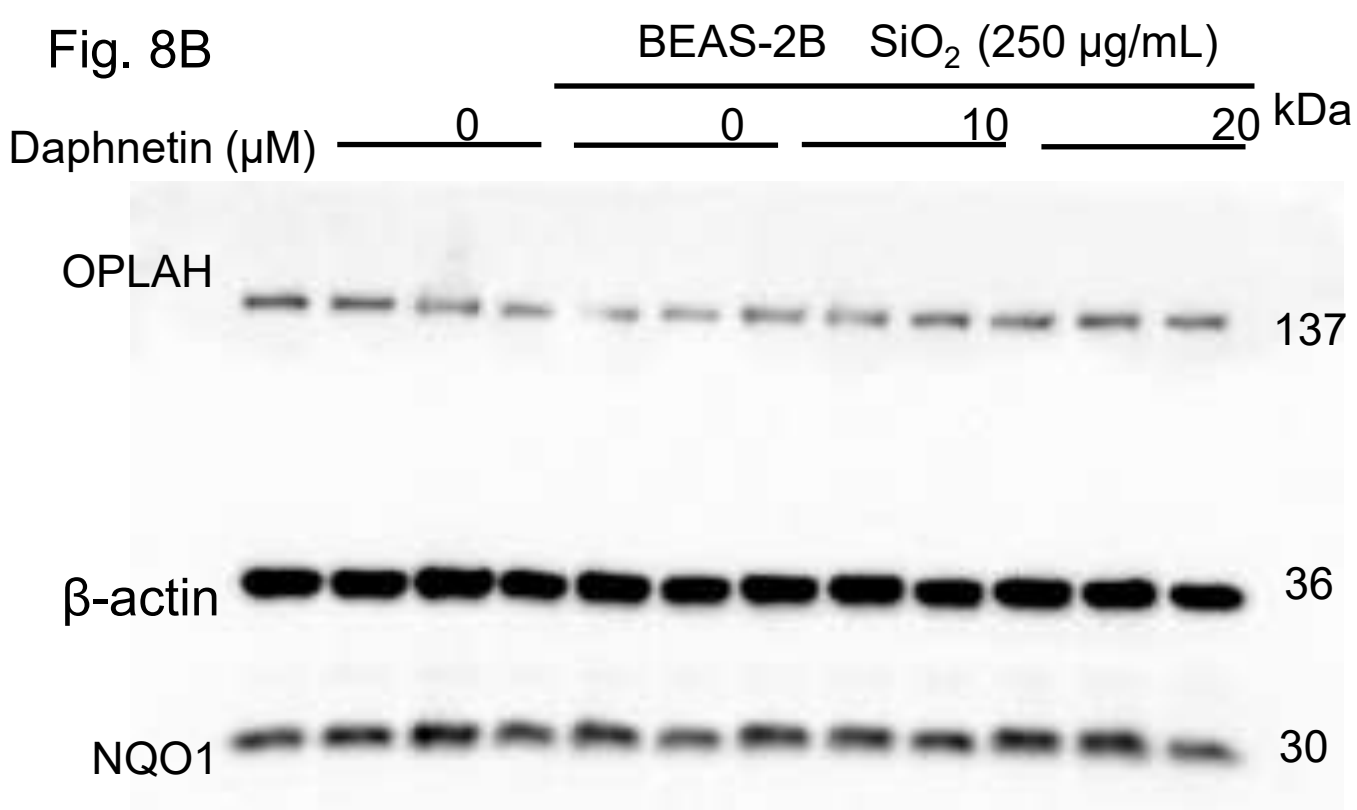

Figure 10B

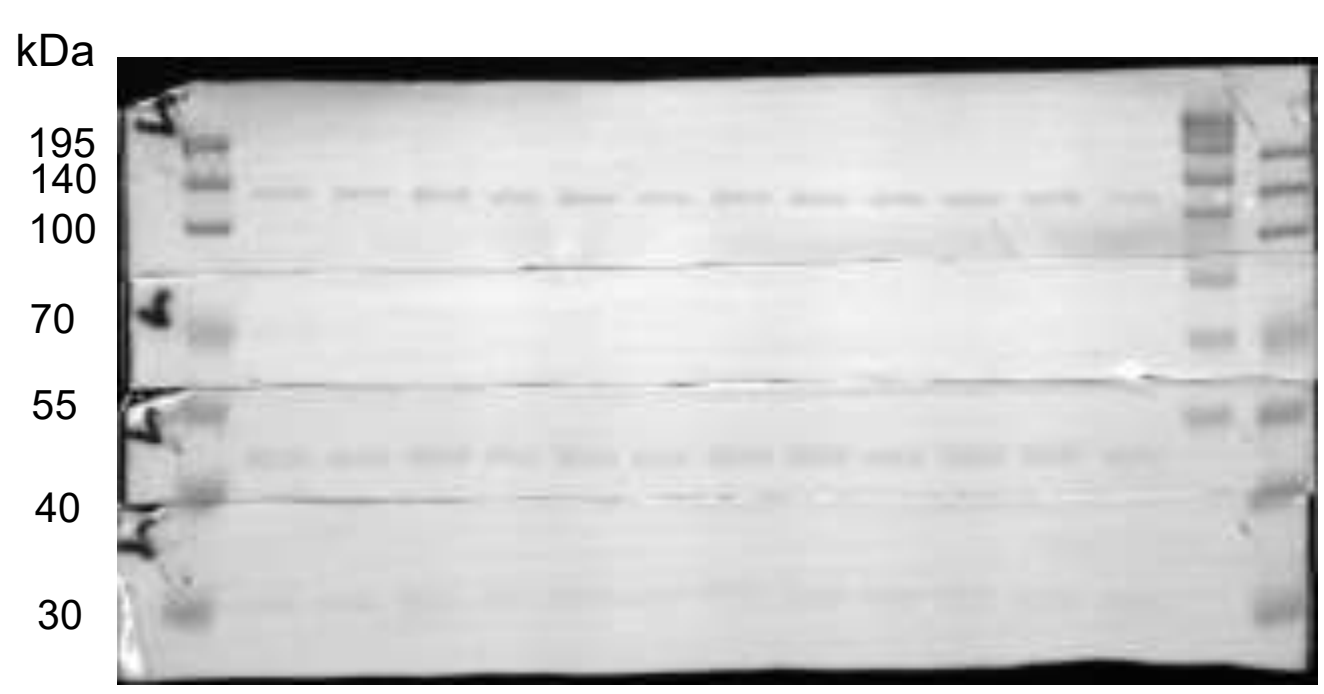

Fig. 8C

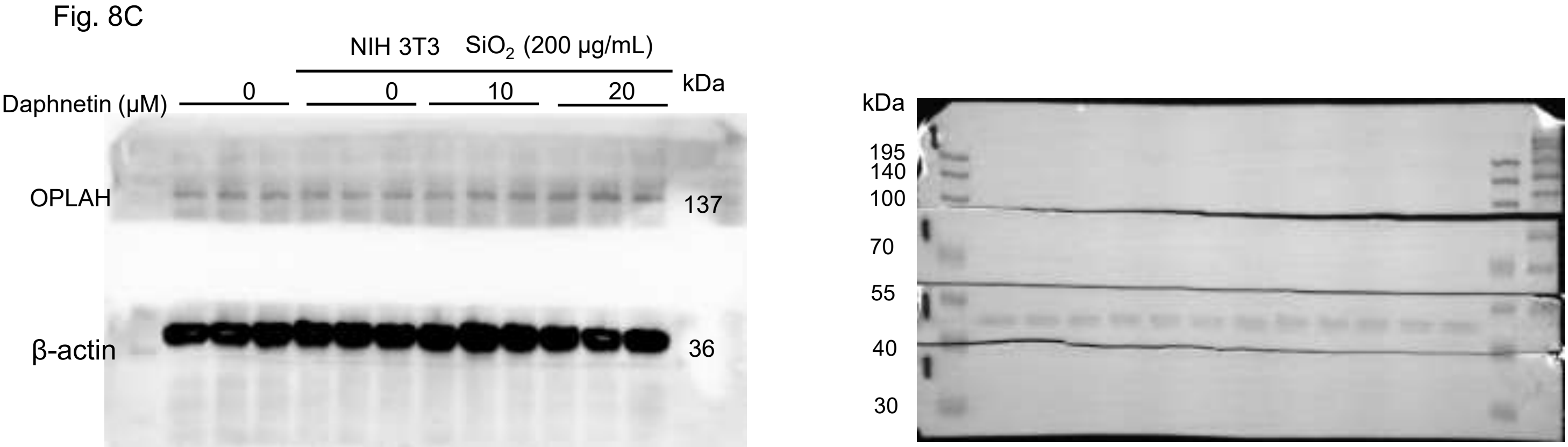

Fig. 8D

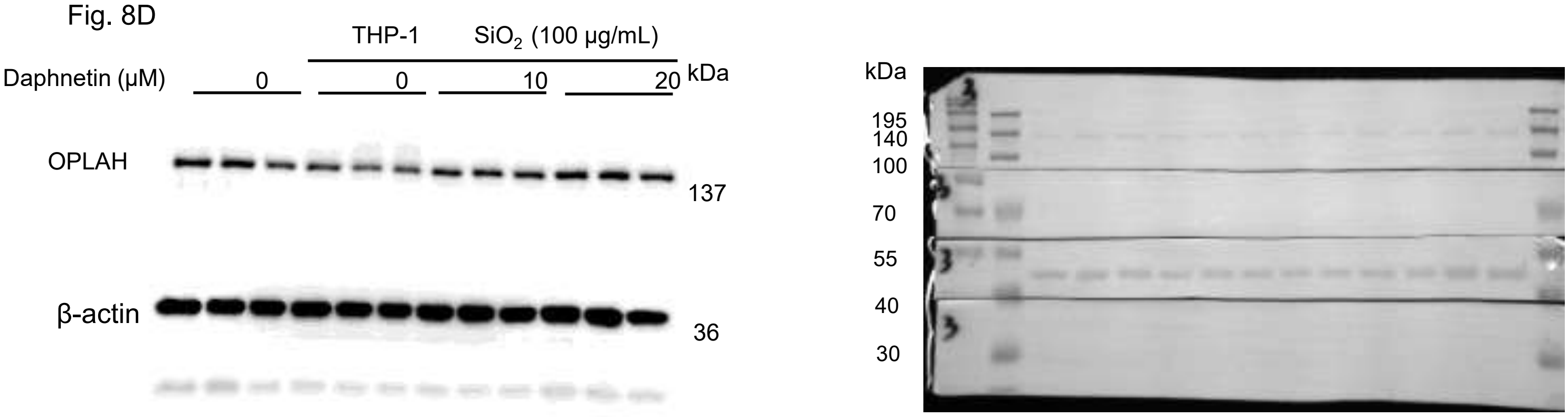

Fig. 8E

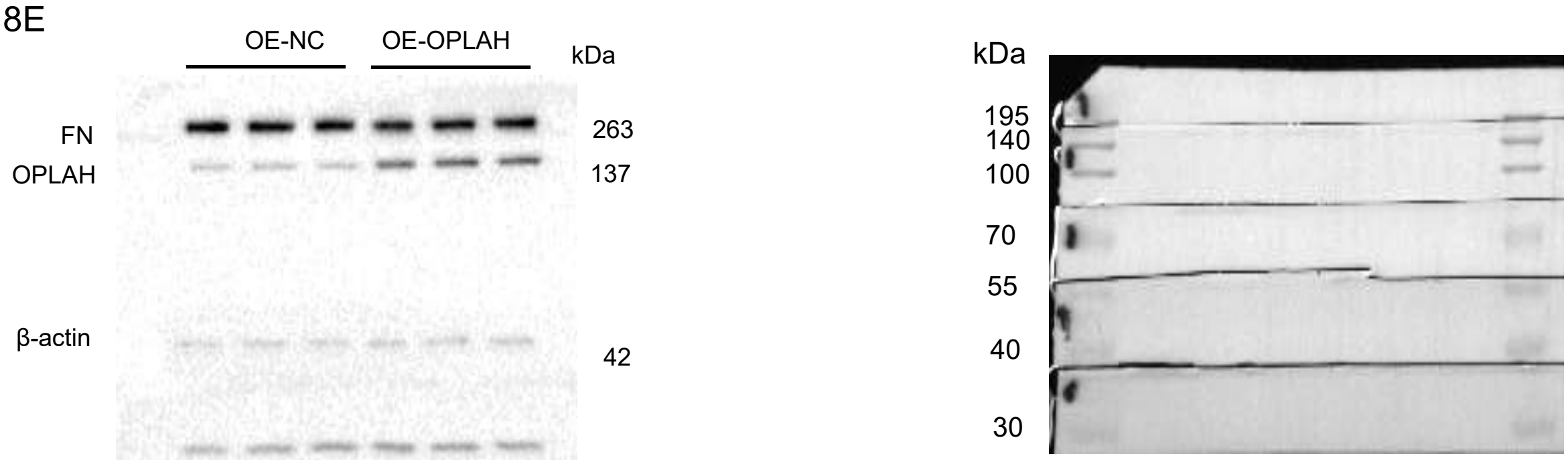

Fig. 8F

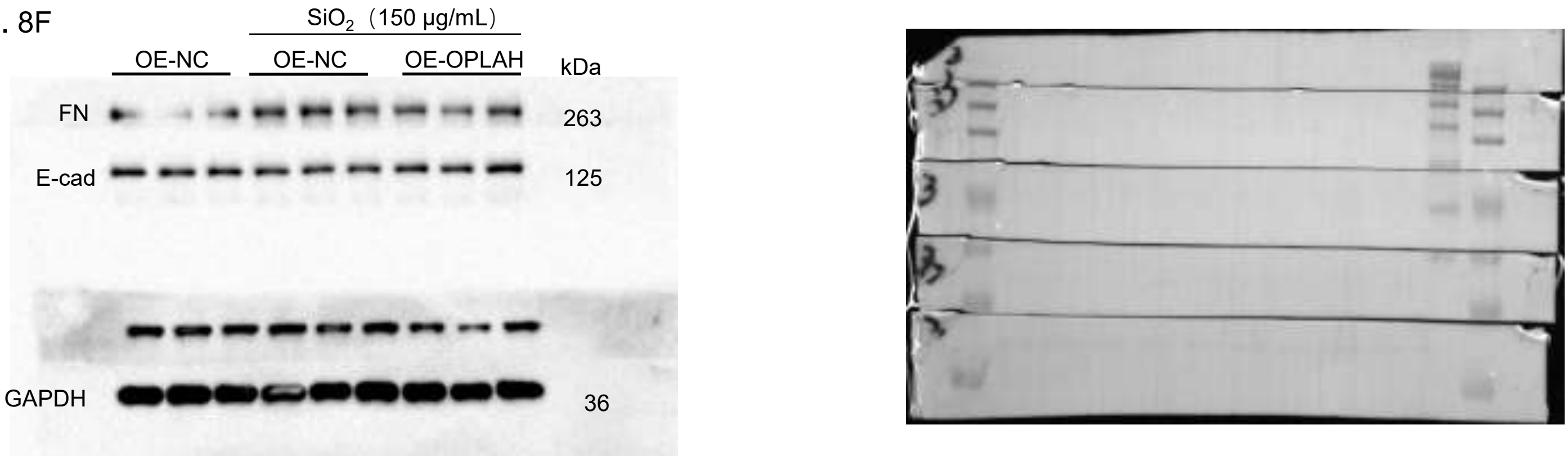

Fig. 8G

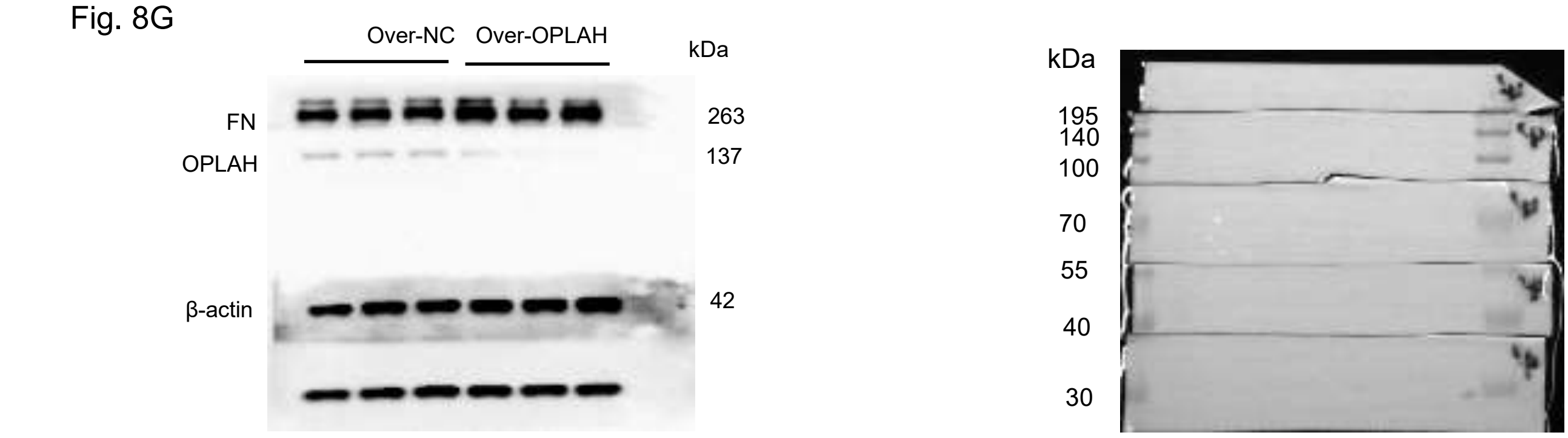
Over-NC
Over-OPLAH
kDa
FN
263
OPLAH
137
β-actin
42
kDa
195
140
100
70
55
40
30

Fig. 8H

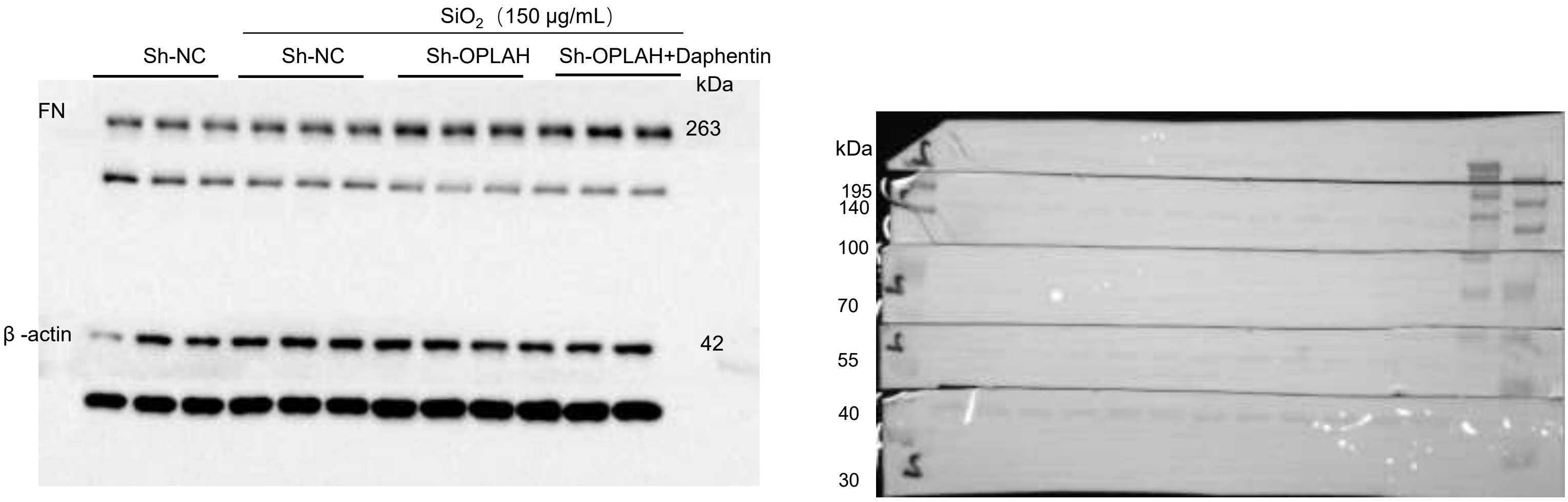
SiO2（150 µg/mL）
Sh-NC
Sh-NC
Sh-OPLAH
Sh-OPLAH+Daphentin
kDa
FN
263
β -actin
42
kDa
195
140
100
70
55
40
30

Fig. S2A

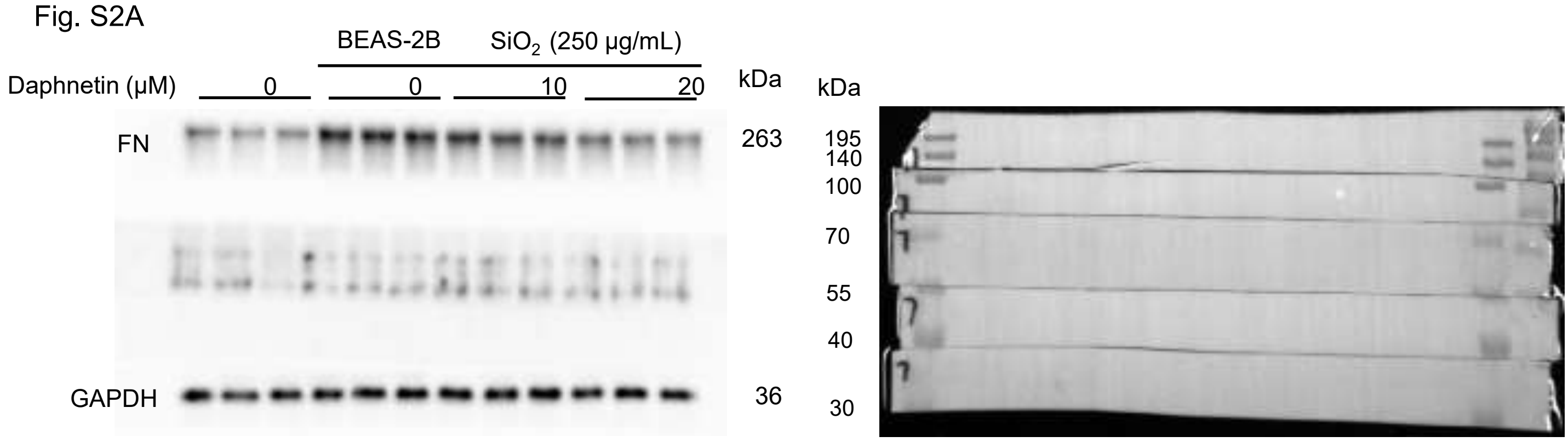

Fig. S2B

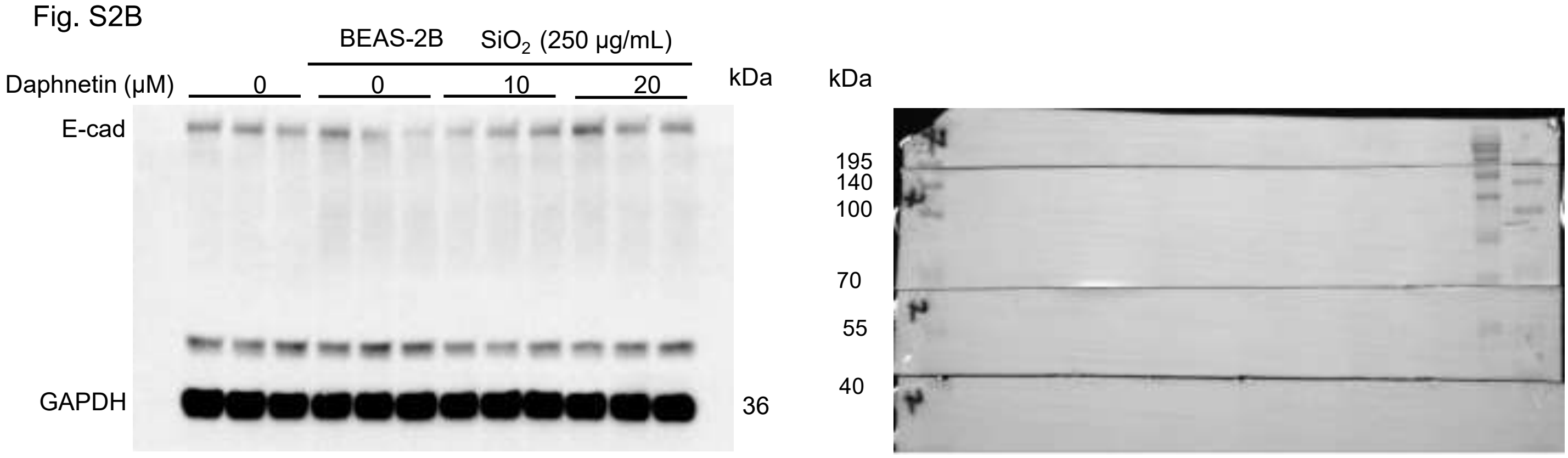

Fig. S2C

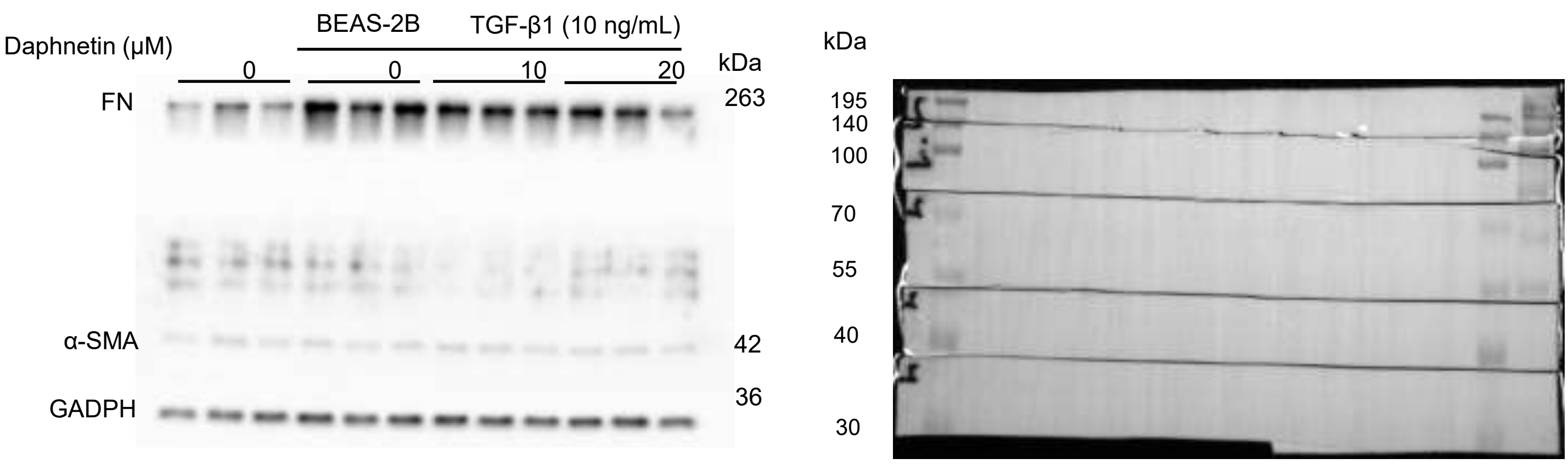

Fig. S2D

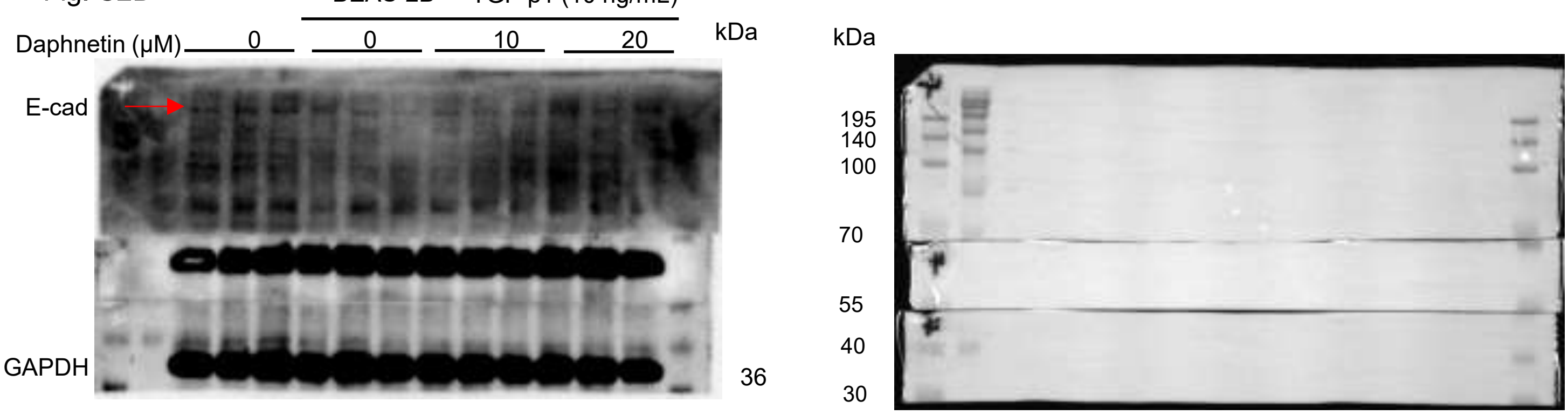

Fig. S6B

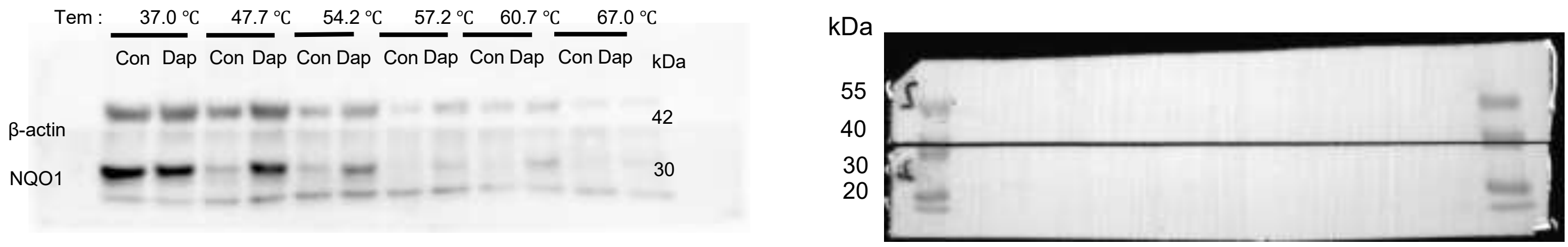

Fig. S6D

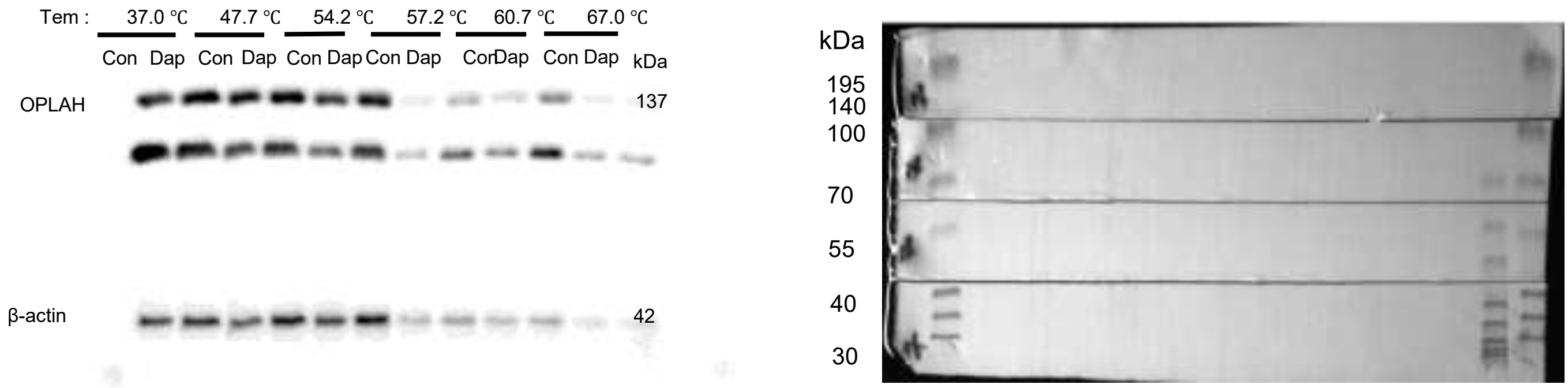

Fig. S6F

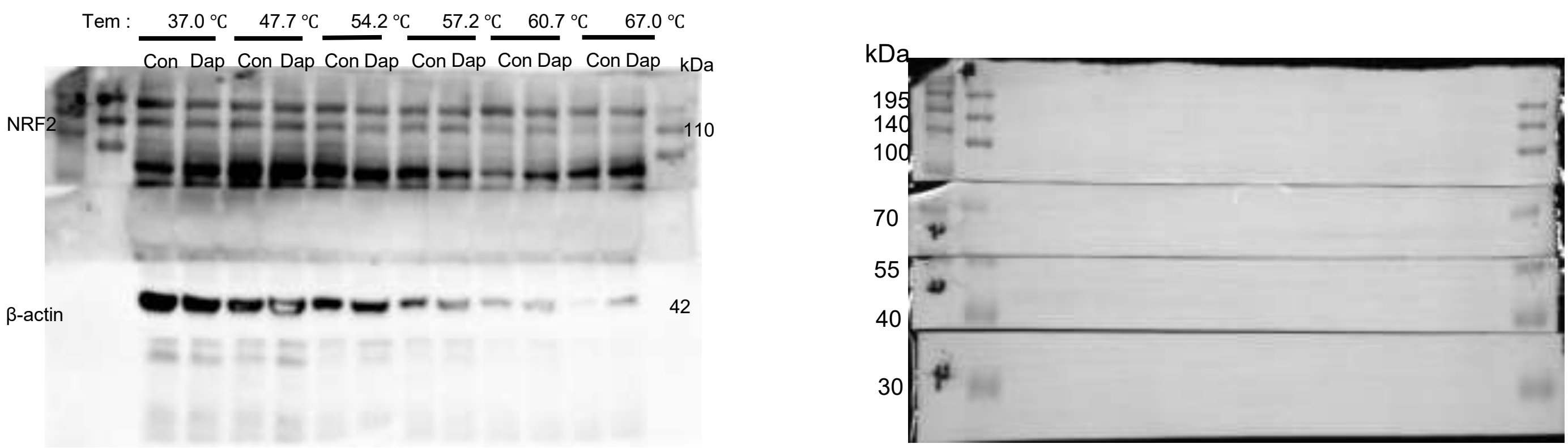

Fig. S6F

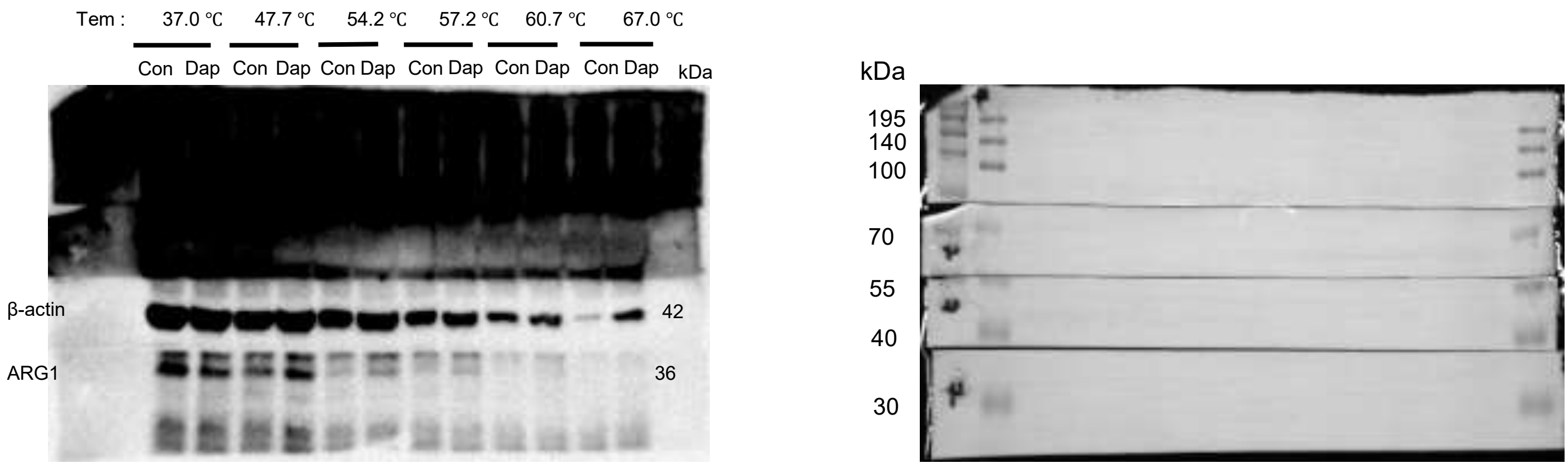

Supplement: Supplementary file 1 [file mmc3.pdf]
