# Supplementary material for: Multi-layered target identification strategy of natural products: Daphnetin targets NQO1 and OPLAH in silicosis alleviation
Source: J Pharm Anal. 2026 Apr 2;16(9):101629. doi: 10.1016/j.jpha.2026.101629 (PMC13602248; doi:10.1016/j.jpha.2026.101629)
Supplement: Supplementary file 2 [file mmc1.docx]

**Supplementary Data**

**S1. Chemicals and materials**

Silicon dioxide (99%, 0.5–10 μm, with approximately 80% between 1–5 μm, Lot No.: S5631-100G) was purchased from Sigma-Aldrich Laboratories, Inc. Daphnetin was purchased in two forms: Lot No.: PS230713–12 (98%, for animal experiments in rats) and Lot No.: F2216293 (99%, for cell culture use), both obtained from Chengdu Push Bio-Technology Co., Ltd. Tetrandrine tablets (Lot No.: PH2206004B, for animal experiments in rats) were purchased from Zhejiang Jinhua CONBA Bio-Pharmaceutical Co., Ltd. Roswell Park Memorial Institute (RPMI) 1640 medium, Dulbecco’s modified Eagle’s medium (DMEM), and Opti-MEM® were purchased from Thermo Fisher Scientific Co., Ltd. (Waltham, MA, USA). Fetal bovine serum was purchased from DCELL Biologics Co., Ltd. (Shanghai, China). Five isotope-labeled internal standards, including cholic acid-2,2,4,4-d4 (CA-d4; Lot No.: 230508), L-valine-d8 (Val-d8; Lot No.: 233023), L-phenylalanine (ring-d5) (Phe-d5; Lot No.: 247149), and stearic acid-18,18,18-d3 (FFA 18:0-d3; Lot No.: 152226), were purchased from MedChemExpress. Nonadecanoyl-2-hydroxy-sn-glycero-3-phosphocholine (LPC19:0; Lot No.: J2214169) was purchased from Aladdin Bio-Chem Technology Co., Ltd. Chromatographic-grade methanol (MeOH), acetonitrile (ACN), and formic acid (FA) were purchased from Thermo Fisher Scientific Co., Ltd. Ultrapure water was purchased from Wahaha Co., Ltd. (Hangzhou, China). Fibronectin (FN; rabbit polyclonal antibody; Lot No.: GB114491-100) and collagen type I (COL-1; rabbit polyclonal antibody; Lot No.: GB11022-3-100) were acquired from Servicebio, Inc. Alpha-smooth muscle actin (α-SMA; rabbit polyclonal antibody; Lot No.: 14395-1-AP), vimentin (VIM; rabbit polyclonal antibody; Lot No.: 00132436), arginase-1 (ARG1; mouse monoclonal antibody; Lot No.: 66129-1-Ig), NAD(P)H quinone oxidoreductase 1 (NQO1; mouse monoclonal antibody; Lot No.: 10041811), β-actin (mouse monoclonal antibody; Lot No.: 10038080), 5-oxoprolinase (OPLAH; rabbit polyclonal antibody; Lot No.: 00095218), GAPDH (mouse monoclonal antibody; Lot No.: 60004-1-Ig), and E-cadherin (E-cad; rabbit polyclonal antibody; Lot No.: 00140274) were purchased from Proteintech Group, Inc. Kelch-like ECH-associated protein 1 (KEAP1; rabbit polyclonal antibody; Lot No.: 107053285) and nuclear factor erythroid 2-related factor 2 (NRF2; rabbit polyclonal antibody; Lot No.: 10302135) antibodies were obtained from Wanleibio. Goat anti-rabbit IgG H&L antibody (Lot No.: BC10268988) and goat anti-mouse IgG H&L antibody (Lot No.: BD10094454) were purchased from Bioss (Beijing, China). Recombinant human transforming growth factor-beta 1 (TGF-β1) and phorbol 12-myristate 13-acetate (Lot No.: 501386) were purchased from MedChemExpress. Isoflurane (Lot No.: 2023071502, for veterinary use) was obtained from Shenzhen REAWORL Life Technology Co., Ltd. (Shenzhen, China). ML385 (purity ≥99%) was purchased from Aladdin Inc. (Shanghai, China). SevenHighTrans™ DNA non-liposomal transfection reagent (Lot No.: 25FI0077) was purchased from Seven Inc. (Beijing, China). Reactive oxygen species assay kit (Lot No.: 10A065) and reduced glutathione (GSH) detection kit were obtained from Wanleibio (Shenyang, China). Hoechst 33342 probe (Lot No.: C0031) was purchased from Servicebio, Inc. (Wuhan, China).

**S2 Histological and immunohistochemical analysis of lung tissue**

*S2.1 Lung histological analysis*

The fixed lung tissues were used for Hematoxylin-Eosin (HE), Masson’s trichrome, and Sirius Red staining. In brief, the samples were dehydrated, embedded, and cut into 4-μm-thick sections. The staining solutions from the Hematoxylin–Eosin Stain Kit (Solarbio, Beijing, China), Masson’s Trichrome Stain Kit (Solarbio, Beijing, China), and Modified Sirius Red Stain Kit (Solarbio, Beijing, China) were then added to the sections for staining, respectively. Eight fields of view were captured from each pathology section using a light microscope and analyzed using ImageJ software. Specifically, whole-slide scanning of the Masson’s trichrome-stained sections was conducted to analyze the proportion of nodular areas. The Szapiel score was based on the results of HE staining.

*S2.2 Lung Immunohistochemical staining*

The lung tissue sections were deparaffinized with dimethylbenzene and antigen retrieval was performed using Tris–EDTA (pH 8.0, Servicebio, wuhang, China). The sections were incubated with 3% H_2_O_2_ at room temperature for 10 min to eliminate the activity of endogenous peroxidase. After incubation with 5% BSA for 45 min, the tissue sections were treated with primary antibodies specific to fibronectin (FN, 1:50000), alpha-smooth muscle actin (α-SMA, 1:1800), collagen type I (COL-1, 1:800), and vimentin (VIM, 1:12500) overnight at 4 °C, followed by incubation with a secondary antibody for 1 h at room temperature. After using a 3,3’-diaminobenzidine (DAB) kit for color development, the sections were counterstained with hematoxylin for 5 min, dehydrated, and mounted for imaging by light microscopy. The proportion of positive areas in each field of the histological slides was analyzed using ImageJ software.

**S3 Detection of Daphnetin-related metabolites in lung tissue and serum**

*S3.1 Sample preparation and extraction*

Serum and lung tissue samples were prepared using a liquid-liquid extraction method. For serum, 50 μL of serum was mixed with 15 μL of 0.1% formic acid (*v/v*) and vortexed for 1 min. After adding 500 μL of ethyl acetate, the mixture was vortexed for 5 min and centrifuged at 13,000 r/min to separate the organic phase, which was then dried under nitrogen at 40°C. The residue was dissolved in 50 μL of methanol and centrifuged at 13000 r/min for 10 min. The supernatant (5 μL) was used for analysis. For lung tissue, approximately 50 mg of tissue was homogenized with 300 μL of physiological saline and 30 μL of 0.1% formic acid. After adding 1.5 mL of ethyl acetate, the mixture was vortexed for 5 min and centrifuged as described for serum. The organic phase was collected, dried under nitrogen, and re-dissolved in 50 μL of methanol before centrifugation. The supernatant (5 μL) was analyzed.

*S3.2 Liquid chromatography-tandem mass spectrometry (LC-MS/MS) conditions*

The LC-MS analysis was performed using a1290 Infinity II liquid chromatography system (Agilent Technologies, Santa Clara, CA, USA) coupled with a TripleTOF 5600+ time-of-flight mass spectrometer (AB Sciex, Framingham, MA, USA). Separation was achieved on a Waters Acquity UHPLC HSS T3 column (2.1 mm × 100 mm, 1.8 μm) at 30°C, with an injection volume of 5 µL and a flow rate of 0.3 mL/min. The mobile phases consisted of 0.1% formic acid (A) in water and (B) acetonitrile, and the gradient program was as follows: 0 min, 98% A; 0−1.5 min, 98% to 10% A; 1.5−2.5 min, 10% A; 2.5−3.5 min, 10% to 98% A; and 3.5−6 min, 98% A. The electrospray ionization (ESI) source was operated in both negative and positive ion modes, with the following parameters: ion spray voltage of −4500 V (negative), turbo spray temperature of 450°C, nebulizer gas (Gas 1) at 55 psi, heater gas (Gas 2) at 55 psi, curtain gas (CUR) at 30 psi, declustering potential (DP) at 60 V, and collision energy (CE) set to 35 eV with a CE spread of 15 eV.

**S4 Pseudotargeted metabolomics analysis**

*S4.1 Sample preparation and extraction*

30 mg of lung tissue were weighed and added into 600 μL of precooled MeOH: ACN: H_2_O (5:3:2, *v*/*v*/*v*). Then homogenized the mixture for 3 min at 60 Hz, and extracted by ultrasonicating in an ice-water bath for 10 minutes, followed by incubation at 4 °C for 1 h. After centrifugation at 13,000 r/min for 15 mins at 4 °C, 800 μL of the supernatant was collected and evaporated to dryness in vacuum evaporation, and reconstructed with 100 μL of pre-cooled 20 % acetonitrile containing of 4.60 μg/mL L-Val-d8, 4.78 μg/mL Phe-d5, 4.86 μg/mL CA-d4, 2.87 μg/mL FFA 18:0-d3 and 2.85 μg/mL LPC19:0, followed by ultrasonication in an ice-water bath for 10 minutes. The sample was then centrifuged at 13,000 r/min for 15 min at 4 °C, and an appropriate amount of the supernatant was taken for subsequent mass spectrometry analysis. To ensure the stability and repeatability of the experiments, 10 μL each sample were pooled together as a quality control (QC) sample.

*S4.2 LC-MS/MS conditions for comprehensive pseudotargeted metabolomics*

Comprehensive pseudotargeted metabolomics data were acquired using an AB SCIEX Triple Quad 5500 linear ion trap triple quadrupole mass spectrometer (AB Sciex) coupled to an ExionLC AD UHPLC system (Shimadzu, Kyoto, Japan). Chromatographic separation was achieved on a Waters Acquity UHPLC HSS T3 column (2.1 mm × 100 mm, 1.8 μm) maintained at 40 °C, with an injection volume of 1 μL. The mobile phases consisted of 0.1% formic acid in water (solvent A) and acetonitrile (solvent B), delivered at a flow rate of 0.3 mL/min. The gradient elution program was as follows: 0−2 min, 2% B; 2−3 min, 2% to 35% B; 3−17 min, 35% to 70% B; 17−18 min, 70% B; 18−20 min, 70% to 98% B; 20−23 min, 98% B; 23−23.5 min, 98% to 2% B; and 23.5−25 min, 2% B. Mass spectrometry was conducted using an electrospray ionization (ESI) source in both positive and negative ion modes, with the following settings: ion spray voltage of +5500 V (positive mode) and −4500 V (negative mode), ion source temperature of 550 °C, curtain gas (CUR) at 30 psi, nebulizer gas (Gas 1) at 55 psi, and heater gas (Gas 2) at 55 psi. MRM cycle time was 1 s.

*S4.3 LC-MS/MS conditions for animo acids*

The amino acid metabolomic profiling analysis was performed using an AB SCIEX Triple Quad 3200 linear ion trap triple quadrupole mass spectrometer (AB Sciex) coupled to an ExionLC AD UHPLC system. A Waters Acquity UHPLC BEH C18 column (2.1 mm × 100 mm, 1.7 μm) was used for separation at a column temperature of 30 ℃. The injection volume was 3 µL. For metabolomics analysis, the mobile phases consisted of 0.1 % formic acid aqueous solution (A) and acetonitrile (B). The flow rate was 0.2 mL/min and gradient elution was optimized as follows: 0−1 min, 0−3 % B; 1−1.8 min, 3 % B; 1.8−3 min, 3−6 % B; 3−5 min, 6−25 % B; 5−7.5 min, 25−60 % B;7.5−10 min, 60−0 % B; 10−11 min, 0 % B. The parameter settings of the positive electron spray ionization (ESI +) source were as follows: ion spray voltage was set as 5500 V, turbo spray temperature 550℃, nebulizer gas (Gas 1) 45 psi, heater gas (Gas 2) 50 psi, curtain gas (CUR) 40 psi.

**S5 Western blotting analysis**

Lung tissue and cells were lysed using precooled enhanced radioimmunoprecipitation assay (RIPA) buffer containing 1% phenylmethylsulfonyl fluoride (PMSF) to extract total protein. The supernatant was then used to measure protein concentration and was quantified to 2 μg/μL. Twenty micrograms of protein were loaded onto an 8% SDS-polyacrylamide gel, which was electrophoretically transferred to PVDF membranes (Millipore, MMAS, USA) after electrophoresis. The membranes were blocked and incubated at 4°C overnight. The primary antibodies used in this study included fibronectin (FN, 1:1000), α-smooth muscle actin (α-SMA, 1:1000), E-cadherin (E-cad, 1:15000), arginase-1 (ARG1, 1:1000), NAD(P)H quinone dehydrogenase 1 (NQO1, 1:1000), kelch-like ECH-associated protein 1 (KEAP1, 1:1000), nuclear factor erythroid 2-related factor 2 (NRF2, 1:1500), 5-oxoprolinase (OPLAH, 1:1000), β-actin (1:20000), and glyceraldehyde-3-phosphate dehydrogenase (GAPDH, 1:20000). The secondary antibody (1:20000) was added and incubated for 1.5 h, after which the protein signals were collected by chemiluminescence (ChemiDoc™ XRS+, Bio-Rad), and the band densities were quantified using Image Lab software.

**S6 Computational Biology Methods**

*S6.1 Molecular Docking*

According to the previous study, molecular docking of daphnetin with NAD(P)H quinone dehydrogenase 1 (NQO1) or 5-oxoprolinase (OPLAH) proteins was performed using AutoDock Vina (Discovery Studio v2.5, San Diego, CA, USA). Validated NQO1 and OPLAH protein structures were preprocessed in AutoDockTools by removing ligands, water molecules, and other non-essential components. The three-dimensional structure of daphnetin (*.sdf) was retrieved from the PubChem database. Docking sites on the proteins were defined using AutoDockTools. For the NQO1-daphnetin complex, the grid box was centered at x = 20.914, y = -18.033, z = -29.115, with dimensions of 85.25 × 96.1 × 83.7 Å. For the OPLAH-daphnetin complex, the grid box was centered at x = -0.838, y = 3.489, z = 2.452, with dimensions of 91.35 × 87.0 × 91.35 Å. The exhaustiveness parameter was set to 300 for all docking runs.

*S6.2 Molecular Dynamics (MD) Simulations*

To further assess the stability of protein-ligand interactions, MD simulations were performed using GROMACS 2022.2. The CHARMM36 force field was used, and topologies for both proteins and small molecules were generated using CHARMM-GUI. Simulations were initiated based on the docked protein-ligand complex conformations. Each system was solvated in a TIP3P water model within an octahedral box, ensuring a minimum distance of 9.4 nm from the solute to the box edge. Energy minimization was carried out using the steepest descent algorithm until the maximum force fell below 1000 kJ/mol/nm. Equilibration was then performed under NVT (constant number of particles, volume, and temperature) and NPT (constant number of particles, pressure, and temperature) ensembles, using the V-rescale and C-rescale algorithms to maintain the temperature at 303.15 K and the pressure at 100 kPa, respectively. A time step of 2 fs was applied, and the LINCS algorithm was used to constrain bonds involving hydrogen atoms. Trajectory analyses over 100 ns were performed in GROMACS to calculate the root mean square deviation (RMSD), root mean square fluctuation (RMSF, for all atoms and backbone atoms), hydrogen bond dynamics, radius of gyration (Rg), solvent-accessible surface area (SASA), per-residue energy decomposition, and interaction energies.

**S7	Quantitative real-time polymerase chain reaction (qRT-PCR)**

Total messenger ribonucleic acid (mRNA) of lung tissues or cells was extracted using the TRIzol reagent (Sangon Biotech, shanghai, China). RNA quality and concentration were measured by QuickDrop Spectrophotometer (MolecularDevices, San Jose, USA). cDNAs were synthesized from total mRNA using All-In-One 5X RT MasterMix Kit (Abm, Vancouver, Canada). β-actin was used as the reference gene in quantitative real-time PCR assay. The primers used were listed in Table S3. Quantitative real-time PCR analysis was analyzed using the BlasTaqTM 2X qPCR Master Kit (Abm, Vancouver, Canada) and performed on Heal force trident 960 real-time PCR system (shanghai, China). The relative expression of target genes was calculated by the 2^−ΔΔ^*^CT^* method.

**Fig. S1.** Effects of daphnetin on silicotic rats and its metabolism in lung tissue and serum. (A) Body weight (n = 9). (B) Average food intake (n = 9). (C−H) Liquid chromatography-tandem mass spectrometry (LC-MS/MS) detection of daphnetin and its main metabolites in the serum and lung tissue of daphnetin (60 mg/kg) group, (C) Daphnetin in lung, (D) Daphnetin-O-methyl in lung, (E) Daphnetin-O-sulfonate in lung, (F) Daphnetin in serum, (G) Daphnetin-O-methyl in serum, (H) Daphnetin-O-sulfonate in serum.

**Fig. S2.** Effect of daphnetin on epithelial-mesenchymal transition in BEAS-2B cells induced by silica or transforming growth factor-β1 (TGF-β1) (n = 3). (A, B) Expression of fibronectin (FN) (A) and E-cadherin (E-cad) (B) in BEAS-2B cells exposed to silica (250 μg/mL). (C) Expression of FN and α-smooth muscle actin (α-SMA) treated with TGF-β1 (10 ng/mL). (D) Expression of E-cad cells treated with TGF-β1 (10 ng/mL). (E) Representative images and quantitative analysis of the scratch wound healing assay in BEAS-2B cells treated with TGF-β1. ^*^*P* *<* 0.05, ^**^*P* *<* 0.01, and ^*^*P* *<* 0.001 vs. control group; ^#^*P* *<* 0.05, ^##^*P* *<* 0.01, and ^###^*P* *<* 0.001 vs. model group.

**Fig. S3.** Construction of the daphnetin gene expression signature and pneumoconiosis-related enzymes. (A) Network diagram of 57 efficacy targets of daphnetin. (B) Schematic diagram of functional representation of gene signatures (FRoGS), a deep learning model for gene feature-based target prediction. ShRNA: small hairpin RNA; cDNA: complementary DNA.

**Fig. S4.** Target identification for daphnetin using metabolomics. (A) Kyoto Encyclopedia of Genes and Genomes (KEGG) pathway analysis of 202 pneumoconiosis-related enzymes. (B, C) The typical chromatograms in the positive (B) and negative (C) ion modes, respectively. (D) Heatmaps of differential metabolites in positive (right) and negative (left) ion modes across different groups. (E) KEGG pathway analysis of the 55 daphnetin-associated metabolites. (F) Principal component analysis (PCA) distribution plot of quality control (QC) samples after combat correction. (G) Distribution bar chart of common metabolites detected in both batches after ComBat correction. (H) Bar plots showing the levels of ornithine, citrulline, valine, glutamate, 5-oxoproline, serine, asparagine and spermine. ^*^*P* *<* 0.05, ^**^*P* *<* 0.01, and ^***^*P* *<* 0.001 vs. control group; ^#^*P* *<* 0.05, ^##^*P* *<* 0.01, and ^###^*P* *<* 0.001 vs. model group.

**Fig. S5.** Construction of the daphnetin efficacy target network and screening of thermostable proteins. (A) Venn diagram showing the overlap between transcriptomic-derived and metabolite–enzyme-derived targets. (B) Network of 142 efficacy targets of daphnetin. (C) Protein thermal stability gel images of lung tissue after oral daphnetin (20 mg/kg) administration at different temperatures.

**Fig. S6.** Cellular thermal shift assay (CETSA) analysis of protein thermal stability in rat lung tissue samples following oral administration or *in vitro* incubation with daphnetin. (A, B) Effect of daphnetin administration via oral gavage (A) or *in vitro* incubation (B) on the thermal stability of Nicotinamide adenine dinucleotide phosphate hydrogen (NAD(P)H) quinone oxidoreductase 1 (NQO1) protein. (C, D) Effect of daphnetin on 5-oxoprolinase (OPLAH) protein stability following oral administration (C) or *in vitro* incubation (D). (E, F) Effect of daphnetin on nuclear factor erythroid 2-related factor 2 (NRF2) protein stability after oral administration (E) or *in vitro* incubation (F). (G, F) Effect of daphnetin on arginase 1 (ARG1) protein stability following oral administration (G) or *in vitro* incubation (H). T_m_: Melting temperature.

**Fig. S7.** Molecular docking, molecular dynamics simulations, and virtual alanine scanning analysis. (A) Molecular docking pose of daphnetin with Nicotinamide adenine dinucleotide phosphate hydrogen (NAD(P)H) quinone oxidoreductase 1 (NQO1). (B) Molecular docking conformation of daphnetin with 5-oxoprolinase (OPLAH). (C, D) Molecular docking mode of dicoumarin with NQO1. (E) Virtual alanine scanning analysis of the predicted binding site between OPLAH-daphnetin complexe. (F, G) Root mean square deviation (RMSD) trajectories of the NQO1-daphnetin (F) and OPLAH-daphnetin (G) complexes during the 100 ns simulation. (H, I) Root mean square fluctuation (RMSF) profiles of the NQO1-daphnetin (H) and OPLAH-daphnetin (I) complexes. (J, K) Hydrogen bond number dynamics for the NQO1-daphnetin (J) and OPLAH-daphnetin (K) complexes. (L, M) Radius of gyration (Rg) analysis of the NQO1-daphnetin (L) and OPLAH-daphnetin (M) complexes. (N, O) Solvent-accessible surface area (SASA) of the NQO1-daphnetin (N) and OPLAH-daphnetin (O) complexes.
